# Reduction of nemo-like kinase increases lysosome biogenesis and ameliorates TDP-43–related neurodegeneration

**DOI:** 10.1172/JCI138207

**Published:** 2023-08-15

**Authors:** Leon Tejwani, Youngseob Jung, Hiroshi Kokubu, Sowmithra Sowmithra, Luhan Ni, Changwoo Lee, Benjamin Sanders, Paul J. Lee, Yangfei Xiang, Kimberly Luttik, Armand Soriano, Jennifer Yoon, Junhyun Park, Hannah H. Ro, Hyoungseok Ju, Clara Liao, Sofia Massaro Tieze, Frank Rigo, Paymaan Jafar-Nejad, Janghoo Lim

**Affiliations:** 1 Interdepartmental Neuroscience Program,; 2 Department of Neuroscience, and; 3 Department of Genetics, Yale School of Medicine, New Haven, Connecticut, USA.; 4 Ionis Pharmaceuticals, Carlsbad, California, USA.; 5 Yale College, New Haven, Connecticut, USA.; 6 Program in Cellular Neuroscience, Neurodegeneration and Repair, and; 7 Yale Stem Cell Center, Yale School of Medicine, New Haven, Connecticut, USA.

**Keywords:** Genetics, Neuroscience, Lysosomes, Mouse models, Neurodegeneration

## Abstract

Protein aggregation is a hallmark of many neurodegenerative disorders, including amyotrophic lateral sclerosis (ALS). Although mutations in *TARDBP*, encoding transactive response DNA-binding protein 43 kDa (TDP-43), account for less than 1% of all ALS cases, TDP-43–positive aggregates are present in nearly all ALS patients, including patients with sporadic ALS (sALS) or carrying other familial ALS–causing (fALS-causing) mutations. Interestingly, TDP-43 inclusions are also present in subsets of patients with frontotemporal dementia, Alzheimer’s disease, and Parkinson’s disease; therefore, methods of activating intracellular protein quality control machinery capable of clearing toxic cytoplasmic TDP-43 species may alleviate disease-related phenotypes. Here, we identify a function of nemo-like kinase (Nlk) as a negative regulator of lysosome biogenesis. Genetic or pharmacological reduction of *Nlk* increased lysosome formation and improved clearance of aggregated TDP-43. Furthermore, Nlk reduction ameliorated pathological, behavioral, and life span deficits in 2 distinct mouse models of TDP-43 proteinopathy. Because many toxic proteins can be cleared through the autophagy/lysosome pathway, targeted reduction of *Nlk* represents a potential approach to therapy development for multiple neurodegenerative disorders.

## Introduction

In the majority of neurodegenerative diseases, one or more proteins aggregate over the course of disease progression and, in some cases, are thought to play a central role in pathogenesis. In the case of amyotrophic lateral sclerosis (ALS), cytoplasmic inclusions of transactive response DNA-binding protein 43 kDa (TDP-43) are observed in approximately 97% of all patients, including patients with sporadic ALS (sALS) or familial ALS (fALS) (1–3), as well as in subsets of patients with frontotemporal dementia (FTD) (1, 3), Alzheimer’s disease (4), and Parkinson’s disease (5). This aberrant accumulation of cytoplasmic TDP-43 is thought to underlie gain of toxic functions and exacerbate loss of nuclear function pathogenic mechanisms (6); therefore, preventing the formation or promoting the clearance of these inclusions may be an effective therapeutic approach for TDP-43 proteinopathies.

We have previously reported that nemo-like kinase (Nlk) is a proline-directed serine/threonine kinase capable of interacting with and phosphorylating several neurodegenerative disease–causing proteins, namely, ataxin-1 and androgen receptor (AR), the proteins whose polyglutamine repeat expansion are causal for spinocerebellar ataxia type 1 (SCA1) and spinal and bulbar muscular atrophy (SBMA), respectively (7, 8). Genetic reduction of *Nlk* in animal models for SCA1 and SBMA alleviates several disease-related phenotypes, and this partial rescue was previously attributed to decreased phosphorylation of 2 Nlk substrates, ataxin-1 and AR, at key residues involved in the pathogenic mechanisms (7, 8). Although it is known that Nlk directly interacts with several other proteins that are involved in central nervous system disorders (9) as well as indirectly to affect inflammation through altered NF-κB transcriptional activity (10), the physiological role of Nlk and the general effects of its modulation in the adult nervous system have not yet been comprehensively examined.

## Results

### Nlk negatively regulates the lysosome.

**Figure 1:**
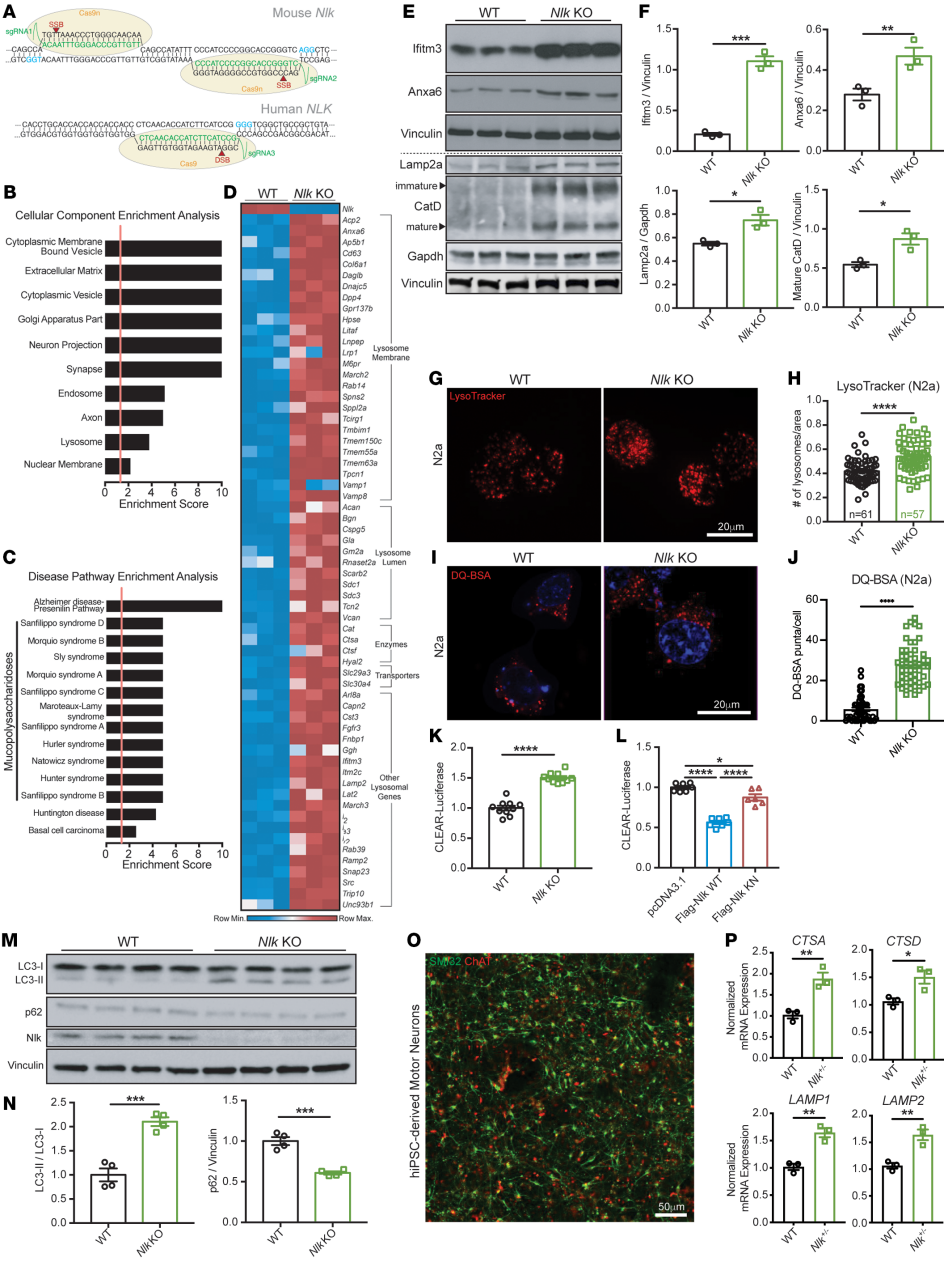
Reduction of *Nlk* increases lysosome biogenesis in vitro. (**A**) Schematic of dual-guide RNA targeting of Cas9 nickase (Cas9n) to *Nlk* for generation of isogenic *Nlk* KON 2a cells and *NLK^+/-^* hiPSCs. (**B** and **C**) Enrichment analyses for all significantly differentially expressed genes from RNA-Seq of isogenic WT and *Nlk* KO N2a cells. Significant enrichment scores of 1.3 (FDR adjusted *P* value *q* < 0.05) are designated by vertical red lines. (**D**) Heatmap of significantly upregulated lysosomal genes from RNA-Seq (*n* = 3 biological replicates). (**E** and **F**) Western blot validation (**E**) confirmed RNA-Seq results, quantified in **F** (*n* = 3 biological replicates). Blot lanes separated by the dotted line were run on lysates from distinct experimental replicates and normalized to their respective housekeeping proteins. (**G** and **H**) Representative LysoTracker images of N2a cells (**G**), quantified in **H**, demonstrated increased lysosome number in *Nlk* KO cells (WT, *n* = 61 cells; *Nlk* KO, *n* = 57 cells). (**I** and **J**) Representative images of functional lysosomes in N2a cells incubated with DQ BSA (**I**), quantified in **J** (WT, *n* = 50 cells; *Nlk* KO, *n* = 50 cells). (**K** and **L**) CLEAR-luciferase assay in *Nlk* KO N2a cells (**K**) or WT N2a cells transfected with WT or KN Nlk (**L**) demonstrated Nlk kinase activity suppresses CLEAR network transcription (**K**, *n* = 10 replicates from 2 different *Nlk* KO clones; **L**, *n* = 7 replicates from 2 experimental repeats). (**M** and **N**) Western blots (**M**) showed increased LC3-II/LC3-I and decreased p62 levels in *Nlk* KO N2a cells, quantified in **N** (*n* = 4 biological replicates). (**O**) Representative immunostaining images of hiPSC-derived motor neurons. (**P**) qPCR showing *NLK^+/–^* iPSC–derived motor neurons expressed higher levels of *CTSA*, *CTSD*, *LAMP1*, and *LAMP2* compared with isogenic controls (*n* = 3 biological replicates from distinct motor neuron differentiations). Two-tailed *t* tests (**F**, **H**, **J**, **K**, **N**, and **P**) or 1-way ANOVA (**L**) analyses were performed, and data are represented as mean ± SEM. *P < 0.05; **P < 0.01; ***P < 0.001; ****P < 0.0001.

We first sought to interrogate the cellular functions of Nlk beyond its role in altering phosphorylation levels of ataxin-1 and AR. To this end, we utilized dual-guide RNAs to target Cas9 nickase to *Nlk* in Neuro2a (N2a) cells, generating 2 offset single-stranded nicks for error-prone nonhomologous end-joining repair (Figure 1A) (11). Due to the polyploidy of N2a cells, we assessed the efficacy of mutagenesis by examining residual Nlk protein levels (Supplemental Figure 1A; supplemental material available online with this article; https://doi.org/10.1172/JCI138207DS1; all raw, uncropped Western blots are available as supplemental material). Individual clones were classified by amount of intact Nlk protein, and to ensure that the phenotypes observed were a result of Nlk depletion and not spurious artifacts of the CRISPR/Cas9 mutagenesis, multiple distinct clones with undetectable Nlk levels (*Nlk* KO) were expanded and used for further experimentation (Supplemental Figure 1B). RNA-Seq was performed on *Nlk-*KO cells and WT isogenic controls (Supplemental Figure 2, A and B, and Supplemental Table 1). Interestingly, gene ontology analyses on all differentially expressed genes revealed an enrichment for transcriptional changes related to multiple cytoplasmic membrane-bound vesicles, including endosomes and lysosomes (Figure 1B), but not to stress granules, which are membraneless structures implicated in ALS pathogenesis (12) (Supplemental Figure 2, C and D). Additionally, lysosomal storage disorders were among the significant disease pathways highlighted by the enrichment analysis (Figure 1C).

Because of the overrepresentation of lysosome-associated transcripts in the *Nlk*-KO data set (Figure 1D and Supplemental Table 2), some of which we validated at the protein level (Figure 1, E and F), we sought to identify the functional consequences of Nlk reduction on lysosome biogenesis and function. We determined that lysosome number was increased in *Nlk-*KO N2a cells compared with that in isogenic controls (Figure 1, G and H, and Supplemental Figure 3, A and B). Transfection of exogenous Nlk suppressed this increase in lysosome number in a kinase activity–dependent manner (Supplemental Figure 3, C and D), as kinase-negative (KN) Nlk did not affect lysosome count. Addition of DQ BSA, a self-quenched dye that becomes fluorescent upon proteolysis by lysosomal hydrolases, revealed increased fluorescence in the absence of Nlk (Figure 1, I and J), suggesting not only that the number of lysosomes increases, but that their proteolytic functionality does as well. Furthermore, transfection of a 4XCLEAR-luciferase reporter (13) confirmed that *Nlk* KO promoted transcription of genes belonging to the coordinated lysosomal enhancement and regulation (CLEAR) network of autophagy and lysosomal genes (14, 15) (Figure 1K and Supplemental Figure 3E), while overexpression of Nlk suppressed 4XCLEAR-luciferase reporter activity in a kinase activity–dependent manner (Figure 1L). Using a tandem fluorescent-tagged RFPGFP-LC3 reporter (16) (Supplemental Figure 3F), we observed that Nlk overexpression decreased the number of autophagolysosomes in a kinase activity–dependent manner (Supplemental Figure 3, G and H). The LC3-II to LC3-I ratio was increased, with a concomitant decrease in p62 in *Nlk-*KO cells (Figure 1, M and N), while overexpression of WT Nlk led to an accumulation of p62 (Supplemental Figure 3I). These data suggest that Nlk negatively regulates autophagy-lysosome flux not only by suppressing lysosomal degradative capacity, but also by suppressing autophagosome formation/maturation, however, to a lesser extent than the effects on lysosomal function.

Finally, we assessed levels of several transcripts encoding proteins critical for lysosome biogenesis and/or function (not limited to differentially expressed genes from our N2a RNA-Seq data set) in human motor neurons differentiated from CRISPR/Cas9-mutated *NLK^+/–^* induced pluripotent stem cells (iPSCs) (Figure 1A and Supplemental Figure 4). These experiments demonstrated that NLK-deficient motor neurons expressed higher levels of *CTSA*, *CTSD*, *LAMP1*, and *LAMP2* compared with isogenic WT cells (Figure 1, O and P). Collectively, these data demonstrate that *Nlk* reduction increases autophagic flux and cargo degradation by increasing lysosome biogenesis, while *Nlk* overexpression impairs lysosomal degradation of autophagy substrates.

### Nlk destabilizes nuclear Tfeb.

**Figure 2:**
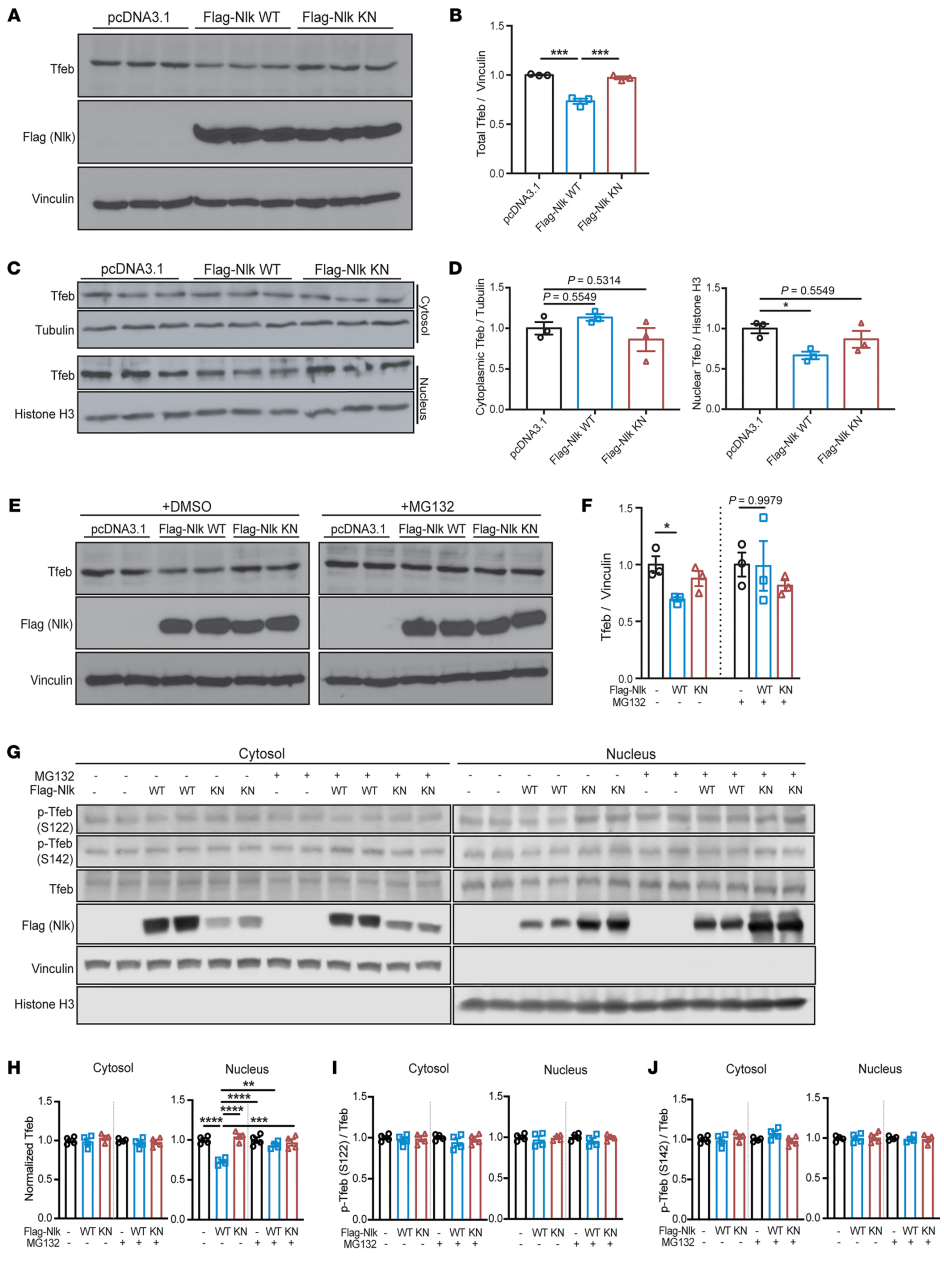
Nlk destabilizes nuclear Tfeb via proteasomal degradation. (**A** and **B**) Western blots of N2a whole-cell protein lysates following transfection with Flag-Nlk WT or Flag-Nlk KN (**A**). Tfeb levels decreased upon Nlk overexpression in a kinase activity–dependent manner, quantified in **B** (*n* = 3 biological replicates). (**C** and **D**) Western blots of WT N2a cells transfected with Flag-Nlk WT or Flag-Nlk KN showed a kinase activity–dependent decrease of nuclear Tfeb levels (**C**), quantified in **D** (*n* = 3 biological replicates). (**E** and **F**) Western blots of N2a whole-cell protein lysates following transfection with Flag-Nlk WT or Flag-Nlk KN, with DMSO vehicle or MG132 treatment (**E**). Tfeb levels decreased upon Nlk overexpression in a kinase activity–dependent manner, but this destabilization was not observed in the presence of the proteasome inhibitor MG132, quantified in **F** (*n* = 3 biological replicates). (**G**–**J**) Western blots of subcellular fractionated N2a protein lysates following transfection with Flag-Nlk WT or Flag-Nlk KN, with DMSO or MG132 treatment (**G**). Only nuclear Tfeb levels were reduced upon Nlk overexpression in a kinase activity–dependent manner, and this decrease was dependent on the proteasomal degradation pathway, quantified in **H**. Phosphorylation of Tfeb at S122 and S142 was not affected by Nlk overexpression, quantified in **I** and **J** (*n* = 4 biological replicates). One-way ANOVA analyses (**B**, **D**, **F**, and **H**–**J**) were performed, and data are represented as mean ± SEM. *P < 0.05; **P < 0.01; ***P < 0.001; ****P < 0.0001.

Several transcription factors have been reported to be involved in the transcriptional regulation of a large number of lysosomal genes, and among these, Tfeb has been described as a key master regulator of lysosome gene expression and function (14, 15, 17). To determine whether Nlk exerts its control of lysosome-associated genes through altering Tfeb, we assessed Tfeb in N2a cells in which WT or KN Nlk was overexpressed. In whole-cell lysates, total Tfeb levels were reduced with Nlk overexpression in a kinase activity–dependent manner (Figure 2, A and B). Because the ability of Tfeb to induce lysosomal gene expression is highly dependent on its translocation to the nucleus (18), we next isolated nuclear and cytosolic fractions to assess changes in the subcellular localization of Tfeb. Interestingly, overexpression of Nlk induced a nucleus-specific reduction in Tfeb levels, with no changes in cytosolic levels (Figure 2, C and D), suggesting the nuclear depletion of Tfeb was not attributed to its cytosolic retention. Furthermore, because the reduction in Tfeb was compartment specific (Figure 2, C and D) and *Tfeb* transcript levels were not significantly altered in Nlk-deficient N2a cells (Supplemental Table 1), this regulation was not a result of transcriptional changes of *Tfeb* itself.

Because the nuclear reduction of Tfeb appeared to be posttranslational and not due to a shuttling deficiency from the cytosol, we next examined the contribution of the ubiquitin-proteasome system (UPS), a major protein catabolism pathway active in both the cytosol and nucleus involved in the turnover of short- and long-lived proteins (19). Although overall proteasome activity was not affected by Nlk overexpression (Supplemental Figure 3J), as determined using a Ub^G76V^-GFP reporter (20), treatment with the proteasome inhibitor MG132 normalized total Tfeb levels following Nlk overexpression (Figure 2, E and F). Subcellular fractionation confirmed that MG132 treatment specifically restored nuclear Tfeb levels without affecting cytosolic Tfeb levels in cells in which Nlk was overexpressed (Figure 2, G and H). Collectively, these data demonstrate that Nlk-induced Tfeb destabilization is specifically dependent on the nuclear proteasome.

Phosphorylation of Tfeb at specific residues by various kinases, the best documented being mTOR, has been ascribed to be a major mechanism that governs subcellular localization and activity of Tfeb (18, 21–24). To determine whether this mechanism of Tfeb regulation by Nlk could similarly be mediated through a direct effect of Nlk on mTOR signaling, we examined several mTOR targets. We determined that *Nlk* KO or overexpression of Nlk in N2a cells did not affect 4E-BP1, S6, or ULK1 phosphorylation (Supplemental Figure 5), suggesting the observed effect on Tfeb by Nlk is not through altering overall mTOR activity. Furthermore, no changes in phosphorylation of Tfeb itself at S122 or S142 were observed (Figure 2, G, I, and J). Overall, these data reveal a mechanism for the regulation of Tfeb levels by Nlk and a component of the crosstalk between the transcriptional control of the lysosome and the UPS.

### Nlk regulates TDP-43 levels.

**Figure 3:**
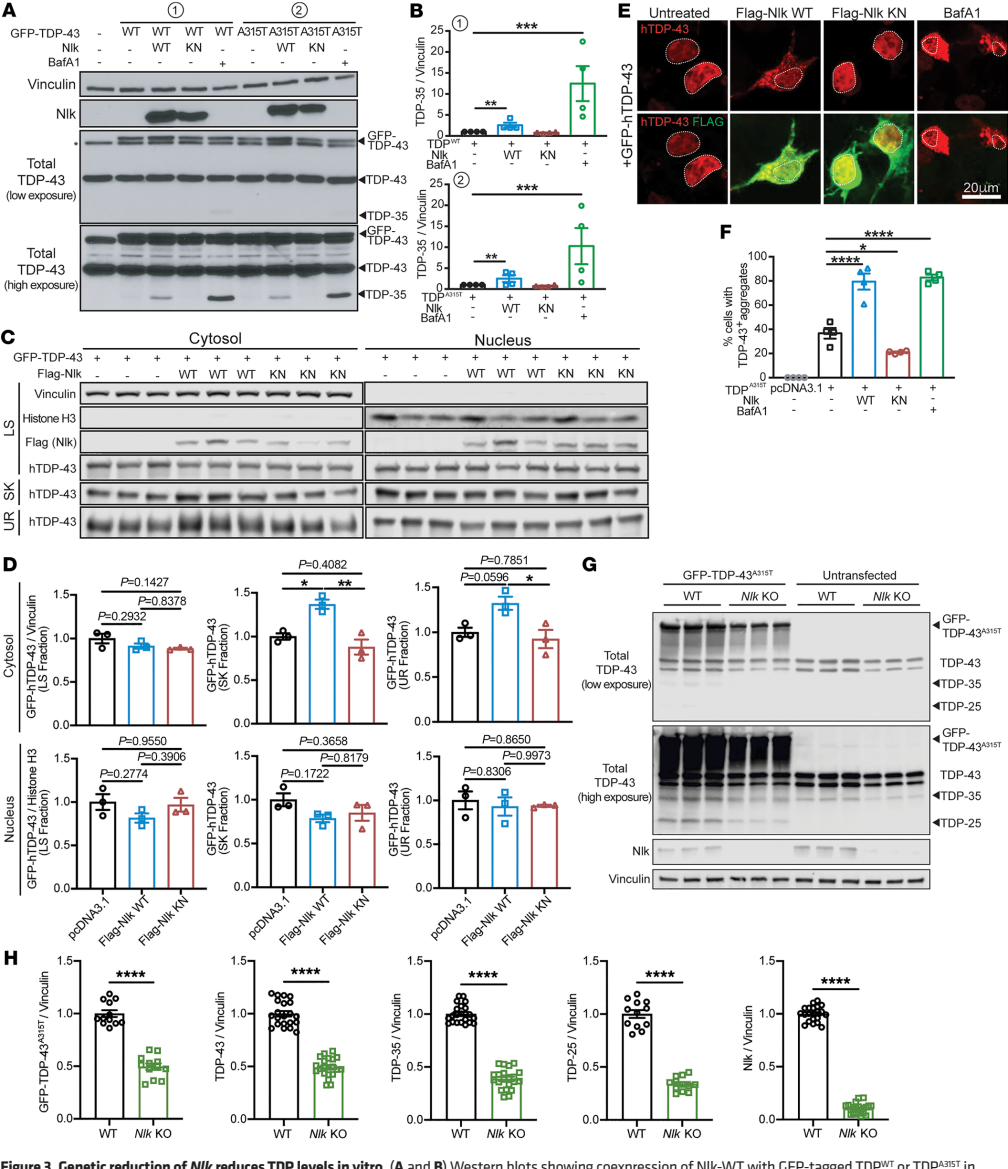
Genetic reduction of *Nlk* reduces TDP levels in vitro. (**A** and **B**) Western blots showing coexpression of Nlk-WT with GFP-tagged TDP^WT^ or TDP^A315T^ in NSC-34 cells (**A**) increased levels of TDP-35, while Nlk-KN did not. V-ATPase inhibitor BafA1 treatment similarly increased TDP-35 levels in the absence of Nlk overexpression to an even greater extent. Quantification shows TDP-35 levels in **B** (*n* = 4 biological replicates). (**C** and **D**) Western blots of sequentially extracted proteins based on detergent solubility from subcellular fractionated NSC-34 cells (**C**). Quantification of TDP-43 species shows Nlk WT overexpression increased cytosolic insoluble TDP-43 (sarkosyl and urea fractions) (**D**) (*n* = 3 biological replicates). LS, low salt; SK, sarkosyl; UR, urea. (**E** and **F**) Representative immunostaining of NSC-34 cells cotransfected with GFP-tagged TDP-43 and Nlk showing that WT Nlk increased formation of GFP-positive cytosolic TDP-43 aggregates (**E**), quantified in **F**. Nuclei are outlined with dotted lines (*n* = 4 biological replicates). (**G** and **H**) Western blots of cell lysates from WT or *Nlk* KO N2a cells transfected with GFP–TDP-43^A315T^ (**G**), quantified in **H** (*n* = 12–24 replicates pooled from 4 individual experiments). Levels of exogenous GFP–TDP-43^A315T^, endogenous TDP-43, and truncated fragments of TDP-43 were significantly reduced. One-way ANOVA analyses (**B**, **D**, and **F**) or 2-tailed *t* tests (**H**) were performed to compare all listed genotypes/treatments unless otherwise noted, and data are represented as mean ± SEM. *P < 0.05; **P < 0.01; ***P < 0.001; ****P < 0.0001.

The autophagy/lysosome pathway (ALP) is 1 of 2 primary systems for intracellular protein degradation and has been implicated as being defective in some forms of ALS (25, 26). In most neurodegenerative disorders, including ALS, one or more proteins aberrantly accumulate, and these aggregates are typically associated with disease pathogenesis. Although the genetic basis underlying ALS is heterogeneous (2), ubiquitin-positive TDP-43 inclusions are present in nearly all ALS patients (1, 3). Because soluble and aggregated forms of TDP-43 and its cleavage products can be cleared through the ALP (27–30), we aimed to determine whether TDP-43 levels could be modulated through altering Nlk levels. Coexpression of TDP-43^WT^ or mutant TDP-43^A315T^, a fALS-causing variant of TDP-43 (31), with Nlk in NSC-34 cells induced a kinase activity–dependent increase in total TDP-43 and a 35 kDa TDP species, both of which also increased with lysosome inhibitor bafilomycin A1 (BafA1) (Figure 3, A and B). Whether this 35 kDa TDP species represents a cleavage product of TDP-43 or an activity-dependent short isoform of *TARDBP* (32) remains to be determined. Further analyses confirmed that the Nlk-induced increase in TDP-43 occurred specifically in the cytosol (Figure 3, C–F). Furthermore, sequential protein extraction based on detergent solubility (3) confirmed that Nlk overexpression resulted in the cytosolic accumulation of insoluble TDP-43 species (sarkosyl and urea fractions) and not soluble TDP-43 (low-salt fraction) (Figure 3, C and D). Conversely, *Nlk* reduction in N2a cells reduced protein levels of full-length and truncated fragments of TDP-43 (Figure 3, G and H). This reduction in TDP-43 was likely not a result of a decreased physical binding with Nlk or alterations in direct phosphorylation by Nlk, as Nlk did not physically interact with endogenous or overexpressed TDP-43 under the tested conditions (Supplemental Figure 6, A and B). Additionally, Nlk overexpression did not affect overall TDP-43 ubiquitination (Supplemental Figure 3K). Collectively, these data suggest an enhanced indirect clearance mechanism of pathological TDP-43 by Nlk reduction.

### Genetic reduction of Nlk extends TDP-43 mouse survival.

**Figure 4:**
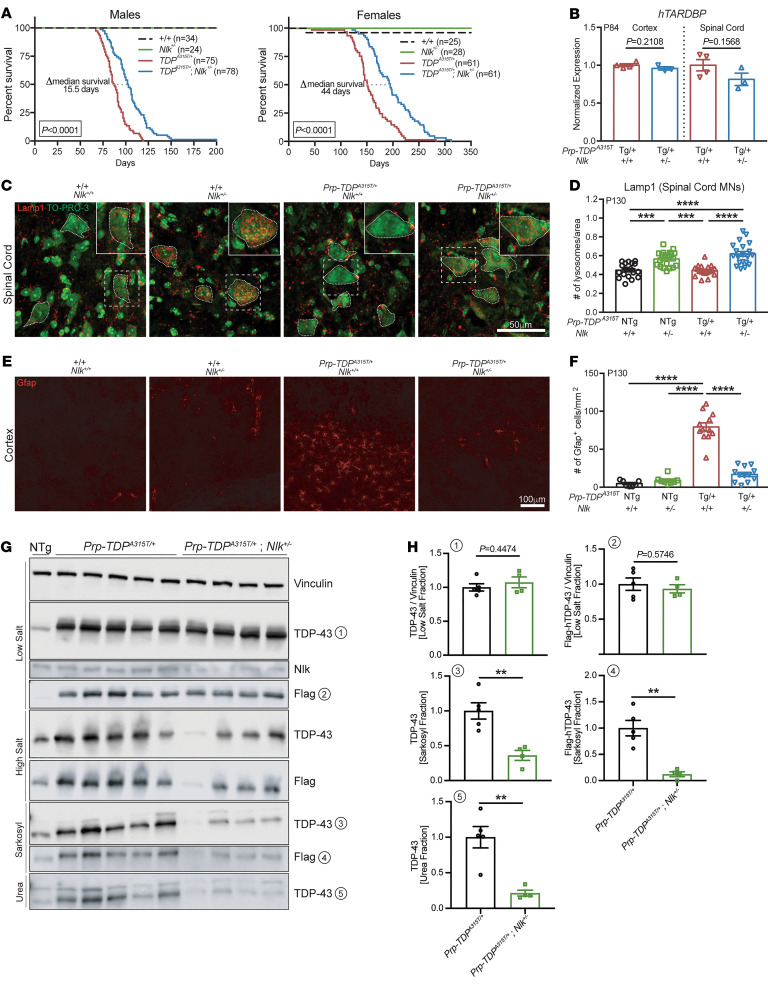
Genetic reduction of *Nlk* improves survival and pathology in TDP-43 mice. (**A**) Kaplan-Meier survival curves showing 50% genetic reduction of *Nlk* increased *Prp-TDP^A315T/+^* (Prp–TDP-43 Tg*)* male and female animal survival. Data are combined from 2 independent *Nlk* gene trap insertion lines. Sample sizes for each genotype are provided in the figure. Curves were compared by log-rank test. (**B**) A 50% genetic reduction of *Nlk* did not affect cortex or spinal cord *hTARDBP* mRNA levels in P84 *Prp-TDP^A315T/+^* mice (*Nlk^+/+^*, *n* = 4 animals; *Nlk^+/–^*, *n* = 3 animals). (**C**–**F**) Representative immunohistochemical images showing genetic reduction of *Nlk* increased Lamp1^+^ lysosomes in the soma (small dotted lines) of lumbar spinal cord motor neurons (**C**, quantified in **D**; *n* = 18–22 motor neurons from 3 animals per genotype) and rescued layer V astrogliosis (**E**, quantified in **F**; *n* = 12 sections from 3 animals per genotype) in P130 *Prp-TDP^A315T/+^* female mice. (**G** and **H**) Western blots of various forms of TDP-43 in fractions of female mouse spinal cords at P200 (**G**). Quantification of TDP-43 species shows levels of insoluble TDP-43 (sarkosyl and urea fractions) were significantly reduced with 50% genetic reduction of *Nlk* (**H**) (TDP-Tg, *n* = 5 animals; TDP-Tg/Nlk, *n* = 4 animals). Note that TDP-43^A315T/+^ protein is Flag tagged at the N-terminal end. Two-tailed *t* tests (**B** and **H**) or 1-way ANOVA analyses (**D** and **F**) were performed to compare all listed genotypes/treatments unless otherwise noted, and data are represented as mean ± SEM. NTg, nontransgenic; Tg, transgenic. **P < 0.01; ***P < 0.001; ****P < 0.0001.

We hypothesized that increasing lysosome biogenesis and TDP clearance through targeted Nlk reduction could modify pathogenesis in animal models of TDP-43 proteinopathies. To test this, we crossed 2 independent *Nlk* loss-of-function mouse lines (*Nlk^XN619/+^* and *Nlk^RRJ297/+^*) (7, 8), collectively referred to here as *Nlk^+/–^*, with transgenic mice expressing a fALS-causing mutant *hTARDBP^A315T^* harboring an N-terminal Flag tag under the control of a *Prp* promoter (33), referred to here as *Prp-TDP^A315T/+^*. Although male and female *Prp-TDP^A315T/+^* hemizygous mice display differences in survival that are likely due to sex differences in gastrointestinal complications that are associated with lethality (34), genetic reduction of *Nlk* by 50% increased median survival of male and female *Prp-TDP^A315T/+^* mice by about 20% (Figure 4A and Supplemental Figure 7, A and B), without altering *hTARDBP* transcript levels (Figure 4B). In addition to increasing overall survival, disease onset, which was defined as the first time point in which weight gain was no longer observed (35, 36), was delayed in male mice with a 50% genetic reduction of *Nlk* (Supplemental Figure 7C). Examination of P130 female mice revealed that genetic reduction of *Nlk* was able to increase Lamp1-positive lysosome number in motor neurons of the lumbar spinal cord in vivo (Figure 4, C and D). Additionally, astrogliosis in layer V of the motor cortex was reduced to near-control levels in *Prp-TDP^A315T/+^* mice with *Nlk* reduction (Figure 4, E and F).

Our in vitro experiments describing the effect of Nlk reduction on promoting lysosomal function and clearance of TDP-43 (Figure 1 and Figure 3), along with the confirmation that genetic reduction of *Nlk* increases lysosome gene expression and number in mouse and human iPSC-derived (hiPSC-derived) spinal cord motor neurons (Figure 1P and Figure 4, C and D), suggest that the therapeutic benefit we observed in vivo could be mediated through altering levels of aggregated TDP-43 in motor neurons. To determine whether *Nlk* reduction did indeed alter levels of aggregated TDP-43 in vivo, we sequentially isolated protein fractions from spinal cords of *Prp-TDP^A315T/+^* and *Nlk^+/-^; Prp-TDP^A315T/+^* mice. Biochemical analysis of TDP-43 confirmed that genetic *Nlk* reduction decreased overall TDP-43 levels in the more insoluble fractions (Figure 4, G and H). Importantly, *Nlk* reduction did not affect levels of soluble TDP-43 (Figure 4, G and H), suggesting a preservation of soluble TDP-43 to engage in its normal, critical functions. These data demonstrate that *Nlk* reduction in vivo causes an increase in lysosome biogenesis that, presumably through increasing clearance of toxic TDP-43 species, alleviates pathological changes and extends life span of *Prp-TDP^A315T/+^* hemizygous mice.

### Nlk ASOs improve TDP-43 mouse behavior.

**Figure 5:**
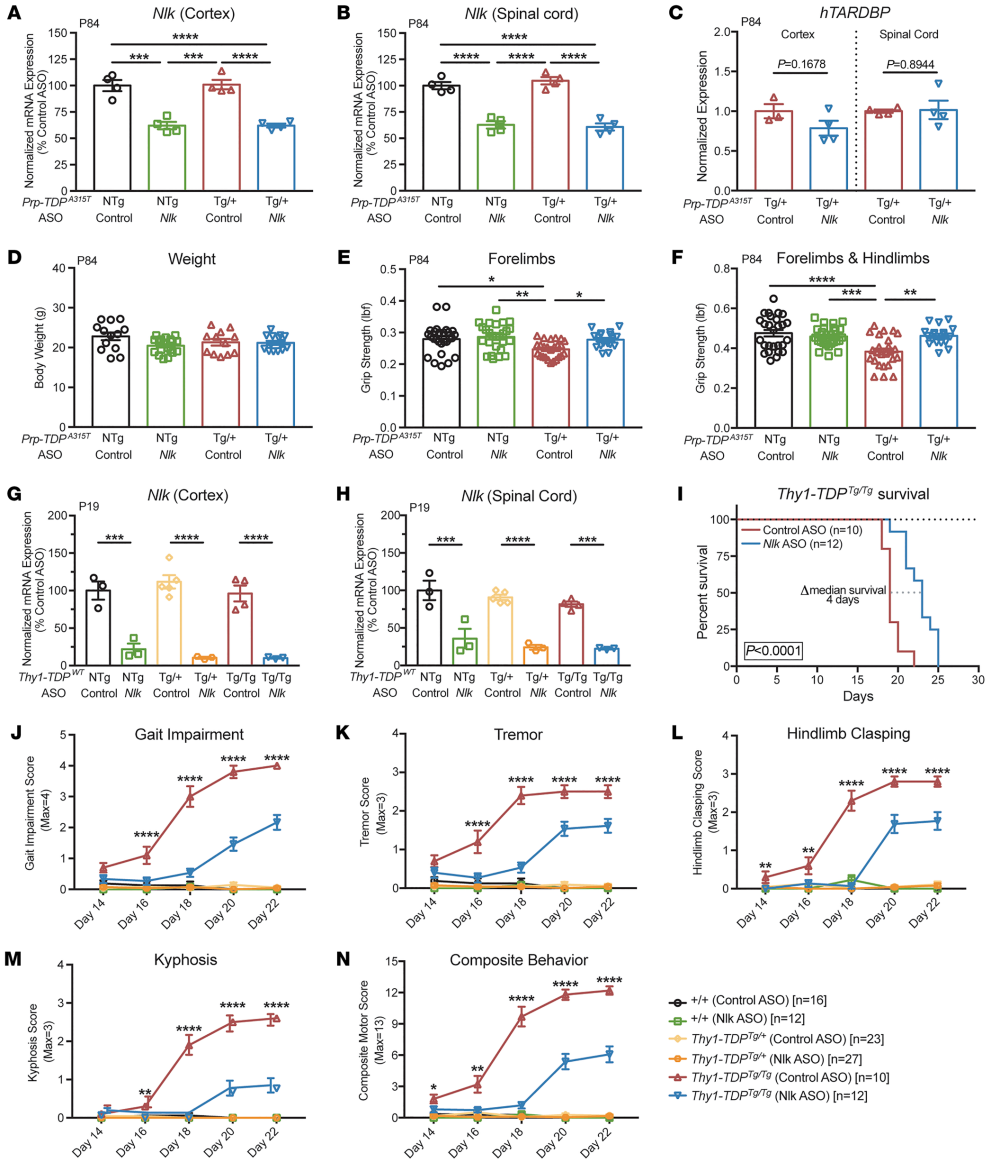
Pharmacological reduction of *Nlk* using ASOs improves behavior in 2 TDP-43 mouse models. (**A** and **B**) An i.c.v. injection of 10 μg of *Nlk* ASO at P1 resulted in a 40% reduction of *Nlk* mRNA at P84 in the cortex (**A**) and spinal cord (**B**) (*n* = 4 animals per condition). (**C**) ASO reduction of *Nlk* did not affect transcript levels of *hTARDBP* (*n* = 3–4 animals per condition). (**D**) Overall body weight was not affected by ASO administration (*n* = 12–19 animals per condition). (**E** and **F**) *Nlk* ASO administration at P1 rescued forelimb (**E**) and forelimb with hind limb (**F**) grip-strength deficits in *Prp-TDP^A315T/+^* mice at P84 (*n* = 18–27 animals per condition). (**G** and **H**) Injection of 10 μg of *Nlk* ASO at P1 resulted in a 90% and 75% reduction of *Nlk* mRNA at P19 in the cortex (**G**) and spinal cord (**H**), respectively (*n* = 3–5 animals per condition). (**I**) Kaplan-Meier survival curves showing administration of 10 μg *Nlk* ASO increased *Thy1-TDP^Tg/Tg^* animal survival (*n* = 10–12 animals per condition). Curves were compared by log-rank test. (**J**–**N**) *Nlk* ASO administration reduced gait impairment (**J**), tremor (**K**), hind limb clasping (**L**), kyphosis (**M**), and composite motor score (**N**) in *Thy1-TDP^Tg/Tg^* animals between P14 and P22 (*n* = 10–28 animals per condition). One-way ANOVA analyses (**A**–**H** and **J**–**N**) were performed to compare all listed genotypes/treatments per day unless otherwise noted, and data are represented as mean ± SEM. *P < 0.05; **P < 0.01; ***P < 0.001; ****P < 0.0001.

To validate the approach of *Nlk* reduction for neurodegenerative diseases in a more translationally relevant manner, we generated antisense oligonucleotides (ASOs) targeting *Nlk*. ASOs are short oligonucleotides that hybridize to complementary transcripts with high specificity and target them for RNase H–mediated degradation (37). The chemically modified backbone and bases minimize immunogenicity and enhance ASO stability, enabling persistent knockdown of a target transcript for prolonged periods of time. In recent years, ASO-based approaches have shown promise in preclinical models and human clinical trials for various motor neuron diseases (38–41), spinal muscular atrophy (42), and Huntington’s disease (43). Injections of *Nlk* ASOs i.c.v. at P1 led to a dose-dependent reduction of *Nlk* mRNA levels in the cortex and spinal cord (Figure 5, A and B, and Supplemental Figure 8, A and B) with no overt astrogliosis or microgliosis at P84 (Supplemental Figure 8, C–F). Administration of *Nlk* ASOs to *Prp-TDP^A315T/+^* mice did not affect levels of *hTARDBP* transcript (Figure 5C) or overall body weight (Figure 5D).

Based on our dosing experiments (Supplemental Figure 8, A and B), we selected a dose of 10 μg *Nlk* ASO, which produced a robust and persistent knockdown efficiency of *Nlk* by 40% at P84 following a single P1 injection (Figure 5, A and B). We began by assessing the effect of *Nlk* ASOs on motor function at P84, a time point at which *Prp-TDP^A315T/+^* mice display brain and spinal cord pathology (33). *Prp-TDP^A315T/+^* mice displayed reduced forelimb and forelimb with hind limb grip strength compared with nontransgenic controls, and these deficits were restored to control levels in transgenic animals that received *Nlk* ASOs (Figure 5, E and F).

To confirm the benefit of *Nlk* reduction on motor deficits associated with TDP-43 accumulation, we turned to an additional transgenic mouse model that overexpresses WT *hTARDBP* under the control of a *Thy1* promoter (44), referred to here as *Thy1-TDP^Tg/Tg^*. Compared with the *Prp-TDP^A315T/+^* mice, these animals display much more severe behavioral deficits, characterized by a rapidly progressing motor impairment and eventual paralysis (necessitating euthanasia at a humane end point) when bred to give rise to homozygous progeny (44). P1 i.c.v. delivery of 10 μg of *Nlk* ASO reduced *Nlk* mRNA levels at P19 by 90% and 75% in the cortex and spinal cord, respectively (Figure 5, G and H). Median life span in *Thy1-TDP^Tg/Tg^* animals injected with *Nlk* ASOs at P1 was increased by 21% compared with that of control ASO-injected animals (Figure 5I). Additionally, motor behavior was scored as previously described (39, 45) and *Nlk* reduction ameliorated gait impairment, tremor, hind limb clasping, and kyphosis between P14 and P22 (Figure 5, J–N, Supplemental Figure 9, and Supplemental Video 1). Finally, because altering diet may affect the course of disease in transgenic ALS mouse models (46), we raised an independent cohort of *Thy1-TDP^Tg/Tg^* animals on a high-fat, gel-food diet and observed a similar improvement in motor behavior with a greater extension of survival with P1 *Nlk* ASO injections (Supplemental Figure 10).

### Nlk ASOs reduce TDP-43–related pathology.

**Figure 6:**
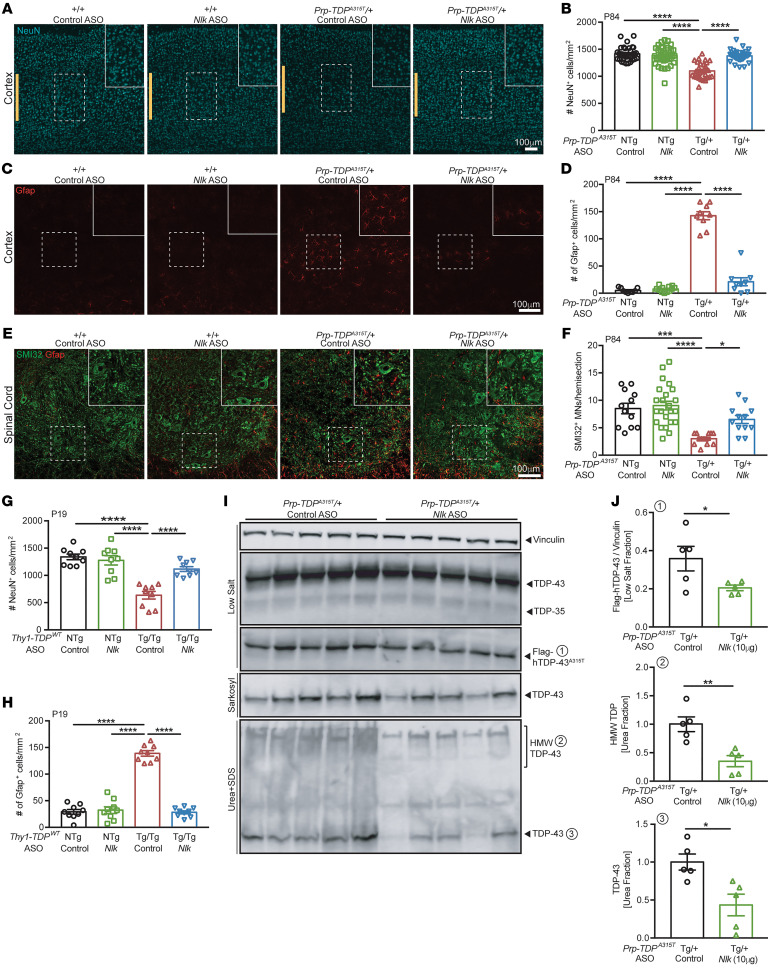
Pharmacological reduction of *Nlk* using ASOs decreases TDP-43 levels in vivo and ameliorates pathology in 2 TDP-43 mouse models. (**A**–**F**) Administration of 10 μg of *Nlk* ASO at P1 rescued loss of layer V cortical neurons (**A**, quantified in **B**; *n* = 29–39 sections from 4–5 animals per genotype; marked by yellow bars), layer V astrogliosis (**C**, quantified in **D**; *n* = 9–12 sections from 3–4 animals per genotype), and lumbar spinal cord motor neuron loss (**E**, quantified in **F**; *n* = 12–21 sections from 3–5 animals per genotype) in *Prp-TDP^A315T/+^* male mice at P84. (**G** and **H**) 10 μg of *Nlk* ASO at P1 rescued loss of layer V cortical neurons (**G**; *n* = 9 sections from 3 animals per genotype) and layer V astrogliosis (**H**; *n* = 9 sections from 3 animals per genotype) in *Thy1-TDP^Tg/Tg^* mice at P19. (**I** and **J**) Western blots of various forms of TDP-43 in fractions of male mouse spinal cords at P84. Quantification of TDP species from (**I**) shows levels of Flag-hTDP-43 (low-salt fraction), total TDP-43 (urea fraction), and high-molecular weight TDP (urea fraction) were significantly reduced in *Nlk* ASO–injected animals (**J**) (*n* = 5 animals per condition). One-way ANOVA analyses (**B**, **D**, **F**, **G**, and **H**) or 2-tailed *t* tests (**J**) were performed to compare all listed genotypes/conditions unless otherwise noted, and data are represented as mean ± SEM. *P < 0.05; **P < 0.01; ***P < 0.001; ****P < 0.0001.

We next examined the effect of pharmacological reduction of *Nlk* on pathological and biochemical changes in TDP-43 transgenic mice. *Prp-TDP^A315T/+^* mice displayed a loss of about 22% of layer V neurons of the motor cortex at P84 with an accompanying astrogliosis, both of which could be normalized to near control levels with a single P1 injection of 10 μg *Nlk* ASO (Figure 6, A–D, and Supplemental Figure 11, A and B). Similarly, loss of lumbar spinal cord SMI32-positive ventral horn motor neurons was mitigated with *Nlk* reduction (Figure 6, E and F, and Supplemental Figure 11C). The pathological rescue with *Nlk* ASOs was also observed in P19 *Thy1-TDP^Tg/Tg^* animals, as layer V cortical neuron loss and astrogliosis were prevented (Figure 6, G and H, and Supplemental Figure 11, D and E).

To confirm that *Nlk* reduction using ASOs can also affect levels of aggregated TDP-43 in vivo, similarly to genetic *Nlk* reduction (Figure 4, G and H), we examined spinal cord protein lysates of male P84 *Prp-TDP^A315T/+^* mice that had been injected with control or *Nlk* ASOs at P1. Consistent with the results from TDP-43 mice, in which *Nlk* was genetically reduced (Figure 4, G and H), *Nlk* reduction using ASOs also decreased overall TDP-43 levels in the more insoluble fractions without affecting soluble WT TDP-43 (Figure 6, I and J). Collectively, these data demonstrate that *Nlk* reduction can confer beneficial therapeutic effects in multiple murine models of protein aggregation.

## Discussion

The inability to adequately clear aggregation-prone proteins is a common phenomenon across neurodegenerative disorders. In ALS, a vast majority of patients display toxic cytoplasmic TDP-43–positive inclusions, regardless of sporadic or familial etiology. Therapeutic approaches aimed at preventing inclusion formation or enhancing clearance are viable strategies; however, methods that globally reduce TDP-43 levels are unfavorable, as TDP-43 serves essential cellular functions (6). On the other hand, increasing activity of cellular machinery capable of clearing cytoplasmic species of aggregated TDP-43, such as the ALP, may be a more effective approach, as this may reduce toxic effects in the cytoplasm while also potentially restoring the proper distribution of TDP-43 to the nucleus (47).

Here, we have identified Nlk as a negative regulator of the lysosome, whose modulation can affect transcription of lysosome-associated genes and therefore alter the capacity to clear disease-associated aggregated proteins, such as TDP-43, through the ALP. To test the therapeutic potential of this approach, we employed genetic and pharmacological tools to specifically reduce *Nlk* in 2 mouse models of TDP-43 proteinopathy and observed substantial life span, behavioral, biochemical, and pathological rescue. Furthermore, we have identified a molecular mechanism for the regulation of Tfeb through the nuclear proteasome. It is possible that Nlk reduction also affects additional target molecules in parallel with Tfeb, such as other transcription factors that may directly alter lysosome gene expression. This study warrants further investigation into these downstream and parallel molecules, which may reveal targets of interest that confer greater precision when designing therapeutic agents.

We have previously reported that NLK phosphorylates AR and ataxin-1, the proteins mutated to cause SBMA and SCA1, respectively, and that phosphorylation of these proteins contributes to pathogenicity (7, 8). Rather than a direct effect of altering phosphorylation of the disease-causing protein, the mechanism of therapeutic benefit by Nlk reduction described here is an indirect one likely mediated through increased lysosomal clearance of aggregated TDP-43, as there is no evidence for a physical interaction between NLK and TDP-43. Possibly due to the indirect nature of this interaction between NLK and TDP-43 through lysosome gene expression, NLK has not been previously identified in genetic screens of modulators of TDP-43 toxicity. It is possible that the role of NLK as a negative regulator of lysosome biogenesis described here may, in part, contribute to the amelioration of disease phenotypes upon genetic reduction of *Nlk* in SCA1 and SBMA animals, beyond simply altering phosphorylation of the toxic protein. A further dissection of the effect of these direct and indirect pathways on suppressing mutant ataxin-1 and AR toxicity could provide critical insights into the role of Nlk in neurodegenerative diseases and whether there is a unifying protective effect of Nlk reduction.

The therapeutic benefits of Nlk reduction on motor behavior, neurodegeneration and its associated astrogliosis, and survival of 2 independent TDP-43 animal models provide strong proof-of-principle evidence for this approach in TDP-43 proteinopathies, both in instances in which *TARDBP* mutations are present (a subset of ALS patients) and when *TARDBP* mutations are not present but TDP-43 aggregation occurs. Although human genetics studies have revealed ALS-causing mutations in numerous genes that converge on the ALP (48, 49), the animals used in the present study have not been reported to display overt impairments in autophagy/lysosome function, likely due to the aggressive disease progression in these models that contributes to premature lethality prior to robust lysosomal dysfunction that may eventually be induced by aberrant TDP-43 aggregation (50). However, a comprehensive examination of lysosomal health and function (beyond immunostaining for a single lysosomal membrane marker) in these animal models of TDP-43 proteinopathy is necessary to conclusively determine whether lysosomal dysfunction is present or not. Regardless, promoting lysosomal biogenesis in animal models of TDP-43 proteinopathy via the reduction of Nlk appears to have a beneficial impact on nearly all aspects of disease pathology, as demonstrated in the in vivo studies presented here.

Because genetic and pharmacological reduction of *Nlk* occurred either prenatally or at P1 in our animal models, respectively, the observed improvements may be attributed to alterations in the disease onset and/or progression. Our findings warrant a more thorough future examination of pathological alterations comparing animals at the onset, early disease, and late stages of disease to definitively determine whether disease progression can also be altered by Nlk reduction. It is important to note that, although the pathological rescue of TDP-43–related neurodegeneration and gliosis upon Nlk reduction was to near nontransgenic control levels, the observed improvement in survival in both TDP-43 transgenic mouse models was only around 20%, highlighting the need to test the therapeutic impact of Nlk reduction in animal models in which neurodegeneration, and not gastrointestinal complications (34) or other nonneurological problems that may arise from broad expression of the TDP-43 transgene outside of the central nervous system, primarily drives premature lethality. These future studies will likely require a comprehensive assessment of transgene expression distribution across central and peripheral tissues to determine the factors that contribute to premature lethality in the TDP-43 mouse models tested here.

Due to the precision afforded by complementary binding of an ASO to its target transcript of interest, ASOs have emerged as an invaluable tool in specifically altering levels and/or processing of a variety of proteins involved in neurodegenerative diseases, most of which have been the disease-causing protein itself (38, 51–54). While these approaches have proven to be extremely effective in preclinical genetic models of these disorders, the utility of such approaches is restricted to subsets of patients that carry specific, rare mutations in the gene of interest. In the case of ALS, the vast majority of patients have a sporadic form of the disease in which a genetic etiology is not identifiable; therefore, targeted reduction of a mutated protein is not possible. For this reason, alternative approaches that mitigate pathologies commonly observed across sALS and fALS, such as pathological TDP-43 aggregation (39) or premature polyadenylation and loss of stathmin-2, a key regulator of microtubule dynamics in motor neurons (55, 56), are highly desirable.

To our knowledge, the present study is one of the first to generate and validate the therapeutic benefit of ASOs targeting a disease modifier (and not the disease-causing gene itself) in rodent models of a neurodegenerative disease. Among these, we believe this study is the first to target a novel molecule that has not been implicated as a genetic risk factor for the disease being studied. Because no known neurological condition in humans has been associated with a reduction of Nlk, mice with partial genetic reduction of *Nlk* display no pathological (neurodegenerative or inflammatory), survival, or cognitive changes up to 52 weeks (57), and mice with postnatal whole-body conditional ablation of *Nlk* are grossly normal (57), we believe targeted reduction of *Nlk* or its downstream effectors to increase ALP function is a potentially generalizable therapeutic strategy for several neurodegenerative disorders, beyond just ALS. Further examination of the appropriate and effective time window for therapeutic intervention in specific disease contexts would be necessary prior to proceeding.

## Methods

### Animal husbandry.

Mice were maintained on a 12-hour light/12-hour dark cycle with standard mouse chow or high-fat breeder chow followed by DietGel food (ClearH_2_O) and water ad libitum. Two independent *Nlk* gene trap mouse lines (*Nlk^RRJ297/+^* and *Nlk^XN619/+^*) (7), referred to simply as *Nlk^+/–^*, were maintained on a pure C57BL/6J background, as previously described. We refer to compound heterozygous (*Nlk*^RRJ297/XN619^) mice as *Nlk* KO (*Nlk*^–/–^). Transgenic B6.Cg-Tg(Prnp-TARDBP*A315T)95Balo/J (*Prp-TDP^A315T/+^*) mice were obtained from The Jackson Laboratory (catalog 010700) and maintained on a pure C57BL/6J background. Transgenic B6;SJL-Tg(Thy1-TARDBP)4Singh/J (*Thy1-TDP^Tg/+^)* hemizygous mice were obtained from The Jackson Laboratory (catalog 012836) and maintained on a mixed B6;SJL background. For animal PCR genotyping, tail snips for DNA extraction were taken for *Thy1-TDP^Tg^* animals at P14 and between P18 and P20 for all other animals. Unless otherwise noted, both male and female mice were analyzed for behavior, survival, and pathological studies.

### N2a and NSC-34 cell lines.

N2a (ATCC) and NSC-34 (received from A. Horwich lab, Yale University) cells were cultured in DMEM (Gibco, Thermo Fisher Scientific), supplemented with 10% (v/v) FBS (Gibco, Thermo Fisher Scientific). Transient transfection was performed using Lipofectamine 2000 (Thermo Fisher) or polyethylenimine (PEI) (Polysciences) according to the manufacturer’s instructions. To prepare the PEI solution, 100 mg of PEI was dissolved in 100 ml of PBS while pH was monitored and maintained at 7, and the resulting solution was filtered; 1 ml aliquots of the PEI solution were stored at –20°C until use. Cells were analyzed 48 hours after transfection, unless otherwise noted. Cells were routinely tested for mycoplasma contamination by PCR as previously described (58) using primers for-GGCGAATGGGTGAGTAACACG and rev-CGGATAACGCTTGCGACCTATG. Any mycoplasma-positive samples were immediately discarded. For RFPGFP-LC3 experiments, ptfLC3 (Addgene, 21074) (16) was transfected as described above.

### N2a Nlk CRISPR cell-line generation.

For generation of N2a *Nlk* CRISPR cell lines, CRISPR single-guide RNA (sgRNA) sequences (sgRNA no. 1, TTGTTGCCCAGGGTTTAACA; sgRNA no. 2, CCCATCCCCGGCACCGGGTC; and sgRNA no. 3, ACACCACCTTCATCCGGGGT) targeting the mouse *Nlk* locus were cloned into pSpCas9n(BB)-2A-Puro (Addgene, 62987; PX462) or pSpCas9(BB)-2A-GFP (Addgene, 48138; PX458). Cells were transfected with gRNA and Cas9n-expressing plasmids using Lipofectamine 2000 or PEI, and successfully transfected cells were selected with 3 μg ml^–1^ puromycin for 2 days or collected by FACS. Single clones were generated by plating cells at a single-cell density in 96-well plates and then expanded to 24-well plates for genotyping by Western blotting.

### hiPSC NLK CRISPR cell-line generation.

For generation of hiPSC *NLK* CRISPR cell lines, a CRISPR guide RNA sequence (sgRNA no. 3, CTCAACACCATCTTCATCCG) targeting the human *NLK* locus was cloned into pSpCas9 (BB)-2A-GFP (Addgene, 481387). WT iPSCs were transfected with gRNA and Cas9-expressing plasmid using Amaxa Nucleofector 2b, and successfully transfected cells were collected by FACS, as previously described (58). Single clones were generated by plating cells at a single-cell density and then expanded to 24-well plates for genotyping by Sanger sequencing and Western blotting.

### hiPSC-derived motor neuron differentiation.

Human motor neurons were generated using a previously established protocol with minor modifications (59). Briefly, hiPSC colonies were split with Dispase (1 U/ml) (Stem Cell Technologies) and replated at a 1:6 ratio on Matrigel-coated plates in mTeSR1 medium. The next day (day 1), mTeSR1 medium was replaced with neural medium supplemented with 3 μM CHIR99021 (Tocris), 2 μM DMH1 (Tocris), and 2 μM SB431542 (Stemgent). The neural medium contained DMEM/F12 and Neurobasal medium mixed at a 1:1 ratio, 0.5× N2, 0.5× B27, 100 μM ascorbic acid (MilliporeSigma), 1× GlutaMAX (Life Technologies), and 1× penicillin/streptomycin (Life Technologies). The medium was replenished on day 3 and day 5. On day 7, neuroepithelial progenitors (NEPs) were split with dispase (1 U/ml) and replated at a 1:6 ratio on Matrigel-coated plates in neural medium supplemented with 1 μM CHIR99021 (Tocris), 2 μM DMH1 (Tocris), 2 μM SB431542 (Stemgent), 0.1 μM retinoic acid (RA) (Stemgent), and 0.5 μM purmorphamine (Stemgent). The medium was replenished on days 9, 11, and 13. On day 14, motor neuron progenitors (MEPs) were dissociated with Accutase (Stem Cell Technologies) and replated at a 1:6 ratio on Matrigel-coated plates in neural medium supplemented with 3 μM CHIR99021 (Tocris), 2 μM DMH1 (Tocris), 2 μM SB431542 (Stemgent), 0.1 μM RA (Stemgent), 0.5 μM purmorphamine (Stemgent), and 0.5 μM VPA (Stemgent). The medium was replenished on days 16, 18, and 20. On day 21, MEPs were dissociated with Accutase and replated at a 1:6 ratio on Matrigel-coated plates in neural medium supplemented with 0.5 μM RA (Stemgent) and 0.1 μM purmorphamine (Stemgent). The medium was replenished on days 23 and 25. From day 27, Compound E (Calbiochem) was supplemented to the above medium to facilitate motor neuron maturation, and media changes were performed every other day. Mature motor neurons were dissociated with Accutase and plated on Matrigel-coated 35 mm glass-bottom dished or Matrigel-coated coverslips for immunostaining.

### RNA extraction, sequencing, and analysis.

RNA was extracted using the QIAGEN RNeasy Mini Kit, and genomic DNA was removed with DNase I as recommended in the manufacturer’s instructions. The total RNA was sent to the Yale Center for Genome Analysis for processing. RNA integrity was measured using an Agilent Bioanalyzer 2100 (Agilent Technologies), and RIN values were assessed to ensure high RNA integrity before sequencing. Libraries were generated using oligo-dT purification of polyadenylated RNA, followed by reverse transcription into cDNA prior to being fragmented, blunt ended, and ligated to adaptors. The library was quantified before pooling and sequencing on an Illumina HiSeq 2000 using a 75 bp paired-end read strategy.

TopHat2 v2.1.0 was utilized to align reads to the mouse reference genome (GRCm38/mm10) before quantification and differential expression analysis with Cufflinks, version 2.2.1 (60–63). Cuffnorm was utilized for generating normalized expression values (62), and annotations with an FDR adjusted *P* value (*q* < 0.05) were considered significant. Enrichment analysis was carried out using Gene Set Enrichment Analysis (GSEA) (64, 65), ToppCluster (66), and Ingenuity Pathway Analysis (IPA) (QIAGEN Winter Release 2019) on all differentially regulated genes. Biological pathways and cellular components with enrichment scores greater than 1.3 (–log Bonferroni’s corrected *P* value for ToppCluster, and –log Benjamini-Hochberg corrected *P* value for IPA) were considered significant. The diseases function of Toppcluster, which links different gene expression profiles to those known for specific disorders, was utilized to investigate any diseases that have an overrepresented number of genes differentially regulated in the *Nlk-*KO cells. The Gene Ontology Consortium (GO) (67, 68) was utilized to generate gene lists for lysosome, autophagy, and stress granule–related genes. Enrichment score bar plots and heatmaps were plotted in R, version 3.3.3.

For real-time quantitative PCR (qPCR), cDNA was synthesized using oligo-dT primers and the iScript cDNA synthesis kit (Bio-Rad). qPCRs were run using TaqMan probes with iTaq Universal Probe Supermix on a C1000 Thermal Cycler (Bio-Rad) equipped with Bio-Rad CFX Manager software, version 3.1. The following TaqMan probes (Applied Biosystems) were used: *LAMP1* (Hs00931461_m1), *LAMP2* (Hs00174474_m1), *CTSA* (Hs00264902_m1), *CTSD* (Hs00157205_m1), *HPRT1* (Hs02800695_m1), *Nlk* (Mm00476435_m1), *TARDBP* (Hs00606522_m1), *Gfap* (Mm01253033_m1), *Aif1* (Mm00479862_g1), *Hprt* (Mm03024075_m1), and *Actb* (4352933E). Expression data were determined by normalizing target expression to housekeeping genes (*Hprt* and *Actb*) using Bio-Rad CFX Manager software, version 3.1, and then plotted using Prism 7 (GraphPad).

### Protein extraction and Western blot analysis.

Western blot was performed as described previously (8). For protein extraction from cells, cells were harvested and lysed in triple lysis buffer (0.5% NP-40, 0.5% Triton X-100, 0.1% SDS, 20 mM Tris-HCl [pH 8.0], 180 mM NaCl, 1 mM EDTA, and Roche cOmplete Protease Inhibitor Cocktail) for 15 minutes at 4°C, rotated, and then centrifuged for 10 minutes at 15,871*g* at 4°C.

For Nlk coimmunoprecipitation experiments, cell pellets were harvested in NP-40 lysis buffer (0.5% NP-40, 20 mM Tris [pH 8.0], 180 mM NaCl, 1 mM EDTA, and Roche cOmplete Protease Inhibitor Cocktail and PhosSTOP protease inhibitors) for 15 minutes at 4°C, rotated, and then centrifuged for 10 minutes at 15,871*g* at 4°C. A fraction of the resulting supernatant was set aside to be used as the input fraction. The remaining supernatant was incubated with anti-FLAG M2 magnetic beads overnight at 4°C. On the following day, the bound fraction was collected and analyzed by SDS-PAGE.

Protein extraction from mouse tissue for TDP-43 analysis was performed as previously described (3), with minor modifications. Briefly, frozen tissue was weighed and dounce homogenized in 5 ml/g LS buffer (10 mM Tris pH 7.5, 5 mM EDTA, 1 mM DTT, 10% sucrose, cOmplete EDTA-free protease inhibitors and PhosSTOP protease inhibitors) (Roche). Samples were ultracentrifuged at 25,000*g* for 30 minutes at 4°C, and the supernatant was collected as the low-salt fraction. The pellet was washed again with LS buffer and ultracentrifuged at 25,000*g* for 30 minutes at 4°C; the supernatant was discarded, and the pellet was then resuspended in HS buffer (LS buffer, 1% Triton X-100, 500 mM NaCl). The supernatant of this fraction was collected as the high-salt fraction. The pellet was resuspended in MF buffer (HS buffer, 30% sucrose) to remove myelin. Following ultracentrifugation at 180,000*g* for 30 minutes at 4°C, the pellet was resuspended in SK buffer (LS buffer, 1% sarkosyl, 500 mM NaCl) and solubilized at 22°C for 60 minutes using a ThermoMixer 5350 (Eppendorf) at 500 rpm. The solubilized mixture was then ultracentrifuged at 180,000*g* for 30 minutes at 22°C. The supernatant was collected as the sarkosyl fraction. The remaining pellet was resuspended in urea/SDS buffer (30 mM Tris-HCl, 7M urea, 2M thiourea, 2% SDS). This fraction was ultracentrifuged at 25,000*g* for 30 minutes at 22°C, and the supernatant was isolated as the urea fraction.

Total protein concentrations of isolated protein lysates were quantified using the BCA Protein Assay Kit (Thermo Fisher), and equal protein amounts were boiled at 95°C for 10 minutes, loaded, and analyzed by SDS-PAGE. Protein from gels was transferred onto PVDF or nitrocellulose membranes overnight at 4°C. The next day, membranes were washed 3 times and blocked by 5% nonfat dry milk in TBST for 1 hour at room temperature, followed by incubation with primary antibody in 1% nonfat dry milk in TBST at 4°C overnight. Membranes were washed with TBST 3 times and incubated with sheep anti-mouse or donkey anti-rabbit IgG conjugated with horseradish peroxidase (HRP) (1:4,000, GE Healthcare) for 1 hour at room temperature. Membranes were developed using Western Lightning Plus-ECL Reagent (PerkinElmer) and visualized using a SRX-101A tabletop X-ray film processor (Konica Minolta) or KwikQuant Imager (Kindle Biosciences).

The following primary antibodies were used: rabbit anti-IFITM3 (ProteinTech; 11714-1-AP; 1:2,000), rabbit anti–annexin VI (Abcam; ab31026; 1:1,000), mouse anti-vinculin (Sigma-Aldrich; V9264; 1:10,000), rabbit anti-lamp2a (Abcam; ab18528; 1:1,000), rabbit anti-CatD (Abcam; ab75852; 1:2,000), mouse anti-Gapdh (Sigma-Aldrich; G8795; 1:10,000), rabbit anti-LC3B (Abcam; ab51520; 1:3,000), mouse anti-p62 (Novus; H00008878-M01; 1:10,000), rabbit anti-NLK (Abcam; ab26050; 1:1,000), rabbit anti-TFEB (MyBioSource; MBS004492; 1:500), mouse anti-TFEB (MyBioSource; MBS120432; 1:500), rabbit anti–phospho-TFEB (Ser122) (Cell Signaling; 86843; 1:500), rabbit anti–phospho-TFEB (Ser142) (Millipore; ABE1971-I; 1:500), rabbit anti-GFP (Abcam; ab290; 1:1,000), mouse anti-ubiquitin (Santa Cruz Biotechnology Inc.; sc-53509; 1:200), mouse anti-tubulin (Sigma-Aldrich; T6557; 1:10,000), rabbit anti-histone H3 (Millipore; 05-928; 1:5,000), rabbit anti-TARDBP (Novus; NB110-55376; 1:1,000), rabbit anti-TARDBP (Proteintech; 10782-2-AP; 1:1,000), mouse anti-Flag (Sigma-Aldrich; F3165; 1:10,000), rabbit anti-HA (abcam; ab9110; 1:5000); rabbit anti–4E-BP1 (Cell Signaling Technology; 9644, 1:1,000), rabbit anti–phospho-4E-BP1 (Cell Signaling Technology; 2855; 1:1,000), rabbit anti-ULK1 (Cell Signaling Technology; 8054; 1:1,000), rabbit anti–phospho-ULK1 (Cell Signaling Technology; 14202; 1:1,000), rabbit anti-S6 (Cell Signaling Technology; 2217; 1:1,000), and rabbit anti–phospho-S6 (Cell Signaling Technology; 2215; 1:1,000).

### Cytosolic and nuclear fractionation.

N2a cells transfected with indicated plasmids were lysed in fractionation buffer containing 0.5% Triton X-100, 50 mM Tris-HCl, 137.5 mM NaCl, 10% glycerol, and 5 mM EDTA and incubated on ice for 20 minutes, followed by centrifugation for 3 minutes at 1,000*g* at 4°C. The supernatant was then transferred to a new Eppendorf tube and centrifuged again for 5 minutes at 21,130*g* at 4°C. This supernatant was used as a cytosolic fraction. The pellet from the first centrifugation was washed with fractionation buffer twice and then suspended in 0.5% SDS in 100 mM Tris-HCl. These samples were sonicated and centrifuged for 10 minutes at 15,871*g* at 4°C. The supernatant after this centrifugation was used as a nuclear fraction. Cytosolic and nuclear fractions were quantified using the BCA Protein Assay Kit and analyzed by SDS-PAGE and Western blotting, as described above.

### Animal tissue collection and cryosectioning.

Mice were euthanized using isoflurane and perfused with ice-cold PBS for 3 minutes to remove blood. Spinal cords were then extracted by hydraulic extrusion, as described previously (69). Briefly, mice were decapitated and skin along the mouse back was removed. The spinal column was cut transversely at the pelvic bone. A syringe loaded with cold PBS was inserted into the caudal spinal column, and the spinal cord was extruded using hydraulic force. The thoracic and sacral portions of the spinal cord were immediately flash frozen using dry ice with isopropanol and used for protein and RNA extraction, respectively. Fresh lumbar cord tissue was post-fixed in cold 4% paraformaldehyde (PFA) for 48 hours and cryopreserved by immersion in a 20% to 30% sucrose gradient, followed by freezing in OCT compound (VWR). Fresh brains were dissected and cut into sagittal halves. The right brain was post-fixed for 48 hours and cryopreserved using a 20% to 30% sucrose gradient, followed by freezing in OCT compound. The left brain was macrodissected, and individual tissues were flash frozen using dry ice with isopropanol, then stored at –80°C until further processing.

Embedded and frozen lumbar spinal cords were serially sectioned transversely as 30 μm sections directly onto SuperFrost Plus slides (Thermo Fisher) using a Cryostat CM1850 (Leica). Slides with spinal cord sections were stored at –80°C until further use. Slides with spinal cord sections from comparable segments were identified using gross anatomy under a light microscope and selected for further analysis. Embedded and frozen brains were sectioned using a Cryostat CM1850 as 30 μm free-floating sagittal sections in PBS. Floating sections were stored in PBS at 4°C until immunostaining was performed.

### Immunostaining and microscopy.

Cultured cells were fixed with 4% PFA in PBS for 10 minutes at room temperature followed by washing 3 times with PBS and incubation with 5% normal goat serum (NGS) for 1 hour at room temperature. Immunocytochemical staining was performed by overnight incubation with primary antibodies at 4°C. On the next day, primary antibody was removed and coverslips were washed with PBS 3 times and incubated with Alexa Fluor 488–, Alexa Fluor 555–, Alexa Fluor 568–, Alexa Fluor 594–, or Alexa Fluor 633–conjugated secondary antibodies (1:500, Invitrogen) for 2 hours at room temperature, protected from light. Cells were counterstained with TO-PRO-3 (1:5,000, Invitrogen) together with secondary antibodies when necessary. Immunostained cells were mounted onto slides using Vectashield Mounting Reagent (Vector Laboratories). For immunohistological staining of mouse tissue, sections were washed 3 times with 0.25% Triton X-100 (American Bioanalytical) in PBS (PBS-X) and blocked with 5% NGS in PBS-X for 1 hour, followed by overnight incubation with primary antibodies at 4°C. On the next day, sections were washed 3 times with PBS-X and incubated with conjugated secondary antibodies (1:500, Invitrogen) for 2 hours at room temperature, protected from light. Sections were then washed 3 times with PBS-X, once with PBS, and mounted using Vectashield Mounting Reagent with DAPI (Vector Laboratories).

For LysoTracker imaging, N2a cells were incubated with 100 nM LysoTracker Red DND-99 (Invitrogen) in a 5% CO_2_ incubator at 37°C for 55 minutes followed by 3 washes with HBSS. Live imaging was then performed using an UltraVIEW VoX (PerkinElmer) inverted spinning disc confocal microscope equipped with a ×60 CFI Plan APO VC, NA 1.4, oil objective, and Volocity acquisition software, version 6.0, Improvision) (Figure 1) or a Zeiss LSM880 confocal microscope (Supplemental Figure 3). Cells were maintained in Live Cell Imaging Solution (Invitrogen) at 37°C in 5% CO_2_ during image acquisition, and microscope acquisition parameters for all images were identical. Lysosome number per cell was blindly quantified and normalized by cell area.

For DQ BSA assays, N2a cells were incubated with 10 μg ml^-1^ DQ Red BSA (Thermo Fisher) at 37°C for 2 hours and washed with PBS twice. Live-cell imaging was performed within 1 hour after staining using a Zeiss LSM880 confocal microscope by maintaining cells in Live Cell Imaging Solution (Invitrogen) at 37°C in 5% CO_2_ during image acquisition. Microscope acquisition parameters for all images were identical. Lysosome number per cell was blindly quantified.

Unless otherwise noted, imaging was performed using a Zeiss LSM880 confocal microscope or Zeiss LSM710 confocal microscope and processed with ImageJ software (NIH). Number of NeuN-positive layer V cortical neurons was quantified as previously described (39), with minor modifications. Briefly, following acquisition and stitching of a 3 × 3 tiled array of ×20 images, stitched images were deidentified and randomized. The polygon selection tool of Fiji (70) was used to outline layer V, and the area of the selection was measured using the measure tool and recorded. Then the area outside of the selection was removed using the clear selection tool and a threshold was applied using the otsu algorithm. Overlapping nuclei were separated using the watershed tool, and nuclei larger than 50 μm^2^ with a circularity between 0.2 and 1.0 were counted using the analyze particles tool. The number of nuclei was divided by the area of the polygon selection to determine density of nuclei. For all histological analyses, at least 3 animals were examined, with at least three to nine 30 μm sections from comparable brain/spinal cord regions quantified per animal. For Lamp1 quantification in spinal cords, only cells with soma size greater than 200 μm^2^ were counted. The experimenter was blind to animal genotype and ASO treatment during image acquisition and quantification.

The following primary antibodies used were as follows: rat anti-lamp2 (Abcam; ab13524; 1:100), mouse anti–cathepsin D (Abcam; ab6313; 1:200), goat anti-ChAT (Millipore; AB144P; 1:500), rabbit anti-Lamp1 (Abcam; ab24170; 1:400), chicken anti-GFAP (Abcam; ab4674; 1:2,000), mouse anti-NeuN (Millipore; MAB377; 1:200), mouse anti-SMI32 (Covance; SMI-32P; 1:1,000), mouse anti-Flag (Sigma-Aldrich; F3165; 1:2000), and chicken anti-GFP (Abcam; ab13970; 1:3,000).

### Luciferase reporter assay.

Cells were transfected with 4XCLEAR-Luciferase reporter construct (Addgene, 66800) (13) and internal control pRL-TK vector (Promega) plasmids using Lipofectamine 2000. Transfected cells were cultured in DMEM supplemented with 10% FBS for 2 days. Cells were then collected, lysed, and subjected to dual luciferase reporter assay using the Dual-Luciferase Reporter Assay System (Promega) and the GloMax 2020 Luminometer (Promega) according to the manufacturer’s instructions. Luciferase activity was normalized by dividing the *firefly* luciferase activity from the 4XCLEAR-Luciferase construct by the *Renilla* luciferase activity from the pRL-TK vector. All experiments were repeated in triplicate and performed at least 3 times.

### ASO generation and administration.

Synthesis and purification of control ASO (CCTATAGGACTATCCAGGAA) or *Nlk*-targeting ASO (GTCACAGTACAGCCTGGATC) were performed as previously described (71). The MOE-gapmer ASOs are 20 nucleotides in length, wherein the central gap segment comprises ten 2′-deoxyribonucleotides that are flanked on the 5′ and 3′ wings by five 2′ MOE modified nucleotides. Internucleotide linkages were phosphorothiorate interspersed with phosphodiester, and all cytosine residues were 5′ methylcytosines. ASOs were delivered i.c.v. through the right hemisphere (2 mm anterior to lambdoid suture, 1 mm lateral from sagittal suture, 2 mm deep) of hypothermia-induced anesthetized mice at P1 using the 10 ml Model 1701 RN Syringe with a 33-gauge, 0.375 inch, custom point style 4, 45 angle needle (Hamilton).

### Animal behavioral assessment.

Grip strength was assessed in P84 *Prp-TDP^A315T^* animals and littermate controls using a Chatillon grip strength meter (Columbus Instruments). Six measurements were taken for forelimb or forelimb with hind limb grip strength per animal. The maximum and minimum force measurements were discarded, the remaining 4 were averaged, and the average was recorded for each animal. The experimenter was blind to animal genotype and ASO treatment.

Motor behavior of *Thy1-TDP^Tg^* animals and littermate controls was examined at P14, P16, P18, P20, and P22, as previously described (39, 45), with minor modifications. Briefly, animals were assessed for gait impairment, tremor, kyphosis, and hind limb clasping. To assess gait impairment, tremor, and kyphosis, animals were placed into a new cage on a flat, but textured plastic surface. For gait impairment, a score between 0 and 4 was assigned based on the following criteria: normal movement, 0; grossly normal movement with a mild tremor or limp, 1; severe tremor, limp, or lowered pelvis during movement, 2; difficulty moving forward with frequent falls, but is able to right itself within 30 seconds of falling, 3; and unable to right itself in 3 consecutive 30-second trials, 4, also considered the humane end point for euthanasia. For tremor, a score between 0 and 3 was assigned based on the following criteria: no tremor, 0; mild tremor while moving, 1; severe jerking tremor while moving, 2; and severe tremor at rest and during movement, 3. For determining kyphosis score, a score between 0 and 3 was assigned based on the following criteria: no observable kyphosis, 0; mild kyphosis at rest, but able to straighten spine completely during movement, 1; mild kyphosis at rest and unable to straighten spine completely during movement, 2; and severe kyphosis at rest that is maintained during movement, 3.

To assess hind limb clasping, animals were suspended by the base of their tail and the hind limb positioning was observed for 10 seconds. A score between 0 and 3 was assigned based on the following criteria: hind limbs extended outwards for 50% or more of trial, 0; 1 hind limb pointing inward toward the abdomen for 50% or more of trial, 1; both hind limbs partially pointed toward the abdomen for 50% or more of the trial, 2; and both hind limbs completely clasped inwards together against the abdomen for 50% or more of the trial, 3. Composite motor behavior scores were calculated by summing gait impairment, kyphosis, tremor, and hind limb clasping scores (maximum score of 13) for each animal. For animals that reached their humane end point prior to P22 measurements, the scores for gait impairment, tremor, and hind limb clasping at time of end point were also reported as scores for the remaining recordings for those animals. For animals sacrificed at P19 for pathology studies, no scores were reported for P20 and P22 time points. The experimenter was blind to animal genotype and ASO treatment.

### Statistics.

Unless otherwise noted, all data were analyzed using 2-tailed, unpaired Student’s *t* test (2 experimental groups) or 1-way ANOVA with multiple comparisons (more than 2 experimental groups) to determine statistical significance between samples using Prism 7. *P* < 0.05 was considered to be statistically significant. Graphs were plotted using Prism 7, and heatmaps and volcano plots were plotted using R version 3.3.3.

### Study approval.

All animal procedures were performed in accordance with the NIH *Guide for the Care and Use of Laboratory Animals* (National Academies Press, 2011) and approved by the Yale University Institutional Animal Care and Use Committee (protocol 2021-11342).

### Data availability.

RNA data FASTQ files and processed normalized output were deposited in the NCBI’s Gene Expression Omnibus database (GEO GSE235312). Values for all data points found in graphs can be found in the supporting data values file. All raw, uncropped Western blots are available as supplemental material. Additional details regarding data and protocols that support the findings of this study are available from the corresponding author upon request. The ASOs used in this study are produced by Ionis Pharmaceuticals, a for-profit company.

## Author contributions

LT, YJ, HK, and JL conceived and designed this study. YJ, HK, and BS generated Nlk-KO N2a cells and performed in vitro Tfeb, TDP-43, and autophagy experiments. LT, SS, C Lee, and JP performed N2a LysoTracker microscopy, and LT, SS, and JY quantified images. SS performed N2a immunostaining and DQ BSA assays. LN performed N2a proteasome activity assays. PJL performed bioinformatic analysis of RNA-Seq data and generated heatmaps and volcano plots. LT and C Lee performed coimmunoprecipitation assays. LT and JL performed mouse survival studies. LT and YX generated mutant NLK hiPSC lines and performed hiPSC-derived motor neuron experiments. LT, with help from HJ, performed *Nlk* genetic reduction mouse experiments. AS, FR, and PJN generated ASOs and advised on experimental design for ASO experiments. LT performed mouse ASO experiments, with help from C Lee, PJL, KL, JY, HR, C Liao, and SMT for mouse behavior, dissections, immunostaining, protein, and RNA extraction. LT, PJL, and JL maintained the mouse colony and genotyped animals. LT, YJ, and JL wrote the manuscript. Author order was determined based on overall contribution to project conception, data collection and analysis, and manuscript preparation.

## Supplementary Material

Supplemental data

Supplemental table 1

Supplemental table 2

Supplemental video 1

Supporting data values

## Version 1 06/29/2023

In-Press Preview

## Version 2 08/15/2023

Electronic publication
