# Supplementary material for: Reduction of nemo-like kinase increases lysosome biogenesis and ameliorates TDP-43–related neurodegeneration
Source: J Clin Invest. 2023 Aug 15;133(16):e138207. doi: 10.1172/JCI138207 (PMC10425213; doi:10.1172/JCI138207)

Supplementary Materials

Supplemental Figure 1. Generation of isogenic *Nlk* N2a cell lines using CRISPR/Cas9.

Supplemental Figure 2. RNA-seq of *Nlk* KO cells reveals transcriptional changes associated with the autophagy-lysosome pathway.

Supplemental Figure 3. Nlk inhibits protein clearance through the autophagy-lysosome pathway.

Supplemental Figure 4. Generation of *NLK*-deficient human iPSC-derived motor neurons.

Supplemental Figure 5. Nlk has no effect on mTOR activity.

Supplemental Figure 6. Nlk does not physically interact with TDP-43.

Supplemental Figure 7. Reduction of *Nlk* improves survival and delays onset of disease in *Prp-TDP*$^{A315T/+}$ mice.

Supplemental Figure 8. Pharmacological reduction of *Nlk* does not induce gliosis in vivo.

Supplemental Figure 9. ASOs targeting *Nlk* ameliorate motor behavior deficits in *Thy1-TDP*$^{Tg/Tg}$ mice.

Supplemental Figure 10. ASOs targeting *Nlk* ameliorate motor behavior deficits in high fat/gel diet-fed *Thy1-TDP*$^{Tg/Tg}$ mice.

Supplemental Figure 11. ASOs targeting *Nlk* rescue pathology deficits in two TDP-43 mouse models.

Supplemental Table 1. Differential gene expression from RNA-seq of *Nlk* KO N2a cells.

Supplemental Table 2. List of differentially expressed genes related to lysosomal function, autophagy, and stress granules from RNA-seq of *Nlk* KO N2a cells.

Supplemental Video 1. ASOs targeting *Nlk* partially rescue motor impairment in *Thy1-TDP*$^{Tg/Tg}$ mice.

A

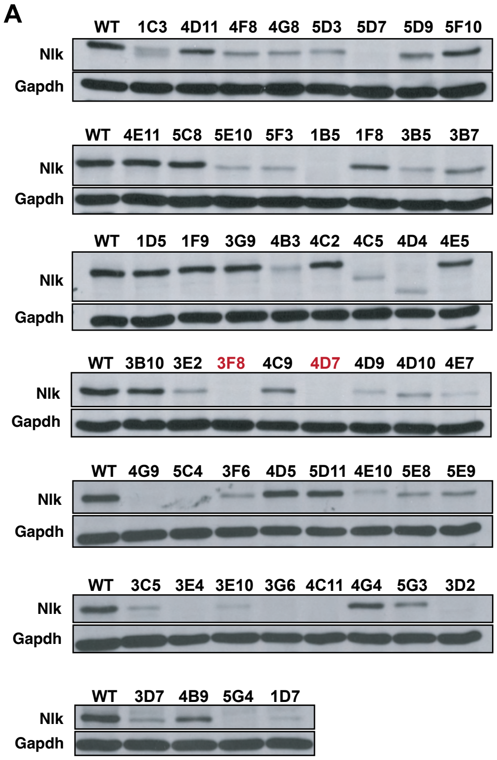

B

CRISPR Result	Clone ID
Nlk WT	4D11, 5F10, 4E11, 5C8, 1D5, 1F9, 3G9, 4C2, 4E5, 3B10, 4D5, 5D11, 4G4
Nlk KD	1C3, 4F8, 4G8, 5D3, 5D9, 5E10, 5F3, 1F8, 3B5, 3B7, 4B3, 3E2, 4C9, 4D9, 4D10, 4E7, 3F6, 4E10, 5E8, 5E9, 3C5, 3E10, 5G3, 3D7, 4B9, 1D7
Δ *Nlk*	4C5, 4D4
Nlk KO	5D7, 1B5, **3F8**, **4D7**, 4G9, 5C4, 3E4, 3G6, 4C11, 3D2, 5G4

Fig. S1_Tejwani et al.

Supplemental Figure 1. Generation of isogenic *Nlk* N2a cell lines using CRISPR/Cas9.
(**A**) Western blots of protein lysates detecting the degree of mutagenesis in individual clones isolated following dual guide RNA targeting of Cas9 nickase to *Nlk*.
(**B**) Summary of genotypes of *Nlk* CRISPR clones determined remaining levels of Nlk protein. WT= wild-type, KD= knock-down, KO= knock-out. Δ= partial deletion. *Nlk* KO clones 3F8 and 4D7 were selected for further experiments.

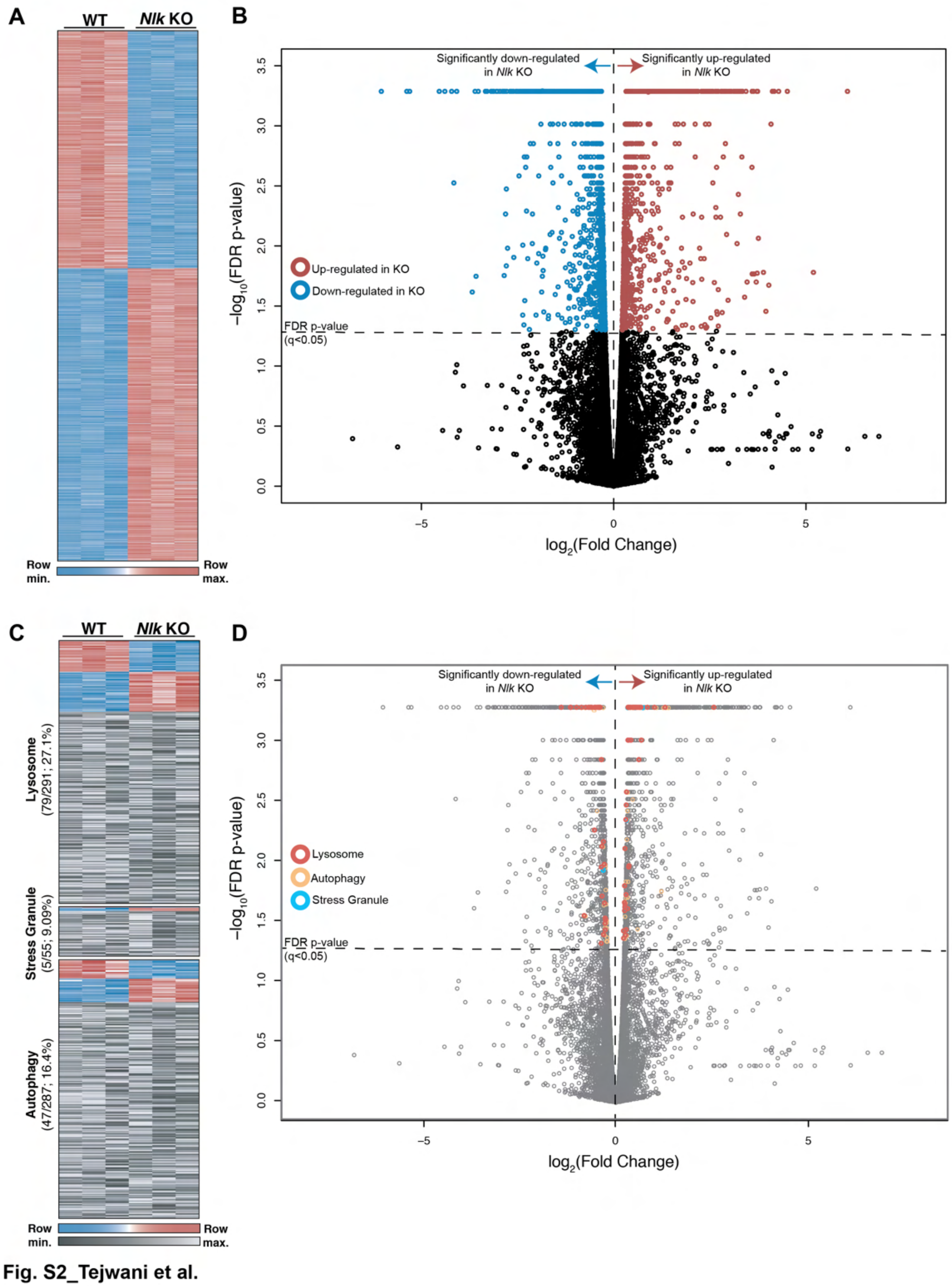

Supplemental Figure 2. RNA-seq of *Nlk* KO cells reveals transcriptional changes associated with the autophagy-lysosome pathway.

(**A**) Heatmap of all significantly altered genes from RNA-seq analysis of *Nlk* KO N2a cells.

(**B**) Volcano plot of RNA-seq results displaying all significantly up-regulated (red) and down-regulated (blue) genes.

(**C**) Individual heatmaps displaying all significantly altered (blue/red) and not significantly altered (gray) annotated lysosome, autophagy, stress granule-associated genes with detectable expression.

(**D**) Volcano plot of RNA-seq results with differentially-expressed lysosome, autophagy, and stress granule-associated genes.

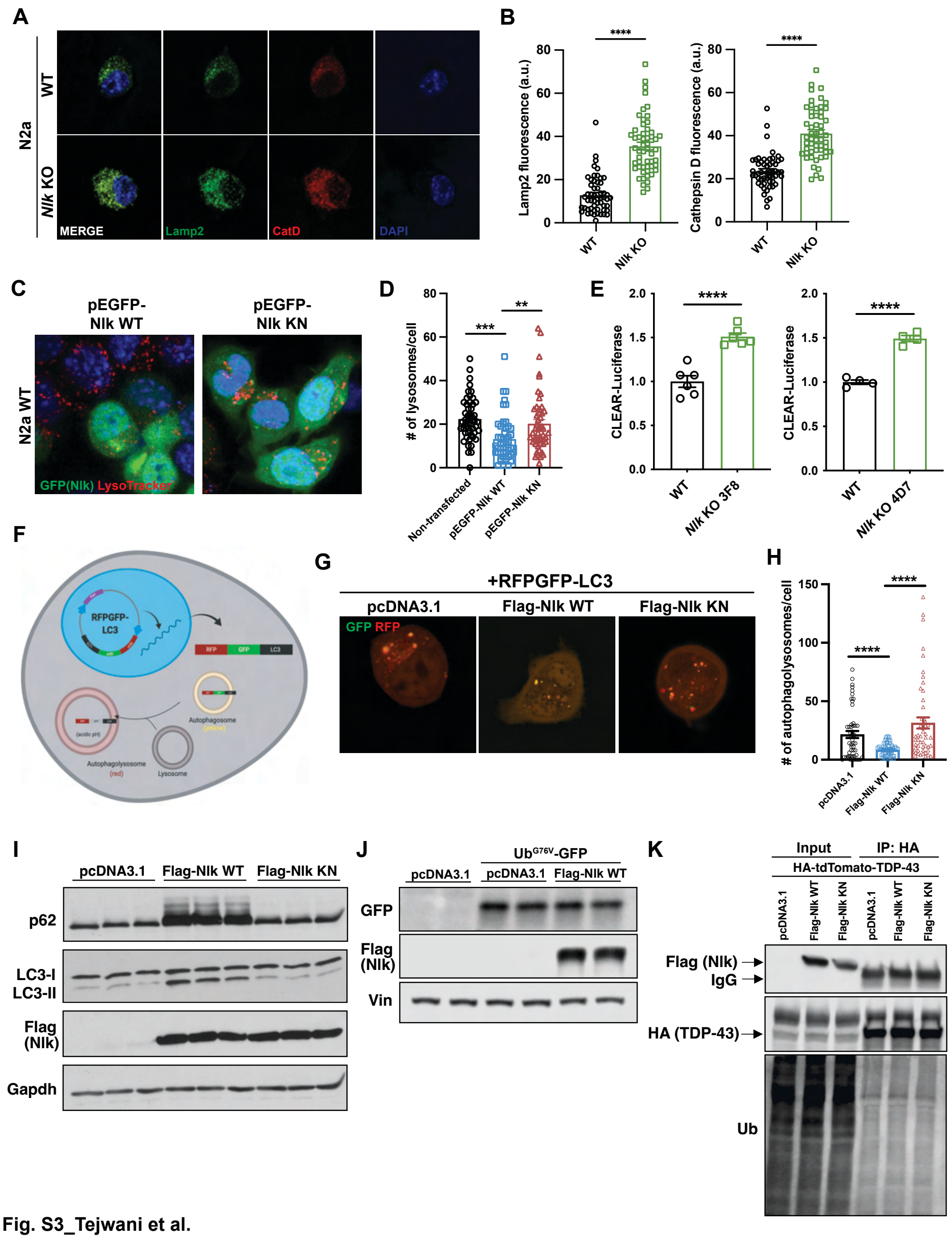

Fig. S3_Tejwani et al.

Supplemental Figure 3. Nlk inhibits protein clearance through the autophagy-lysosome pathway.
(**A** and **B**) Representative immunofluorescence images of Lamp2^{+} and CatD^{+} lysosomes in WT and *Nlk* KO N2a cells (**A**), quantified in (**B**).
(**C** and **D**) LysoTracker imaging of N2a WT cells transfected with EGFP-tagged WT or KN Nlk. Cells successfully transfected with WT Nlk (green) contained fewer LysoTracker-positive vesicles compared to non-transfected cells or cells transfected with kinase-inactive Nlk (Nlk KN), quantified in (**D**).
(**E**) CLEAR-Luciferase assay in two independent *Nlk* KO N2a clones (pooled in Figure 1K).
(**F**) Schematic detailing the tandem fluorescent-tagged RFPGFP-LC3. Transfected cells express the RFPGFP-LC3 fusion protein, which is trafficked into autophagosomes, where red and green co-localized fluorescence will be observed. Upon fusion with a lysosome, the green fluorescence is quenched by the pH of the lysosome and red-labeled vesicles can be detected.

(**G** and **H**) Representative images of N2a WT cells co-transfected with RFPGFP-LC3 and Flag-Nlk WT or Flag-Nlk KN (**G**). Overexpression of WT Nlk reduced the number of autophagolysosomes (red) (**H**).
(**I**) Western blots showed accumulation of p62 and LC3-II due to the failure of lysosome-dependent degradation in cells with WT Nlk overexpression.
(**J**) Western blots of N2a cells transfected with Ub^{G76V}-GFP 26S proteasome activity reporter. Overall proteasome activity was not significantly changed with Nlk overexpression.
(**K**) Co-immunoprecipitation showing that Nlk overexpression did not promote ubiquitination of TDP-43.
Two-tailed t-tests were performed and mean±SEM are displayed. **$p<0.01$, ***$p<0.001$, ****$p<0.0001$.

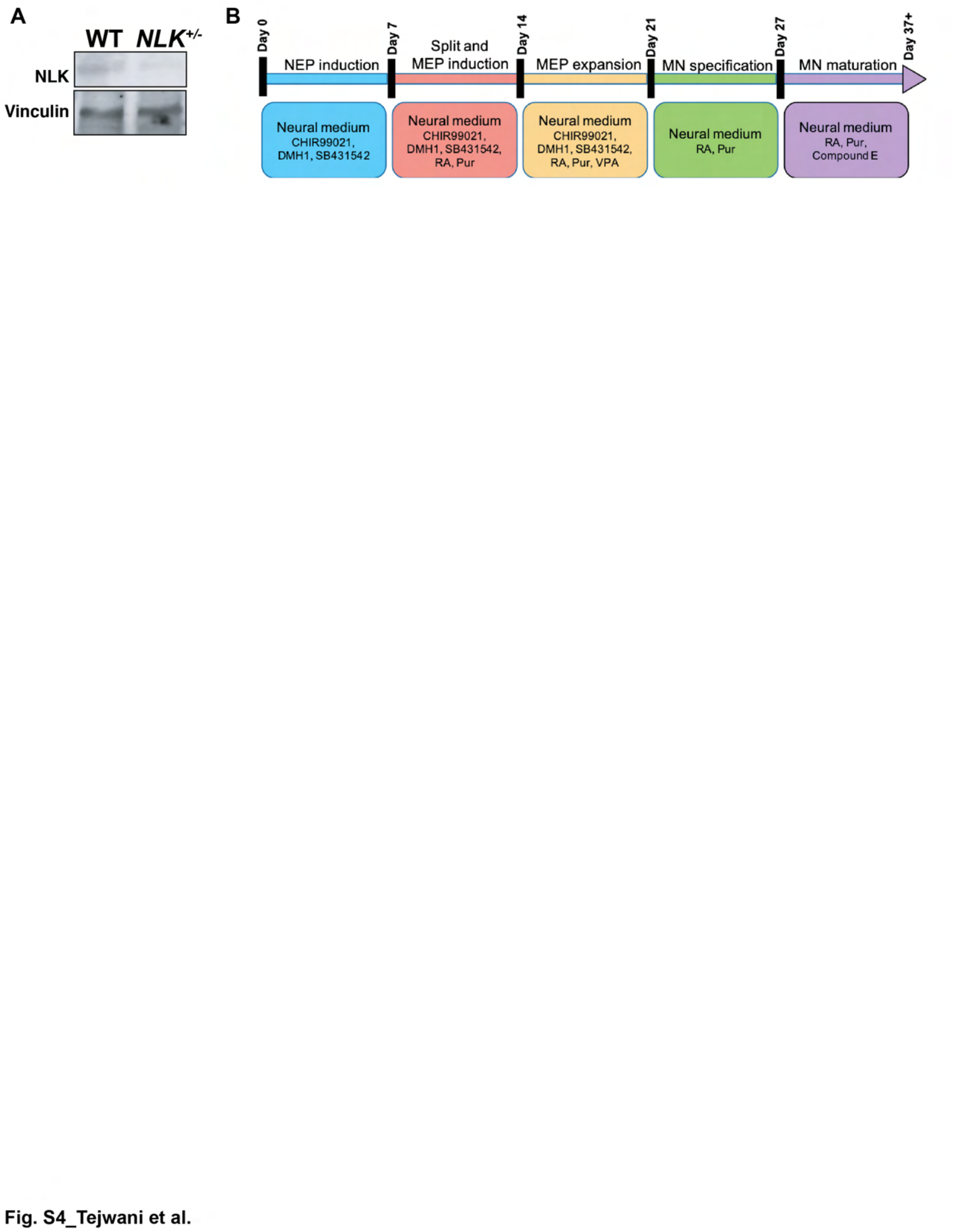

Supplemental Figure 4. Generation of *NLK*-deficient human iPSC-derived motor neurons.
(**A**) Western blots of day 38 WT and isogenic *NLK* heterozygous clones differentiated into motor neurons.
(**B**) Schematic detailing the protocol for generation of spinal motor neurons from iPSCs.

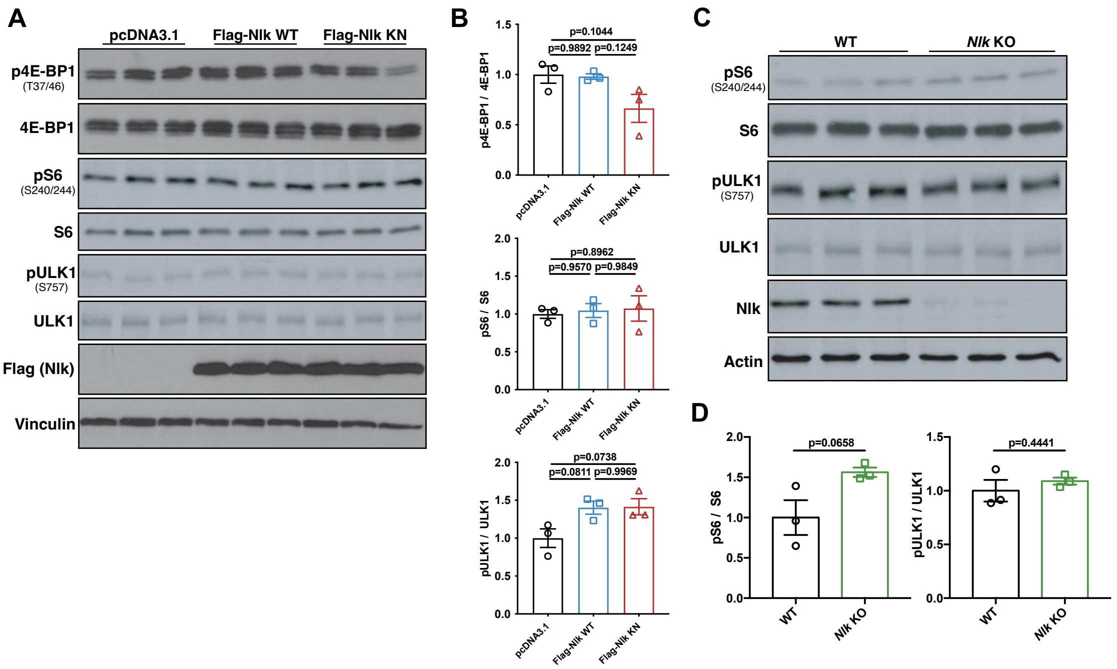

Fig. S5_Tejwani et al.

Supplemental Figure 5. Nlk has no effect on mTOR activity.
(**A** and **B**) Western blots of N2a whole-cell protein lysates showed WT Nlk or KN Nlk overexpression did not affect p4E-BP1/4E-BP1, pS6/S6, or pULK1/ULK1 ratios, suggesting mTOR independence (**A**), quantified in (**B**).
(**C** and **D**) Western blots of N2a whole-cell protein lysates showed *Nlk* KO did not affect pS6/S6 or pULK1/ULK1 ratios (**C**), quantified in (**D**).

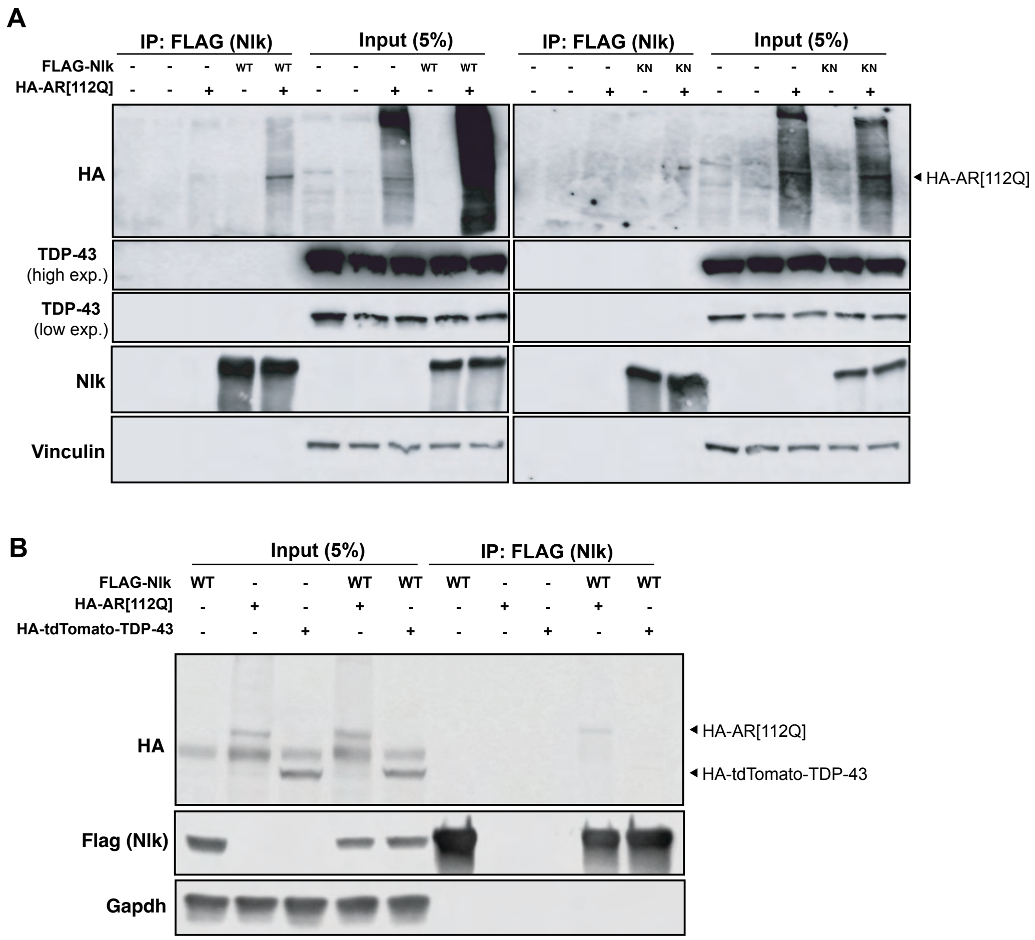

Fig. S6_Tejwani et al.

Supplemental Figure 6. Nlk does not physically interact with TDP-43.
(**A** and **B**) Co-immunoprecipitation showing that WT Nlk and KN Nlk interact with AR but not with endogenous TDP-43 (**A**) or overexpressed TDP-43 (**B**). Interaction of Nlk with AR serves as a positive control (see ref. 8).

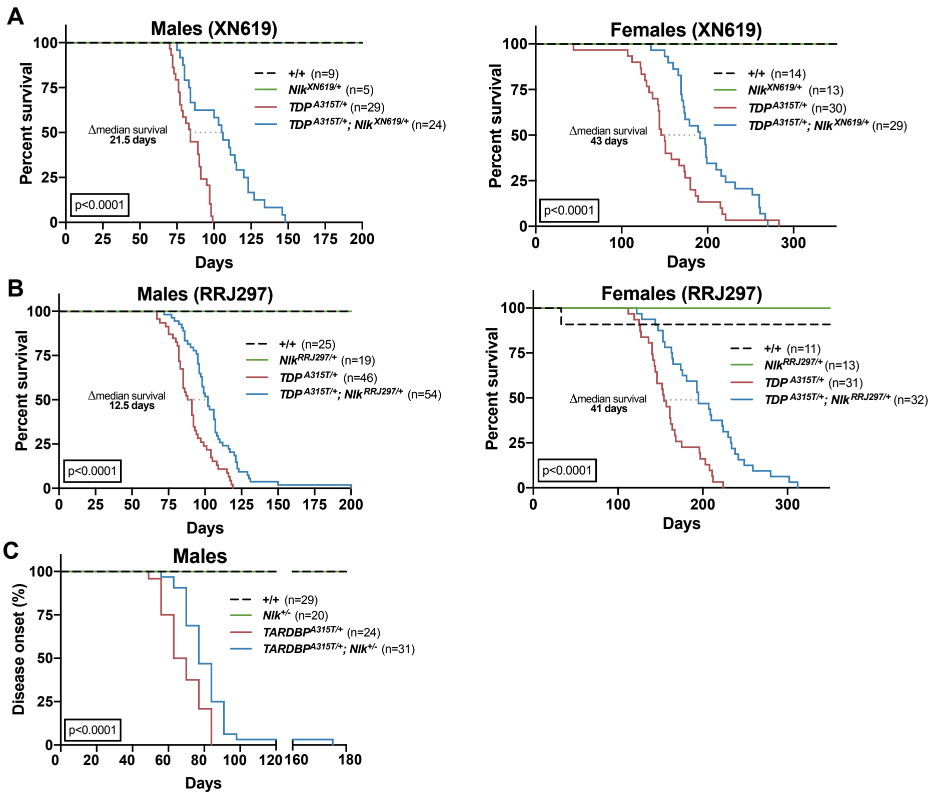

Fig. S7_Tejwani et al.

Supplemental Figure 7. Reduction of *Nlk* improves survival and delays onset of disease in *Prp-TDP*$^{A315T/+}$ mice.

(**A**) Kaplan-Meier survival curves showing 50% reduction of *Nlk* by crossing *Prp-TDP*$^{A315T/+}$ mice with *Nlk*$^{XN619/+}$ improved male and female animal survival. Curves were compared by long-rank test.

(**B**) Kaplan-Meier survival curves showing 50% reduction of *Nlk* by crossing *Prp-TDP*$^{A315T/+}$ mice with *Nlk*$^{RRJ297/+}$ improved male and female animal survival. Curves were compared by log-rank test.

(**C**) Kaplan-Meier curves showing 50% reduction of *Nlk* by crossing *Prp-TDP*$^{A315T/+}$ mice with *Nlk*$^{+/-}$ significantly slowed time to disease onset (defined by the earliest time point in which weight gain was no longer observed) in male mice.

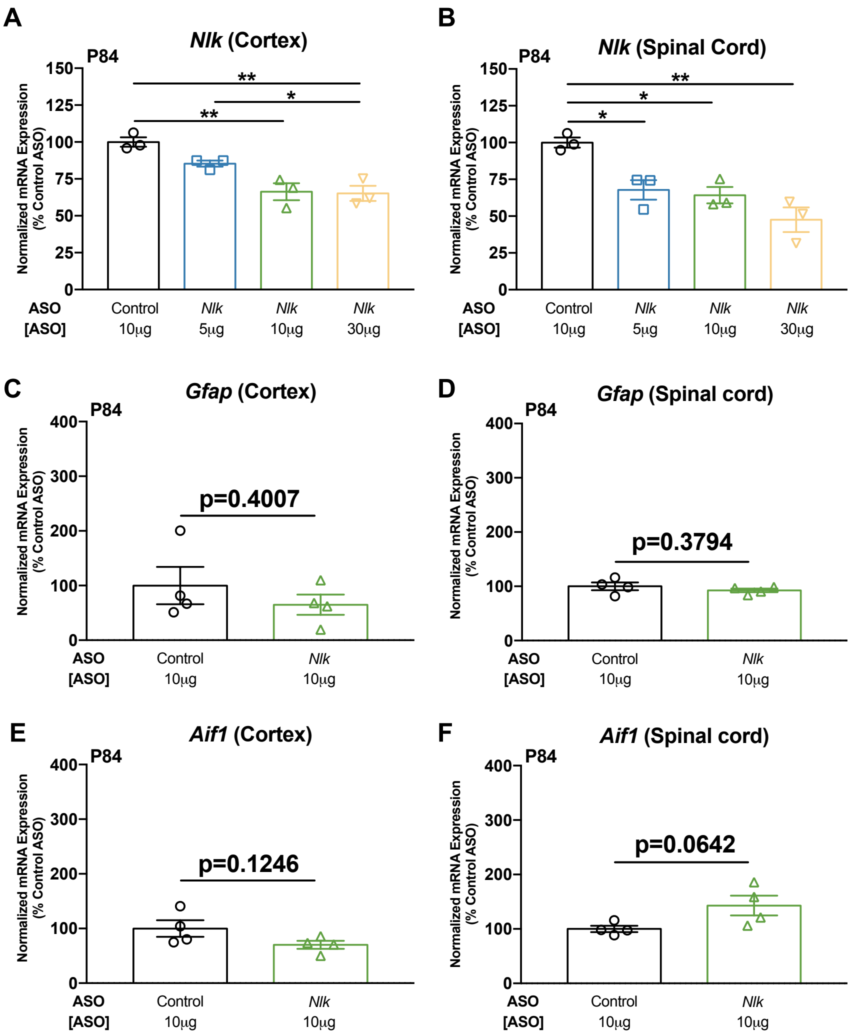

Fig. S8_Tejwani et al.

Supplemental Figure 8. Reduction of *Nlk* does not induce gliosis in vivo.

(**A-F**) P1 delivery of *Nlk* ASOs reduced *Nlk* mRNA levels in a dose-dependent manner (**A** and **B**) without inducing an overt astrogliosis (**C** and **D**) or microgliosis (**E** and **F**) in the cortex and spinal cord of P84 animals.

One-way ANOVA analyses were performed to compare effects of varying ASO dosage. Two-tailed t-tests were performed to compare conditions unless otherwise noted and mean±SEM are displayed. *p<0.05, **p<0.01.

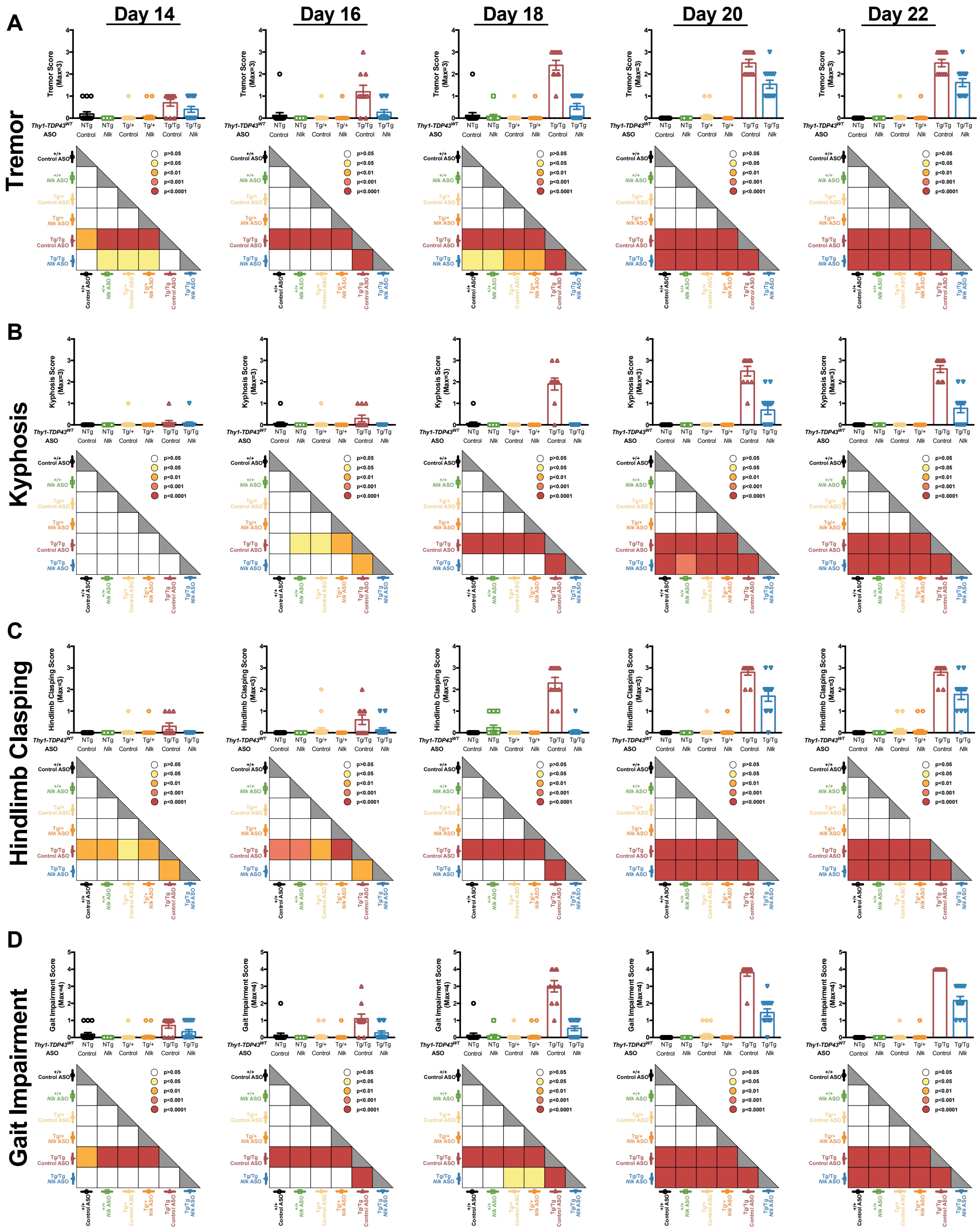

Fig. S9_Tejwani et al.

Supplemental Figure 9. ASOs targeting *Nlk* ameliorate motor behavior deficits in *Thy1-TDP*$^{Tg/Tg}$ mice. (**A-D**), Bar plots showing tremor (**A**), kyphosis (**B**), hindlimb clasping (**C**), and gait impairment (**D**) scores for ASO-injected animals on days 14, 16, 18, 20, and 22. Higher score numbers indicated increasing severity (see Methods). Results of statistical analyses are reported in each triangle below the corresponding bar graph.

One-way ANOVA analyses were performed to compare each genotype/condition on each individual day and reported in the statistics triangle below each bar plot.

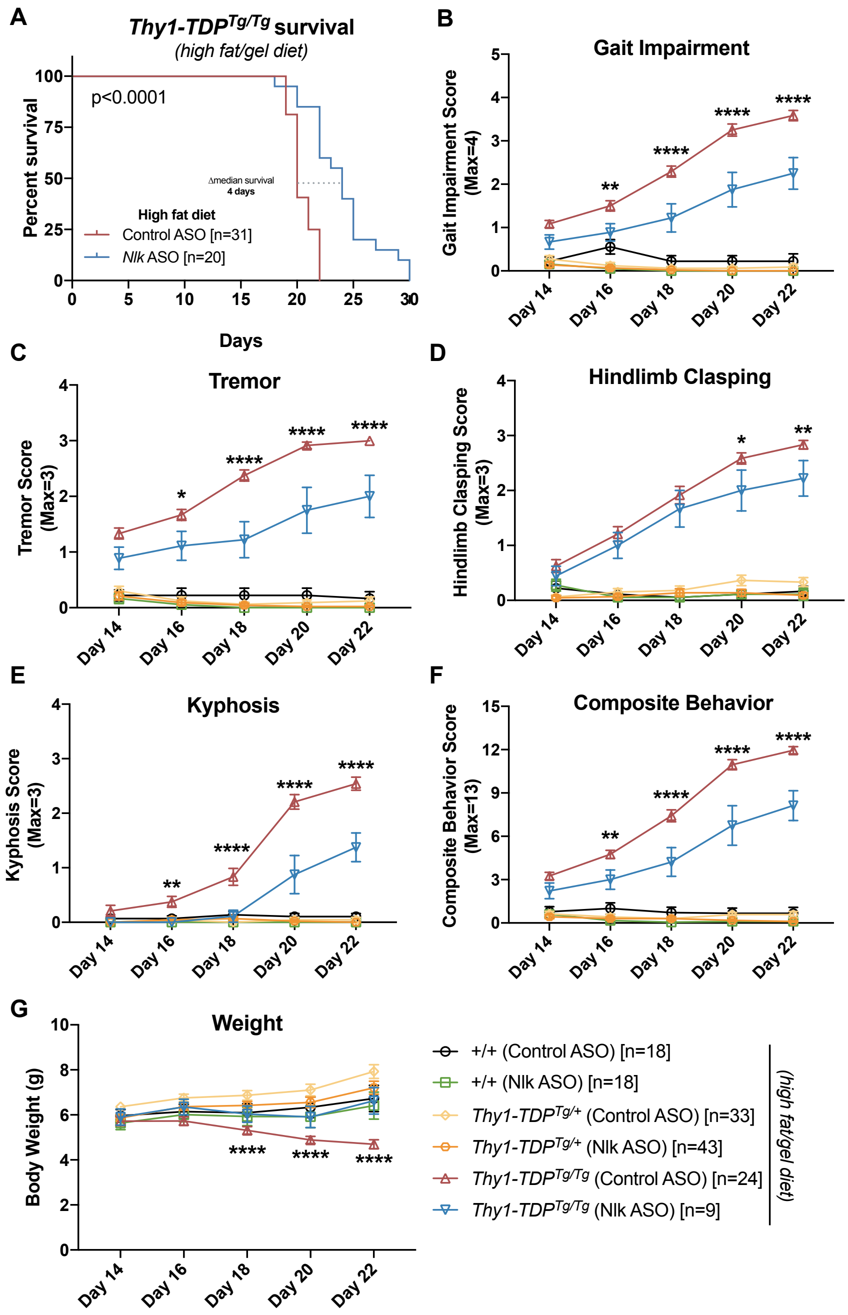

Fig. S10_Tejwani et al.

Supplemental Figure 10. ASOs targeting *Nlk* ameliorate motor behavior deficits in high fat/gel diet-fed *Thy1-TDP$^{Tg/Tg}$* mice.

(**A**) Kaplan-Meier survival curves showing administration of 10µg *Nlk* ASO at P1 increased *Thy1-TDP$^{Tg/Tg}$* animal survival. Curves were compared by log-rank test.

(**B-G**) *Nlk* ASO administration reduced gait impairment (**B**), tremor (**C**), hindlimb clasping (**D**), kyphosis (**E**), composite motor score (**F**), and prevented weight loss (**G**) in *Thy1-TDP$^{Tg/Tg}$* animals between P14-P22. One-way ANOVA analyses were performed to compare all listed genotypes/treatments per day unless otherwise noted and mean±SEM are displayed. *p<0.05, **p<0.01, ****p<0.0001.

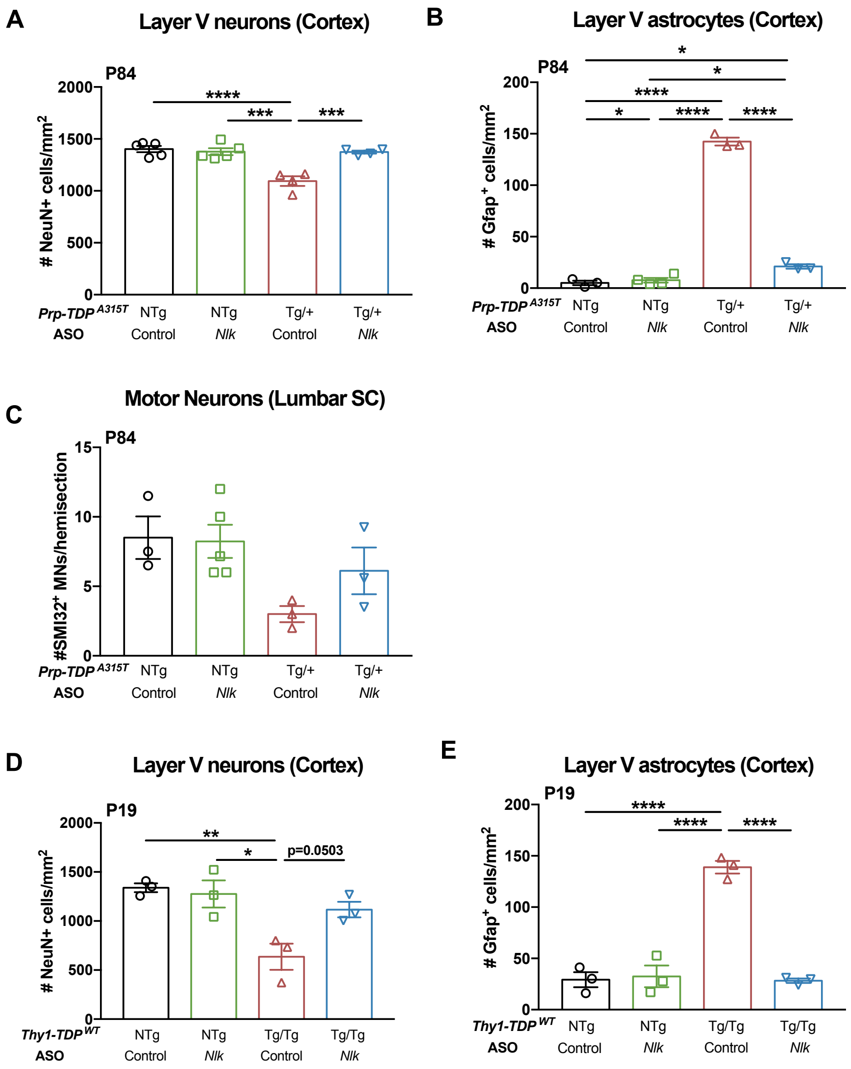

Fig. S11_Tejwani et al.

Supplemental Figure 11. ASOs targeting *Nlk* rescue pathology deficits in two TDP-43 mouse models. (**A-C**) Administration of 10µg of *Nlk* ASO at P1 rescued loss of layer V cortical neurons (**A**; n= 4-5 animals), layer V astrogliosis (**B**; n= 3-4 animals), and lumbar spinal cord (SC) motor neuron loss (**C**; n= 3-5 animals) in P84 *Prp-TDP*$^{A315T/+}$ male mice.

(**D** and **E**) 10µg of *Nlk* ASO at P1 rescued loss of layer V cortical neurons (**D**; n= 3 animals) and layer V astrogliosis (**E**; n= 3 animals) in *Thy1-TDP*$^{Tg/Tg}$ mice at P19.

One-way ANOVA analyses were performed to compare all listed genotypes/conditions and mean±SEM are displayed. *p<0.05, **p<0.01, ***p<0.001, ****p<0.0001.

Supplemental Table 1. Differential gene expression from RNA-seq of *Nlk* KO N2a cells. Processed and normalized differential gene expression data from RNA-seq comparing transcriptomes of *Nlk* KO N2a cells to isogenic controls. Mean expression data from triplicates were compared and FDR-adjusted p-value ($q<0.05$) was used to determine significance.

Supplemental Table 2. List of differentially expressed genes related to lysosomal function, autophagy, and stress granules from RNA-seq of *Nlk* KO N2a cells. Processed and normalized differential gene expression data from RNA-seq comparing transcripts of interest related to lysosomal function, autophagy, and stress granule biology from *Nlk* KO N2a cells RNA-seq. Mean expression data from triplicates were compared and FDR-adjusted p-value ($q<0.05$) was used to determine significance.

Supplemental Video 1. ASOs targeting *Nlk* partially rescue motor impairment in *Thy1-TDP$^{Tg/Tg}$* mice. *Thy1-TDP$^{Tg/Tg}$* mice injected with control ASO displayed a rapid stereotyped decline between P18 and P19, necessitating euthanasia. *Thy1-TDP$^{Tg/Tg}$* mice injected with 10μg of *Nlk* ASO at P1 retained motor abilities beyond P19.

Raw Western blots for Tejwani et al.

Figure 1E- Ifitm3

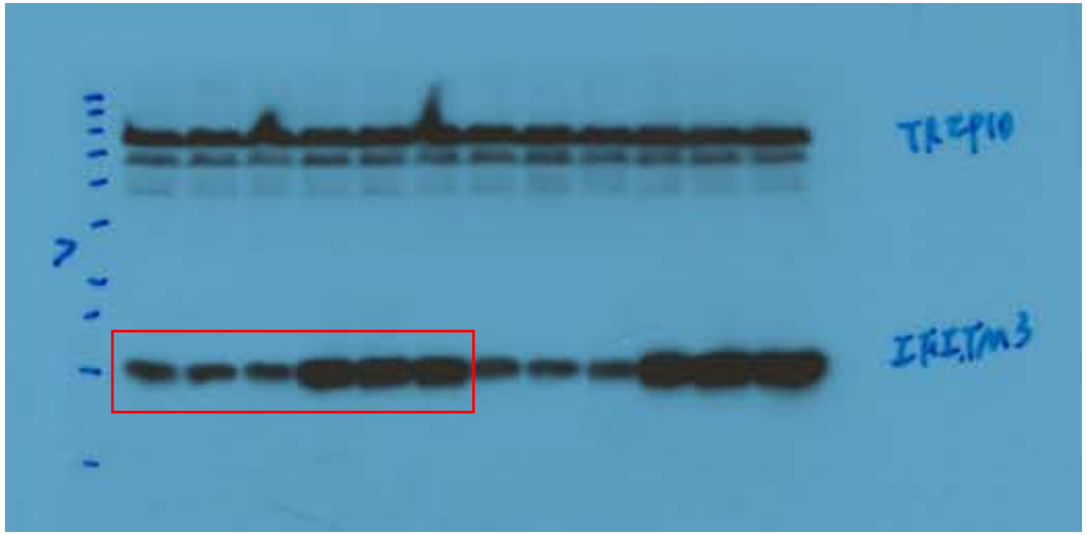

Figure 1E- Anxa6

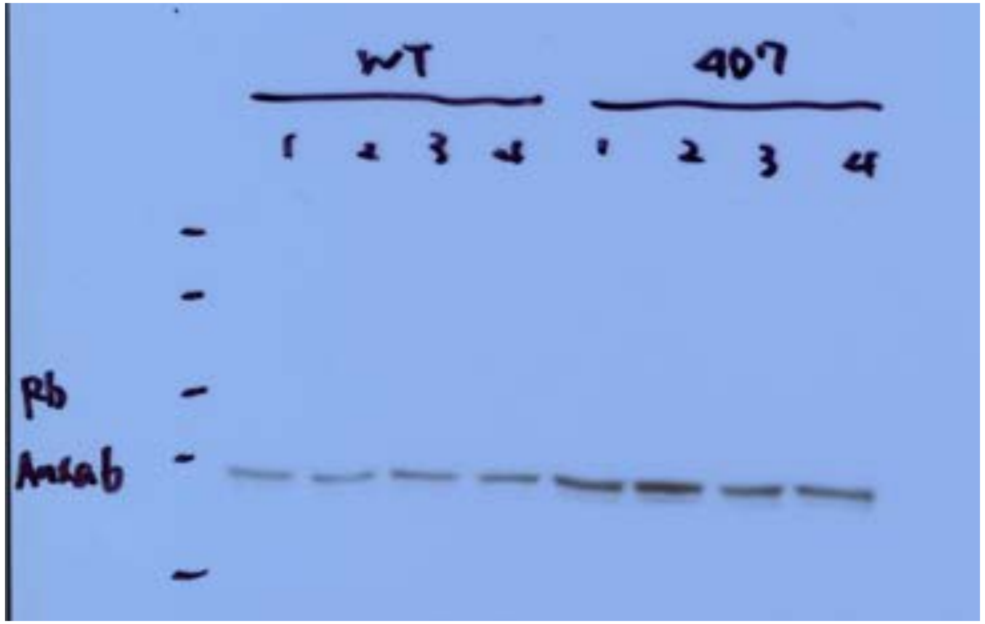

Figure 1E- Vinculin

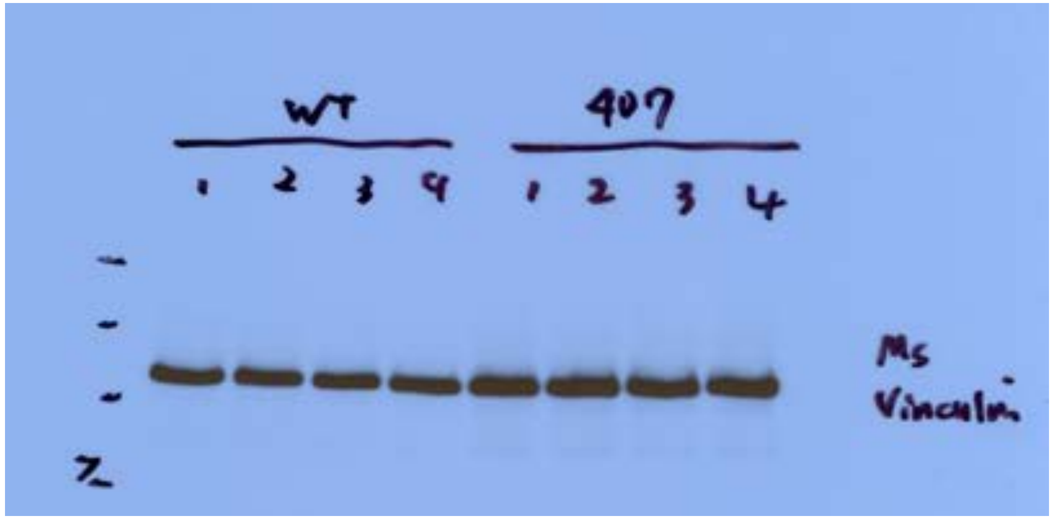

Figure 1E- Lamp2a

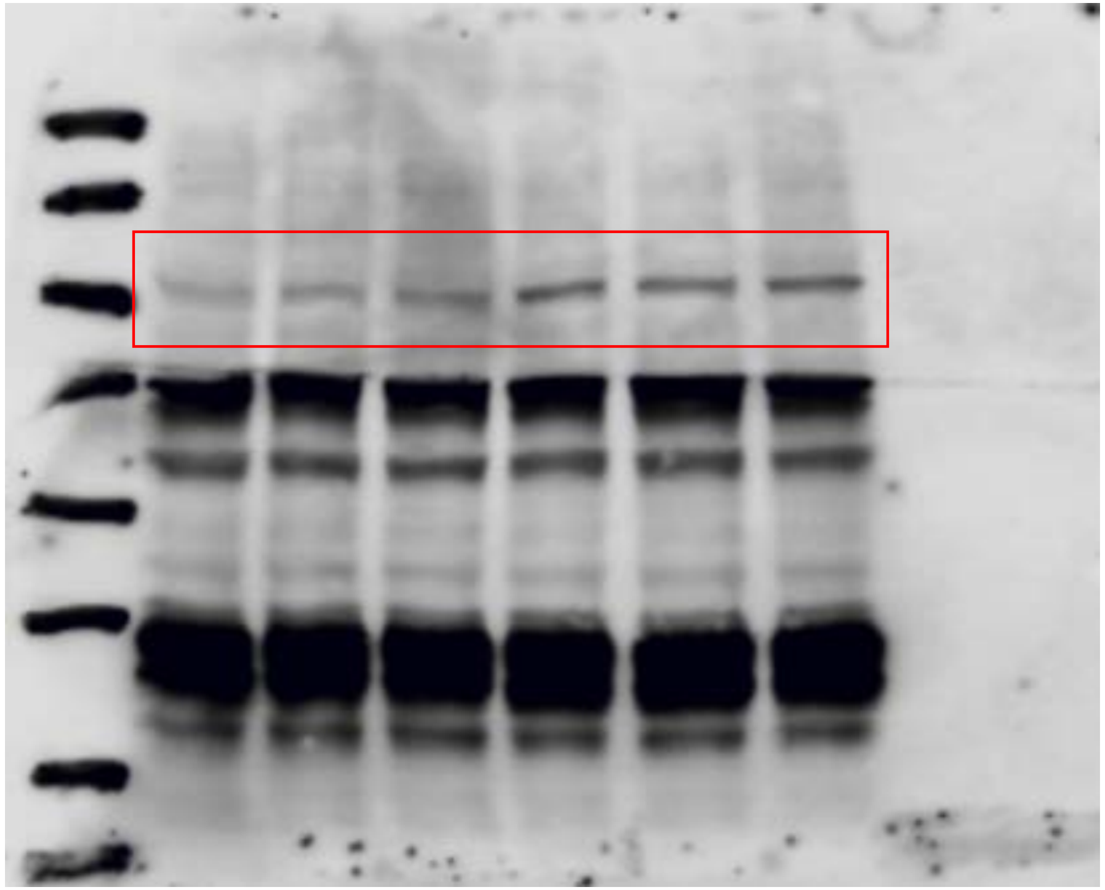

Figure 1E- CatD

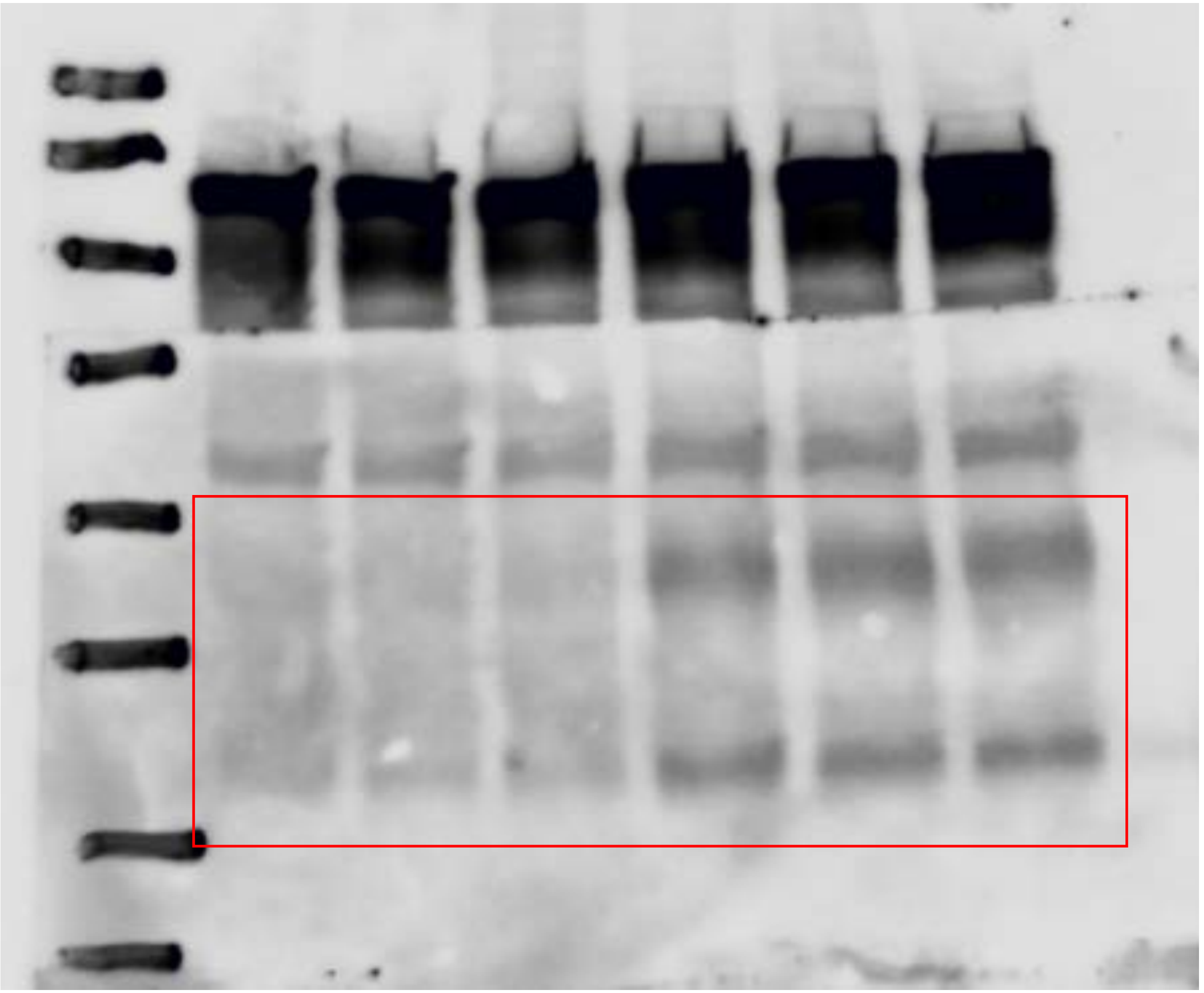

Figure 1E- Vinculin

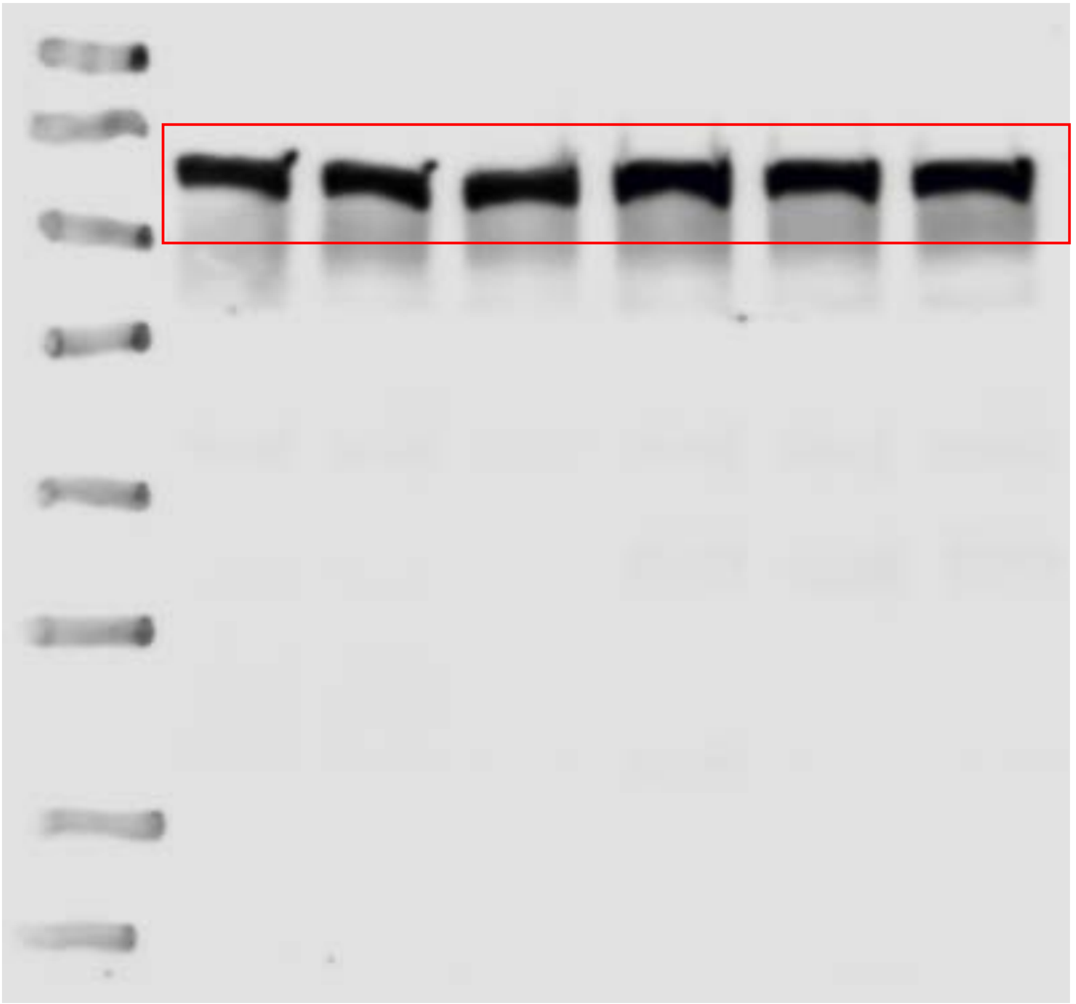

Figure 1E- Gapdh

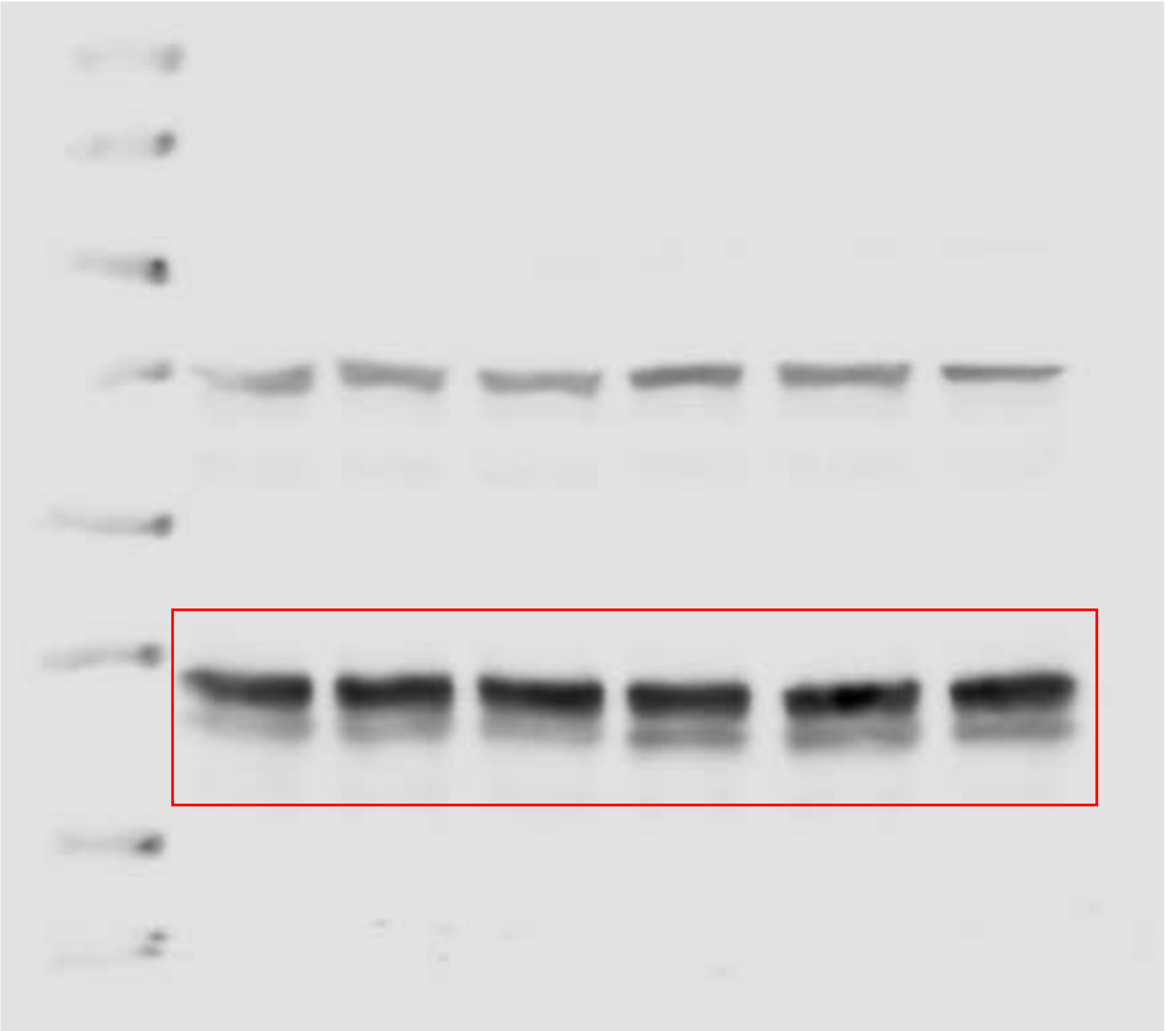

Figure 1M- LC3

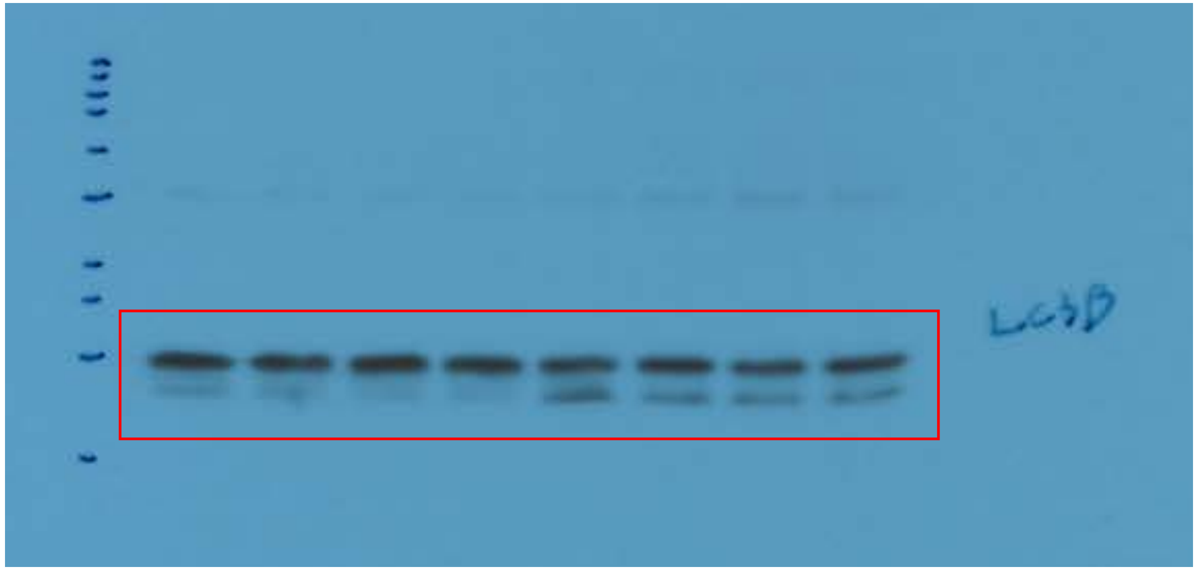

Figure 1M- p62

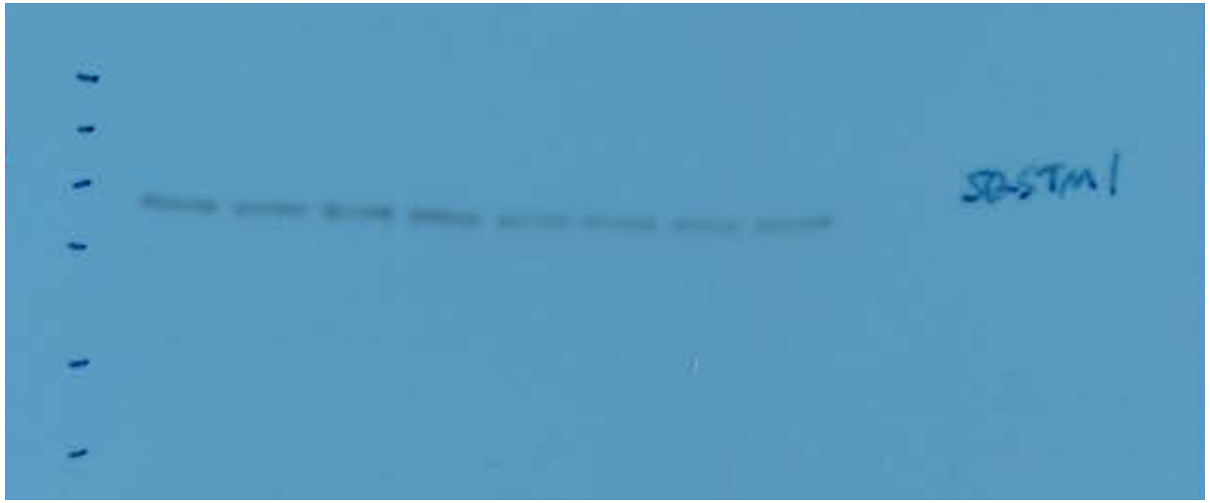

Figure 1M- Nlk

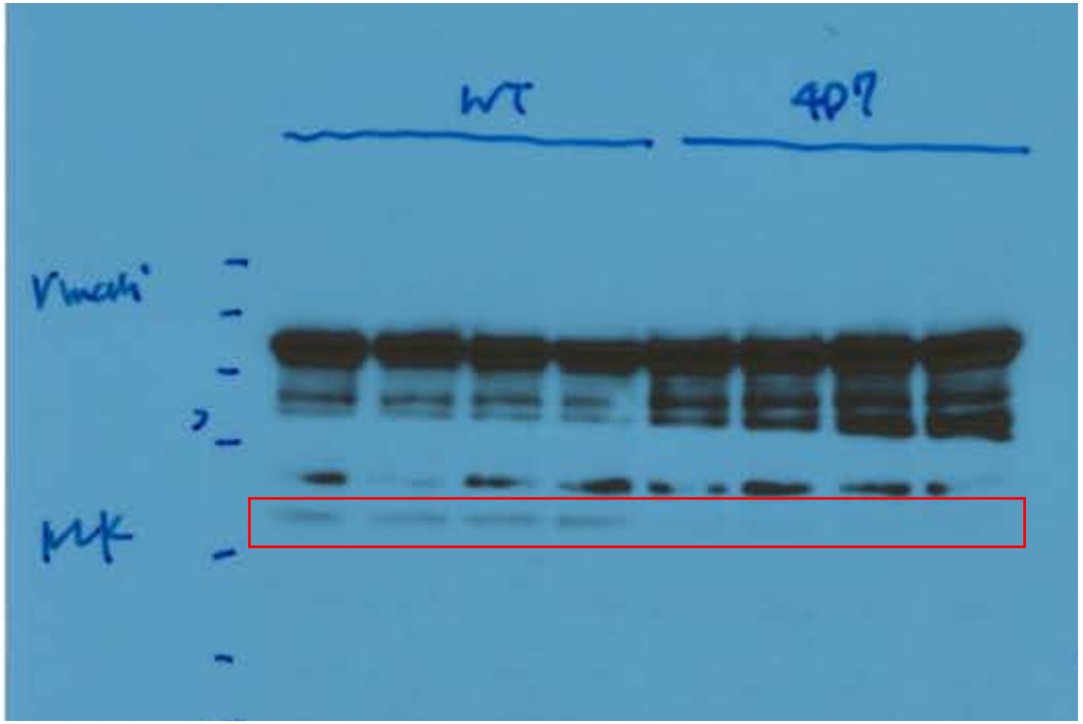

Figure 1M- Vinculin

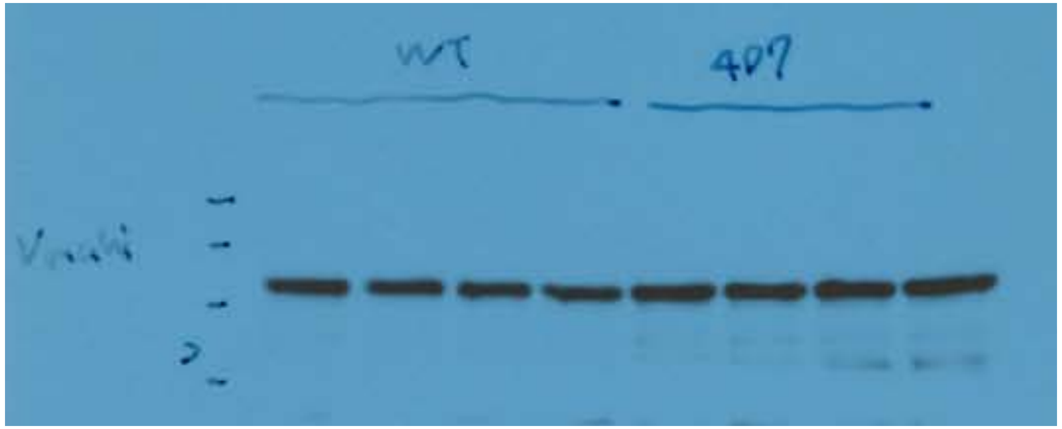

Figure 2A- Tfeb

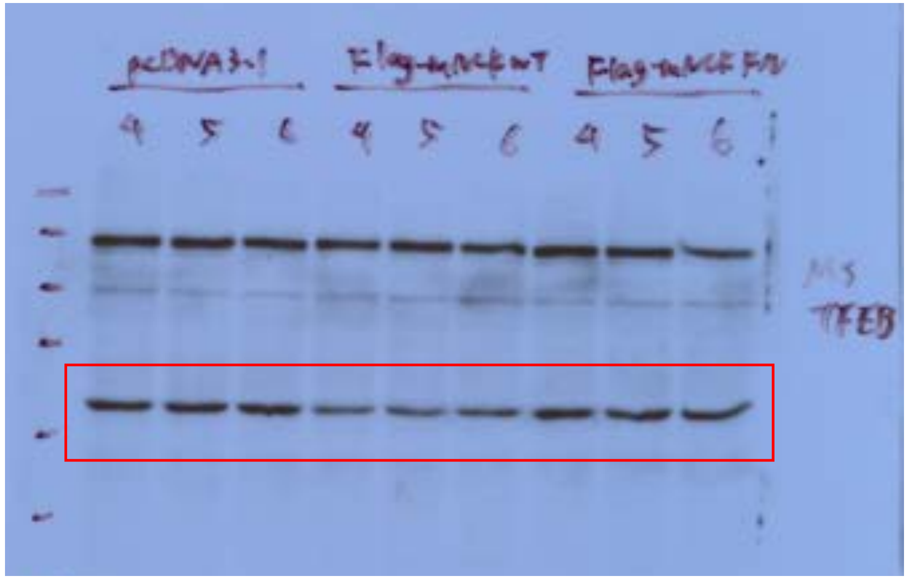

Figure 2A- Flag (Nlk)

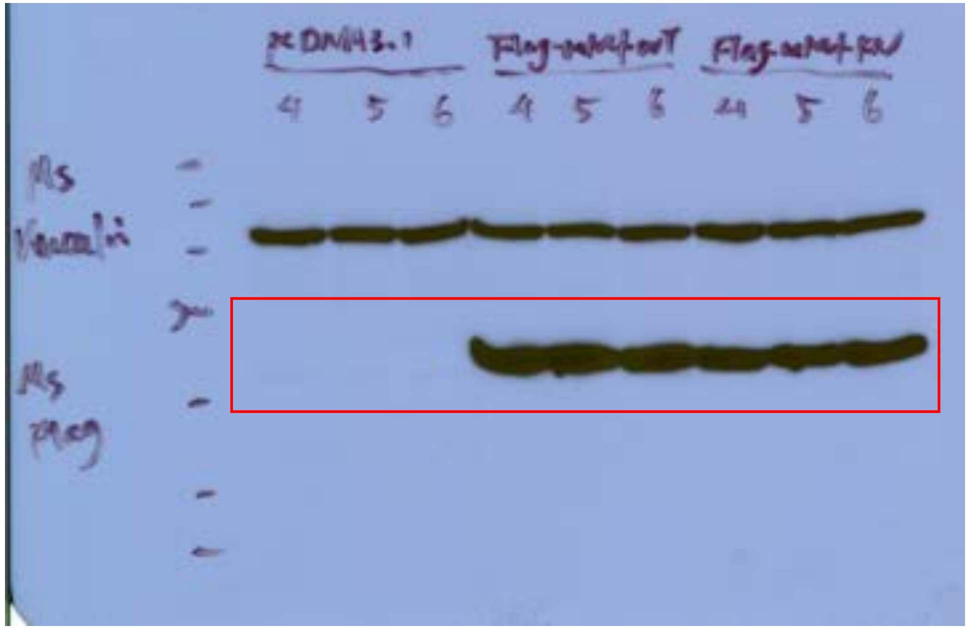

Figure 2A- Vinculin

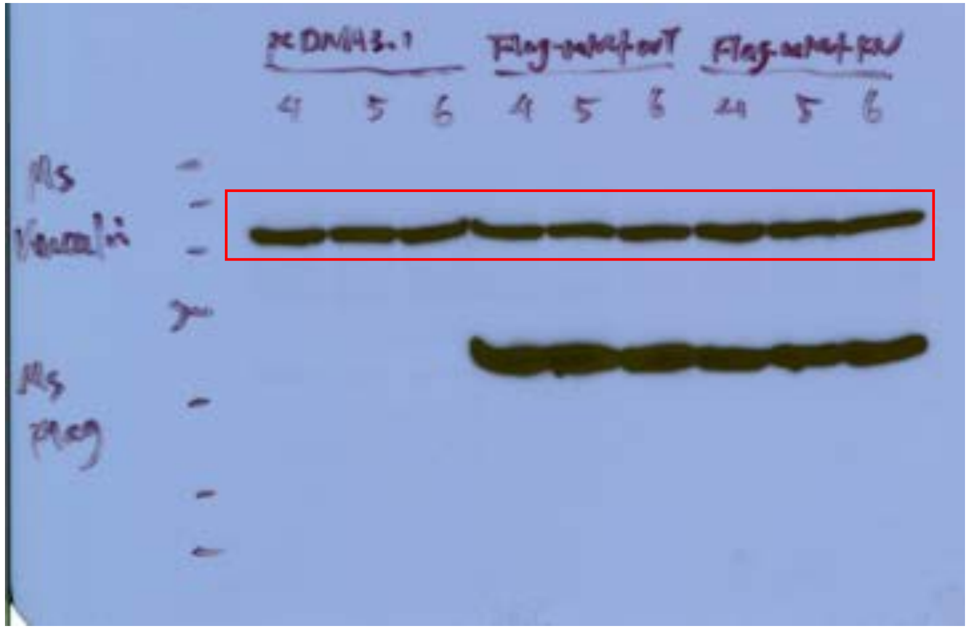

Figure 2C- Tfeb (Cytosol)

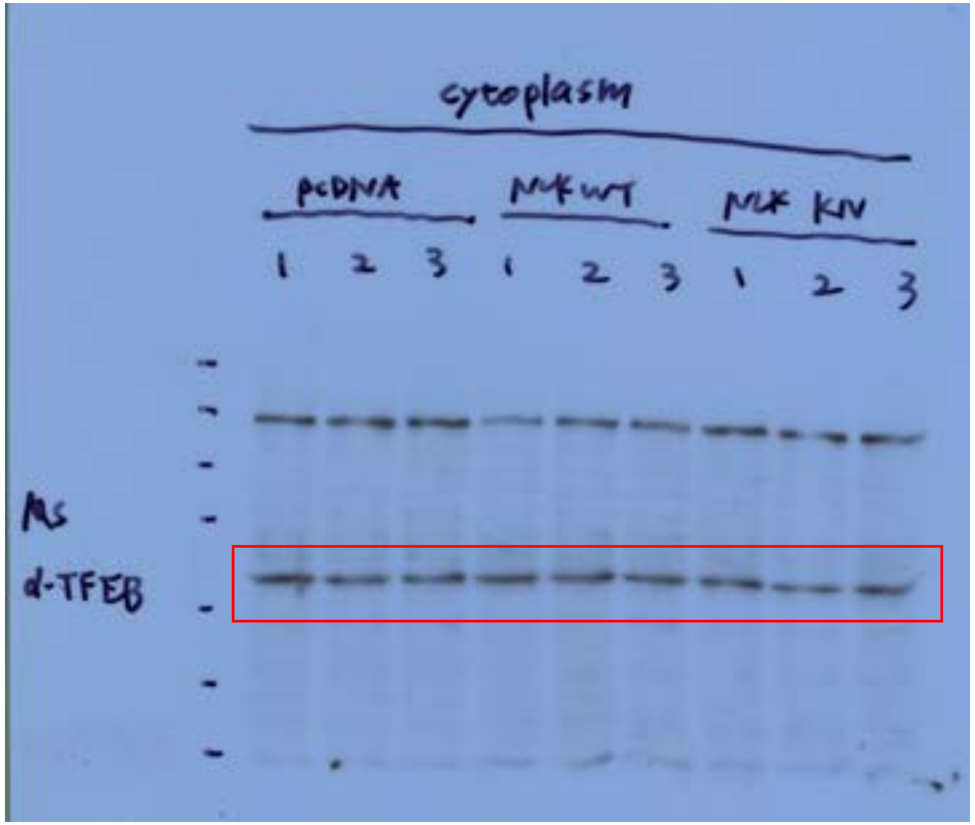

Figure 2C- Tubulin (Cytosol)

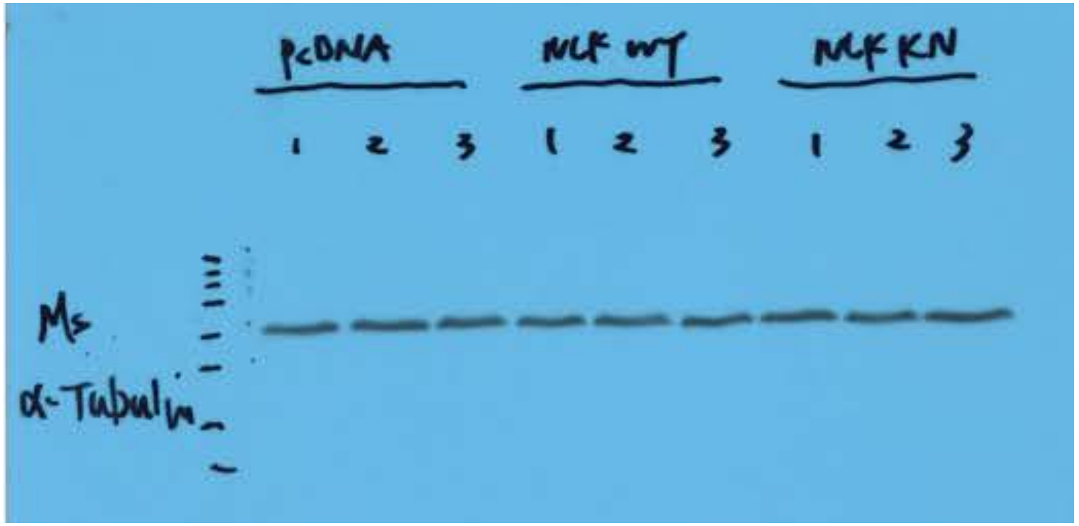

Figure 2C- Tfeb (Nucleus)

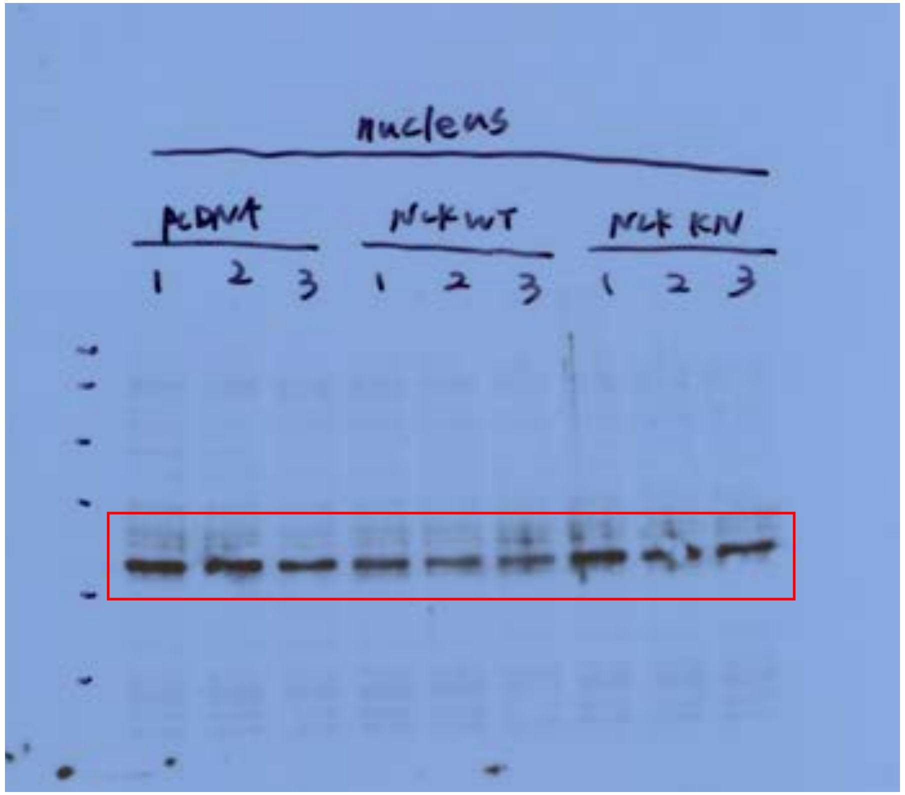

Figure 2C- Histone H3 (Nucleus)

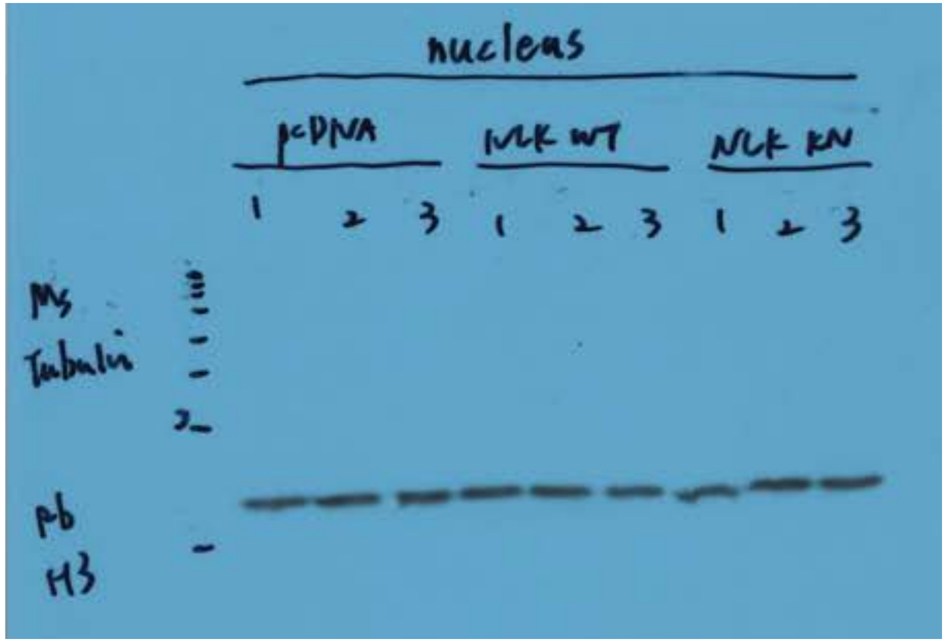

Figure 2E- Tfeb (DMSO)

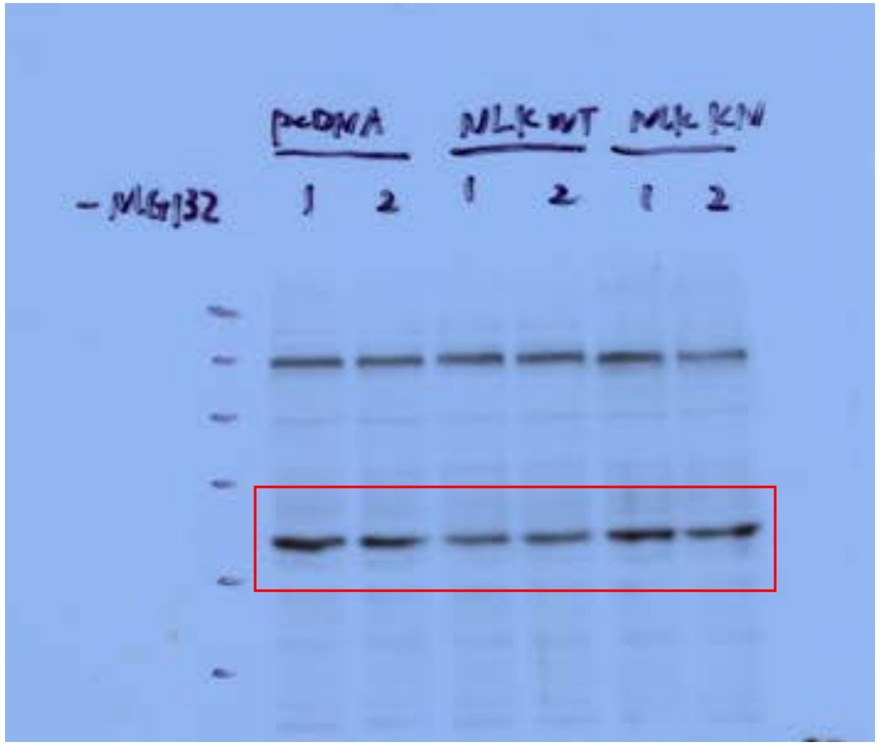

Figure 2E- Tfeb MG132

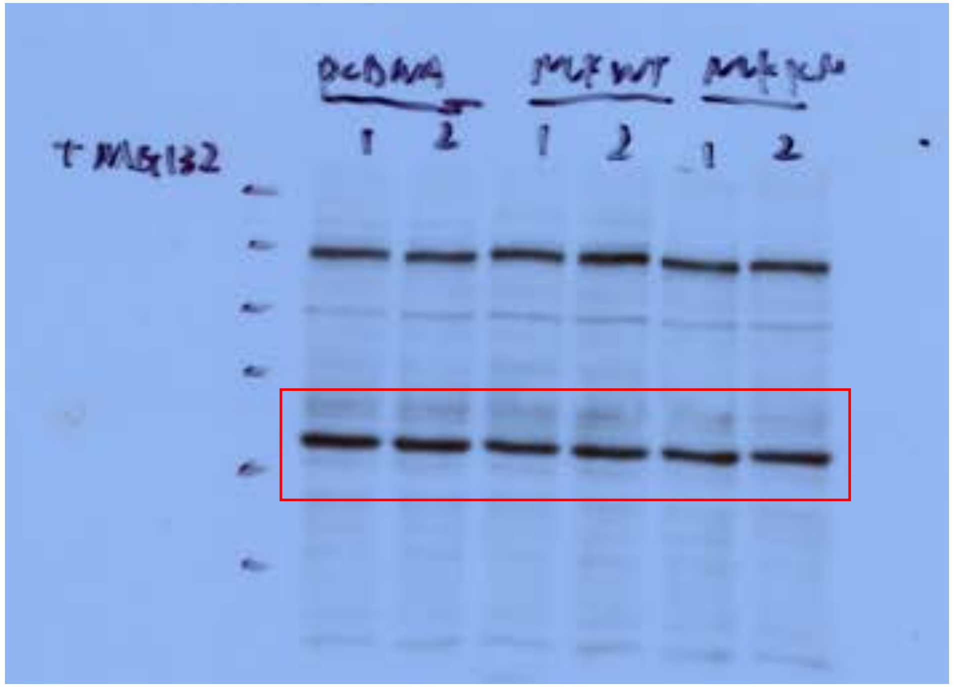

Figure 2E- Flag DMSO

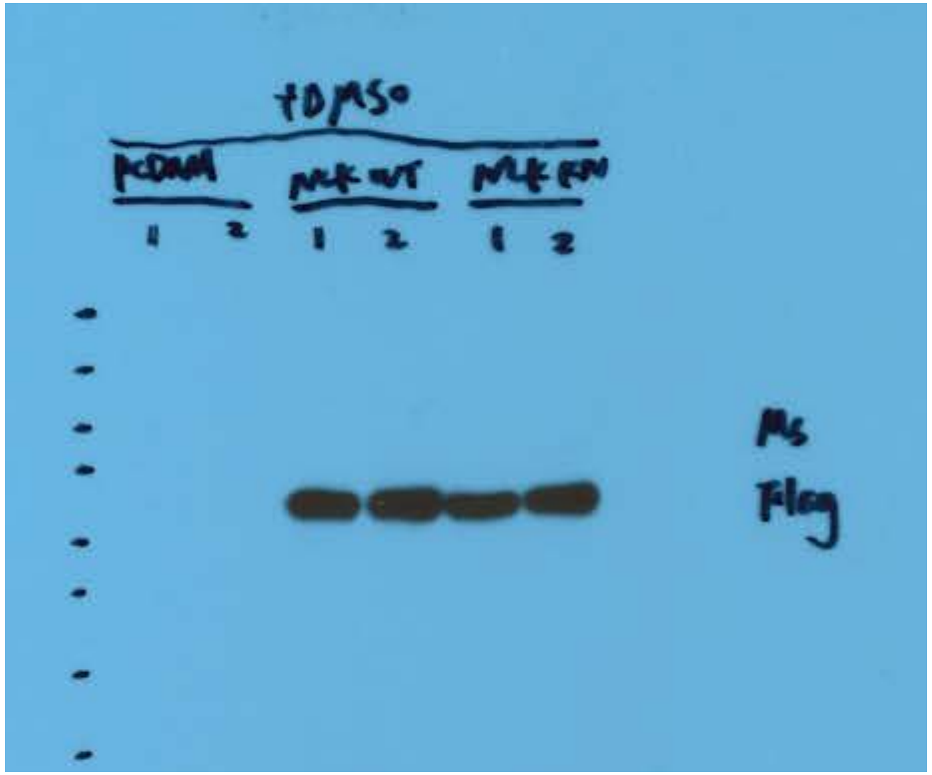

Figure 2E- Flag MG132

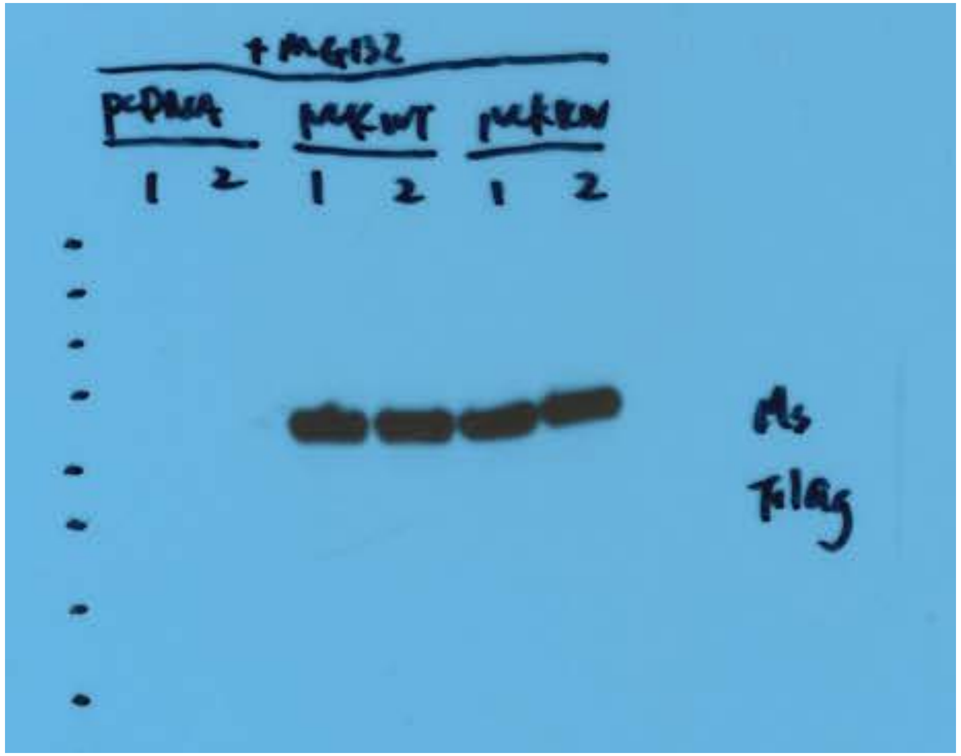

Figure 2E- Vinculin DMSO

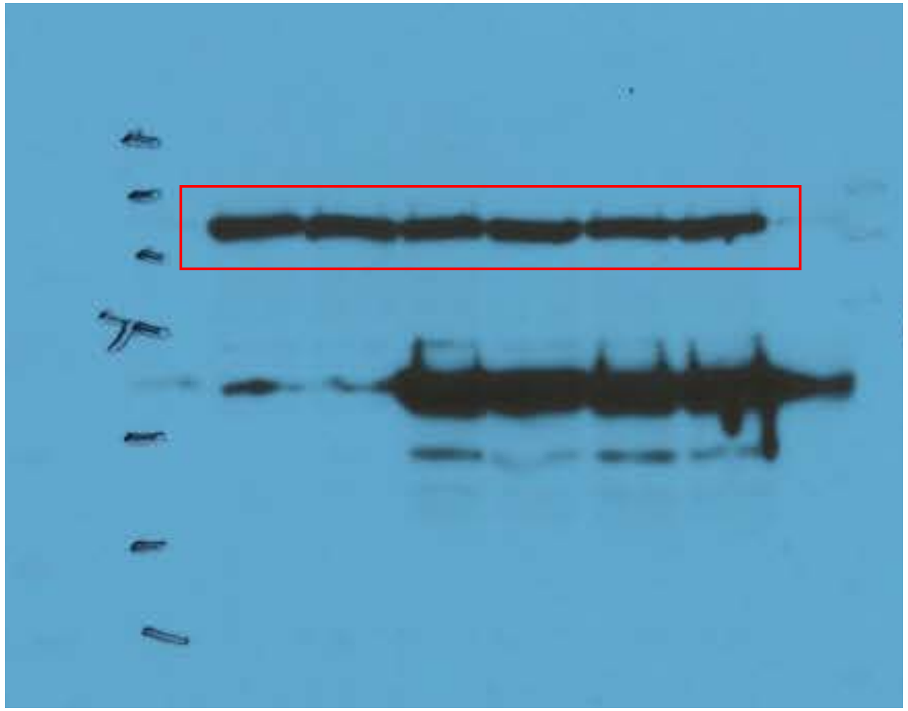

Figure 2E- Vinculin MG132

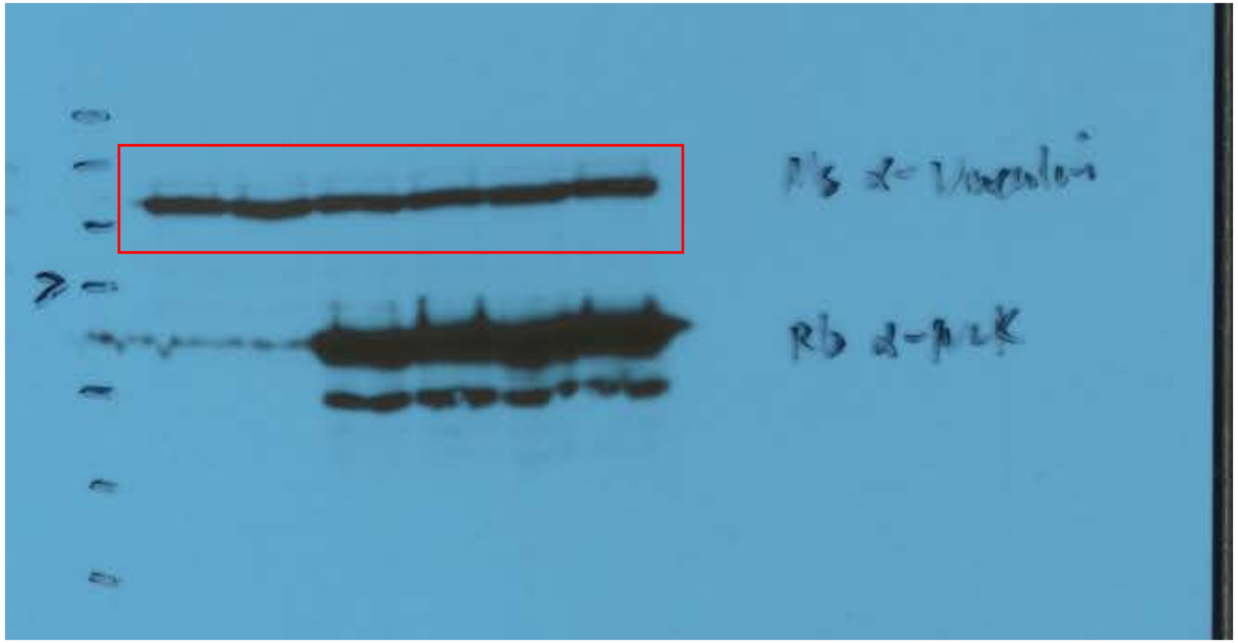

Figure 2G- p-Tfeb (S122) cytosol

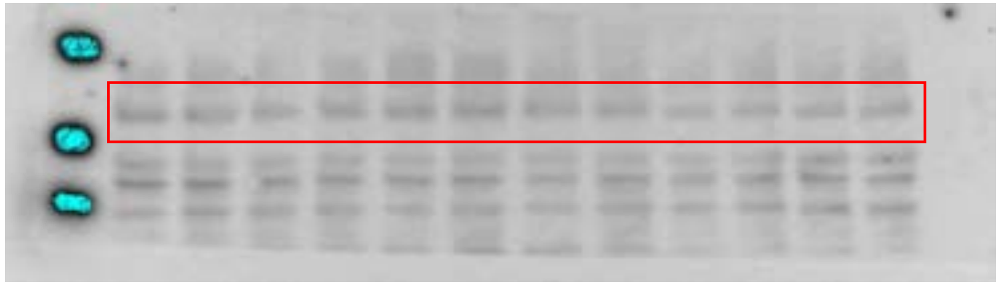

Figure 2G- p-Tfeb (S122) nucleus

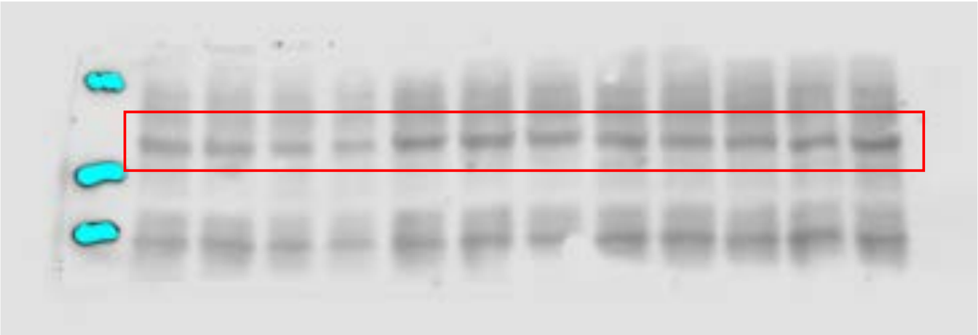

Figure 2G- p-Tfeb (S142) cytosol

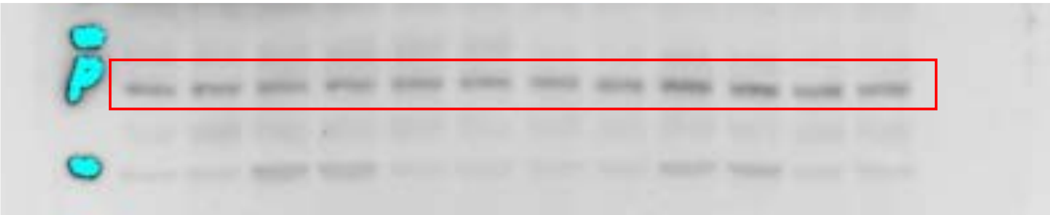

Figure 2G- p-Tfeb (S142) nucleus

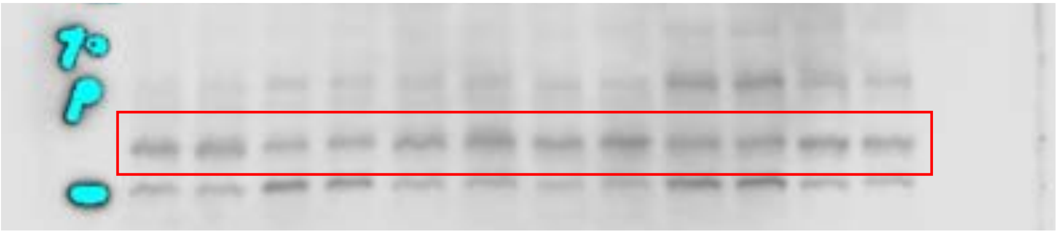

Figure 2G- Tfeb cytosol

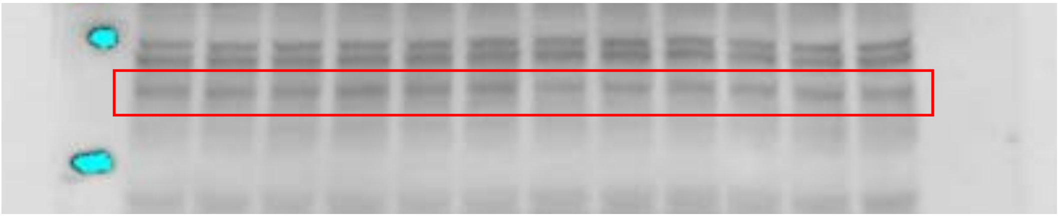

Figure 2G- Tfeb nucleus

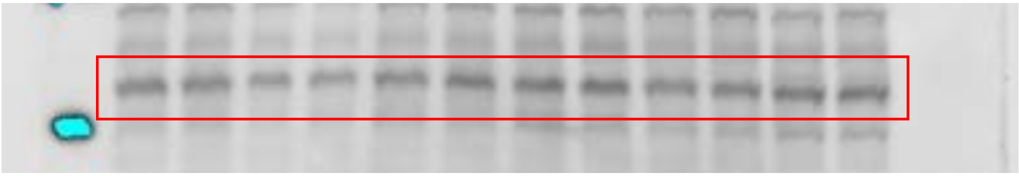

Figure 2G- Flag cytosol

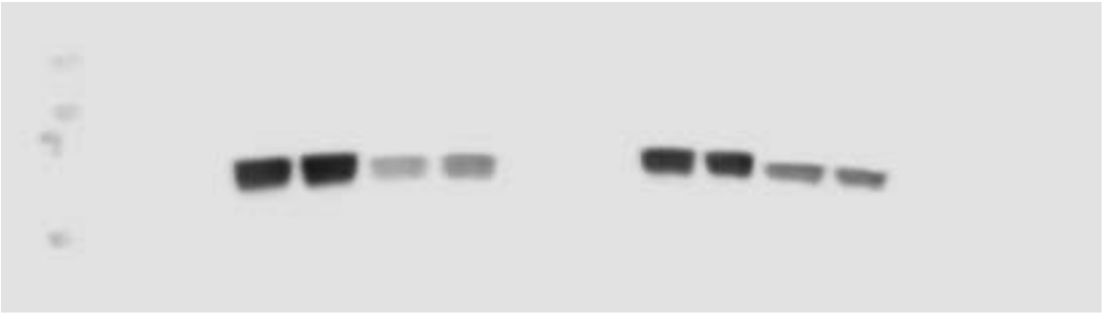

Figure 2G- Flag nucleus

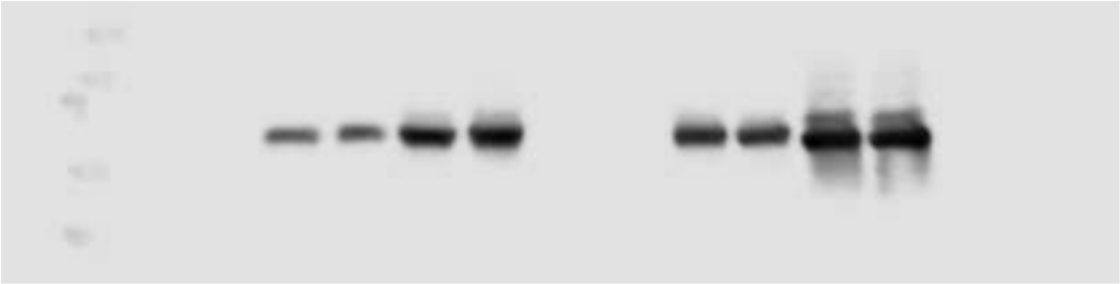

Figure 2G- Vinculin cytosol

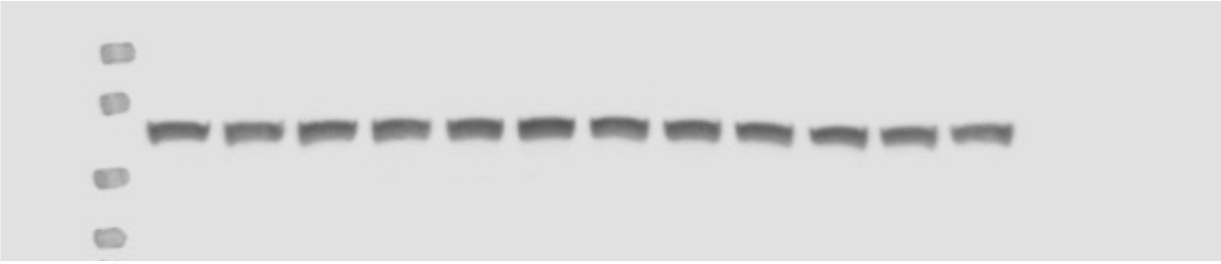

Figure 2G- Vinculin nucleus

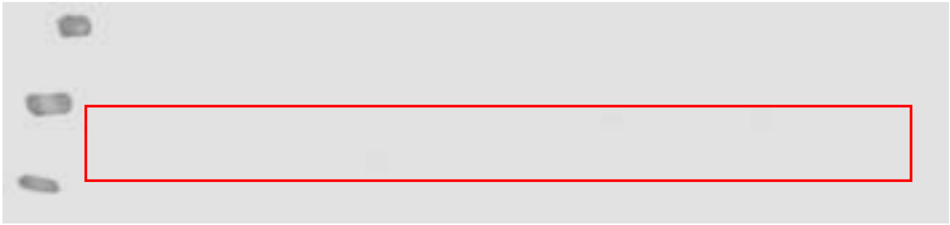

Figure 2G- Histone H3 cytosol

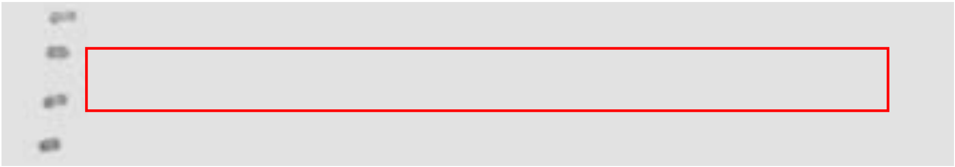

Figure 2G- Histone H3 nucleus

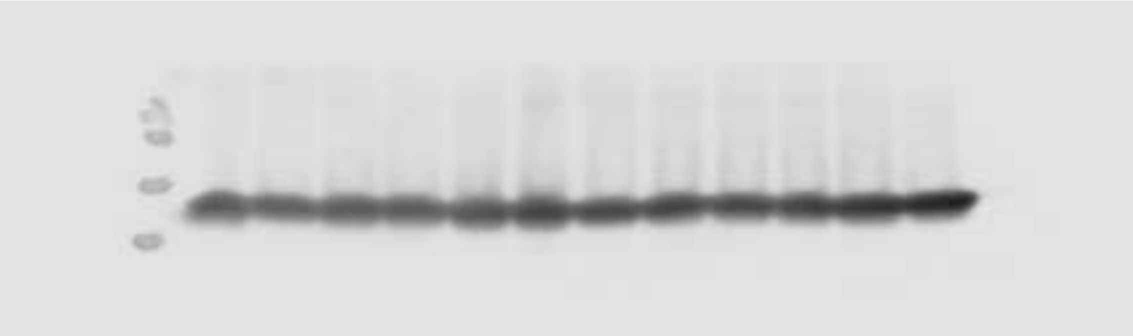

Figure 3A- Vinculin

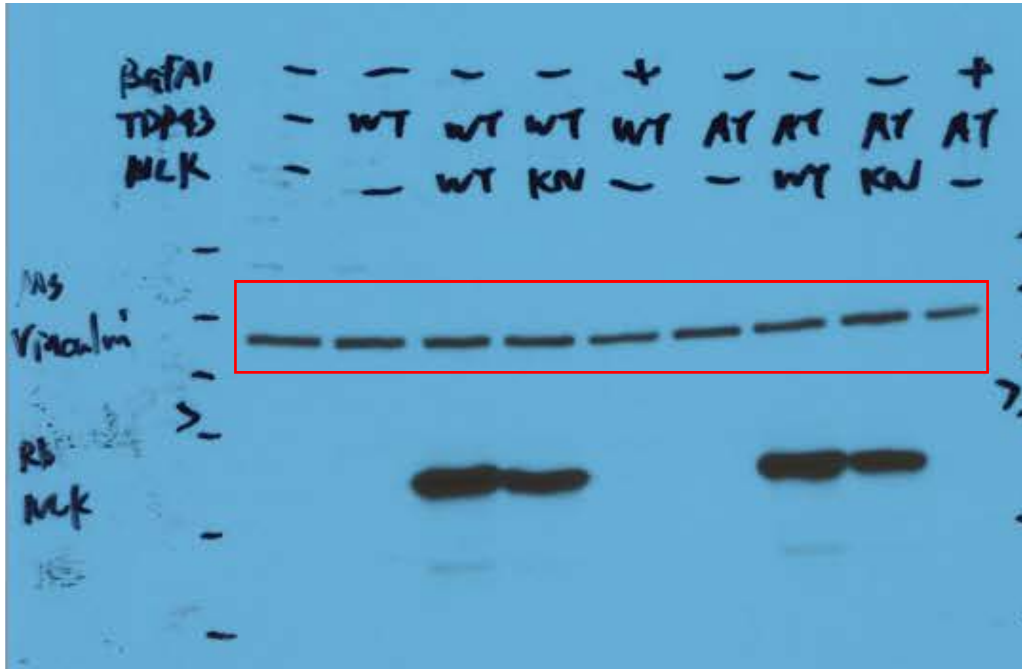

Figure 3A- Nlk

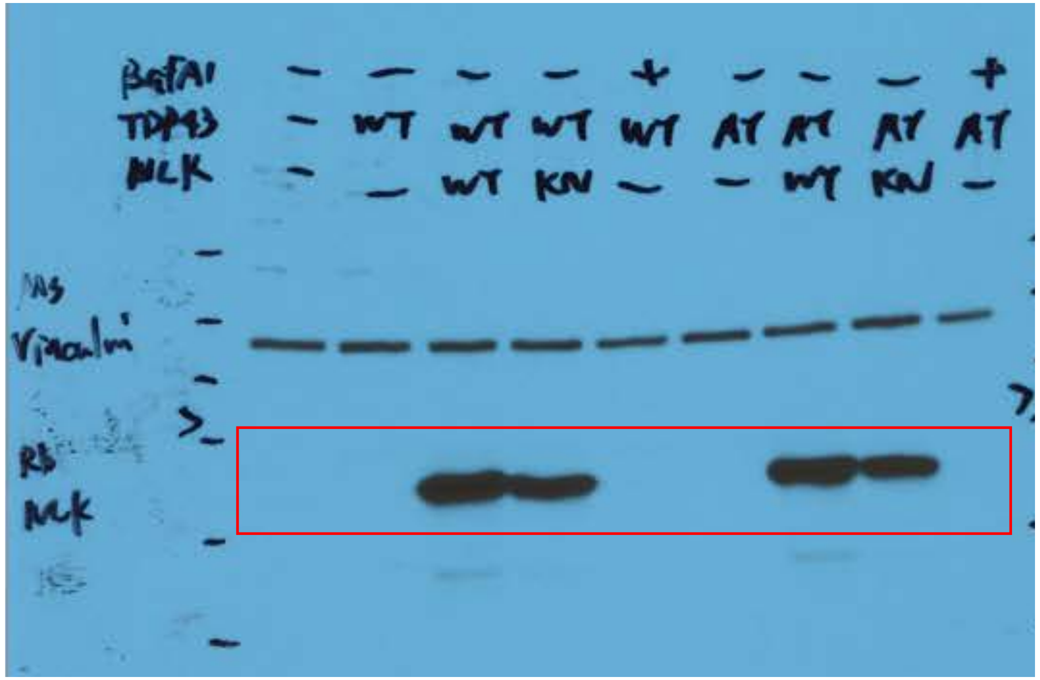

Figure 3A- Total TDP-43 (low exposure)

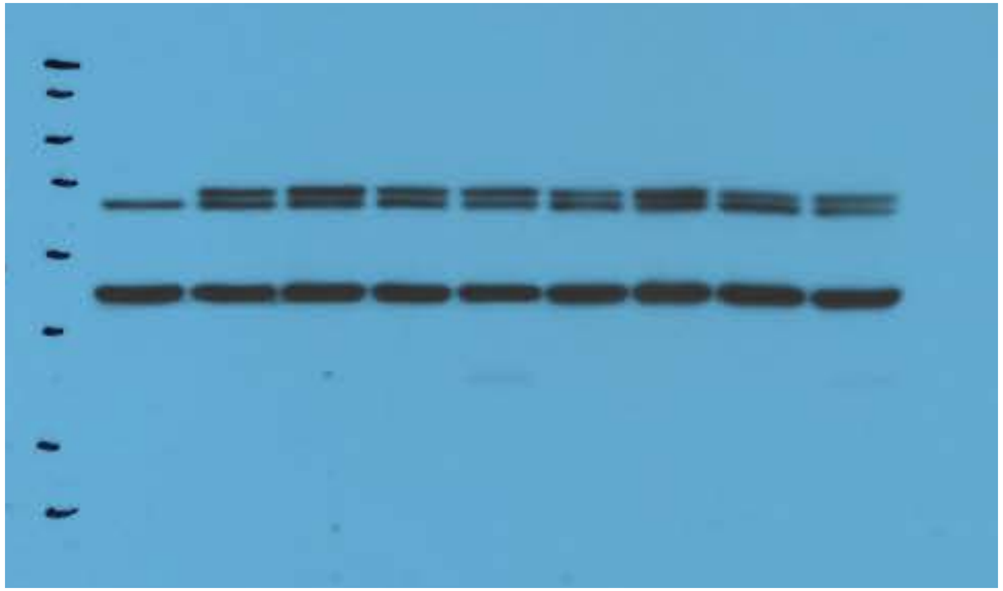

Figure 3A- Total TDP-43 (high exposure)

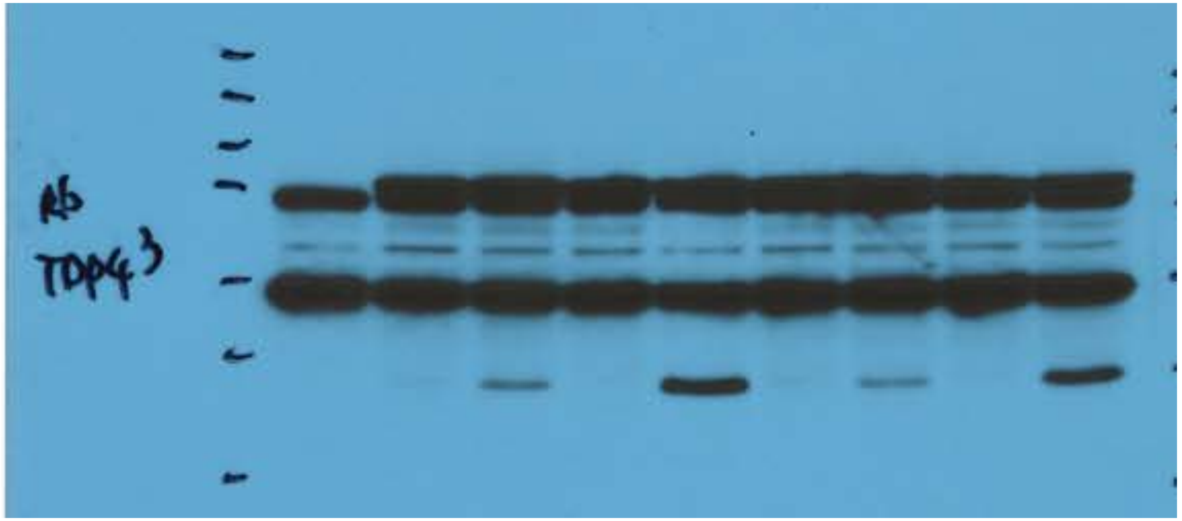

Figure 3C- Vinculin cytoplasm (LS)

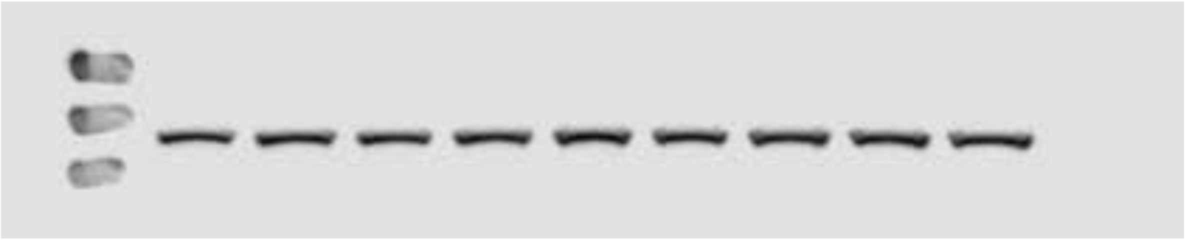

Figure 3C- Vinculin nucleus (LS)

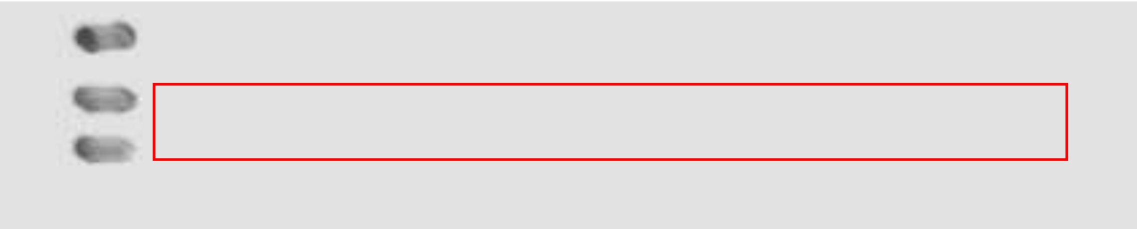

Figure 3C- Histone H3 cytoplasm (LS)

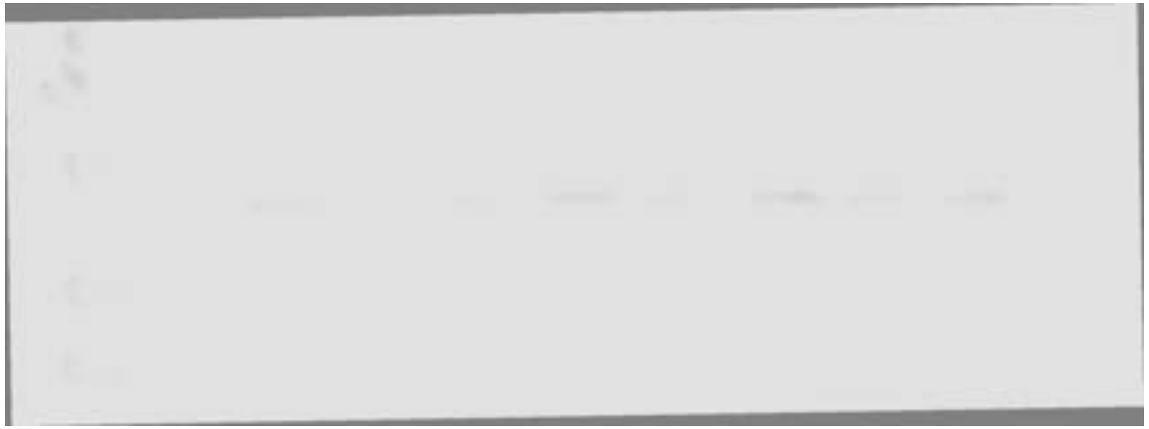

Figure 3C- Histone H3 nucleus (LS)

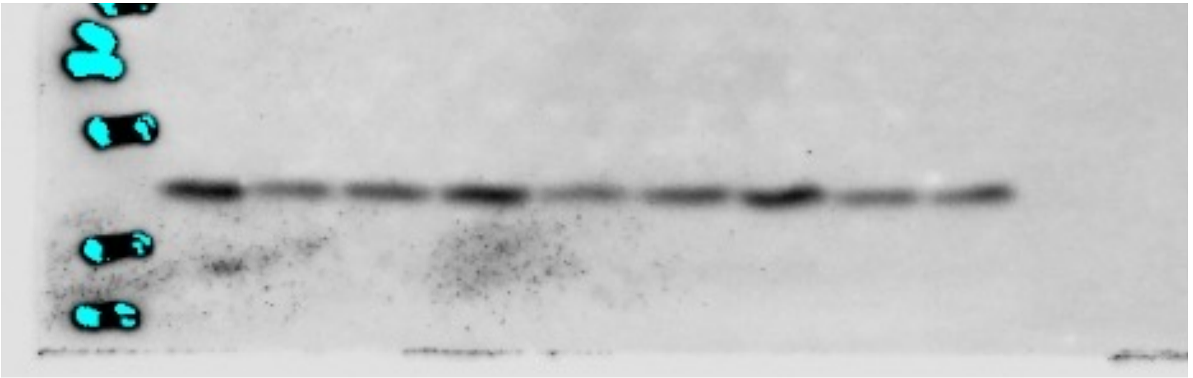

Figure 3C- Flag cytoplasm (LS)

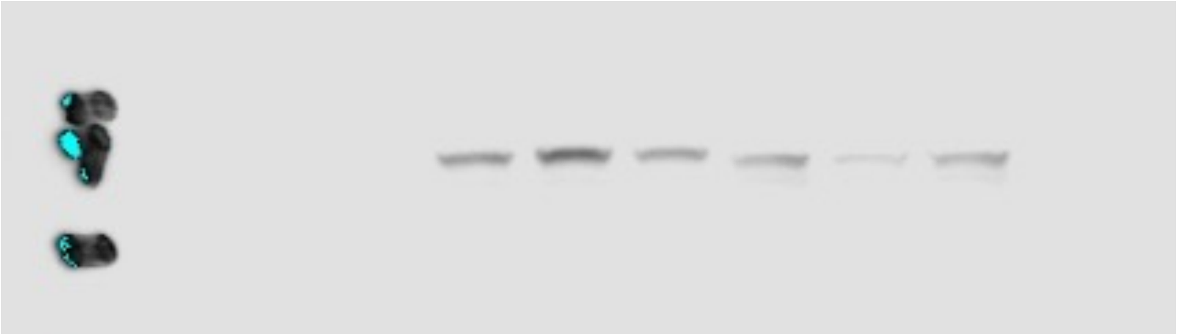

Figure 3C- Flag nucleus (LS)

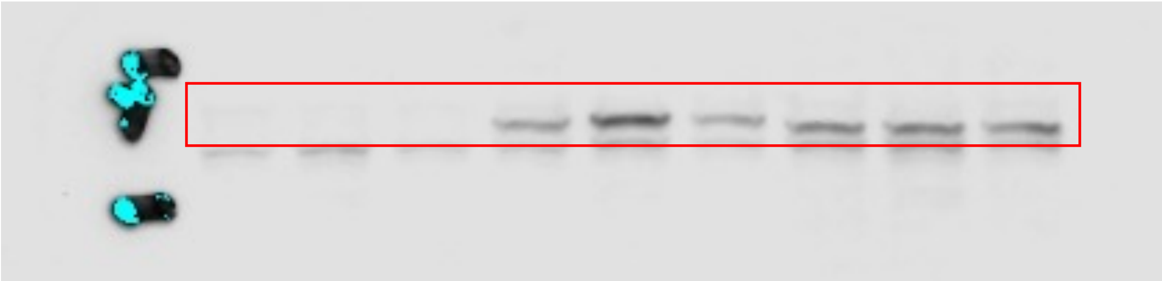

Figure 3C- hTDP-43 cytoplasm (LS)

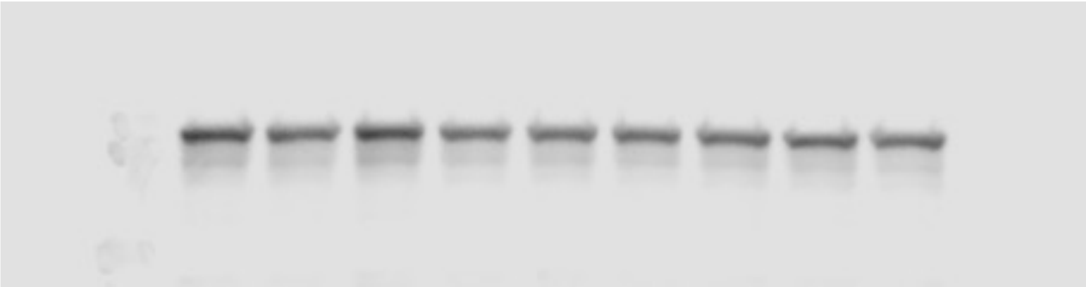

Figure 3C- hTDP-43 nucleus (LS)

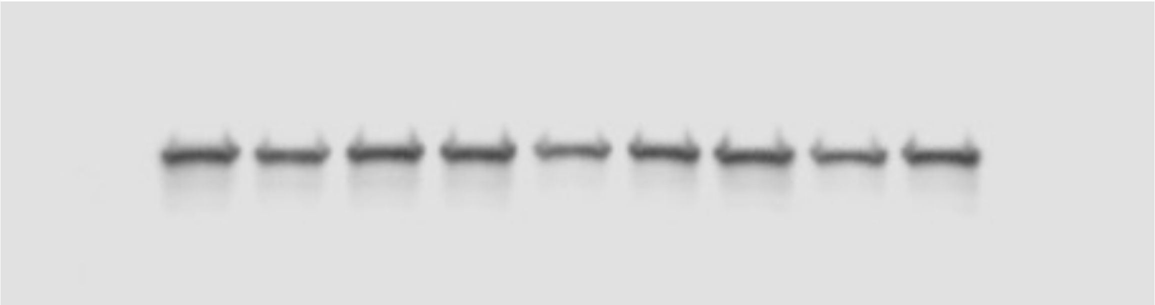

Figure 3C- hTDP-43 cytoplasm (SK)

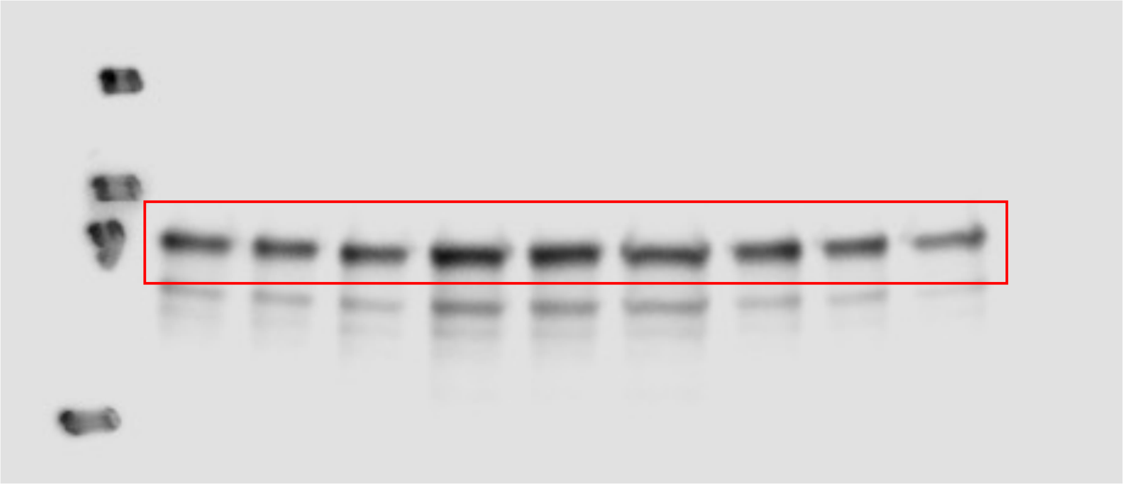

Figure 3C- hTDP-43 nucleus (SK)

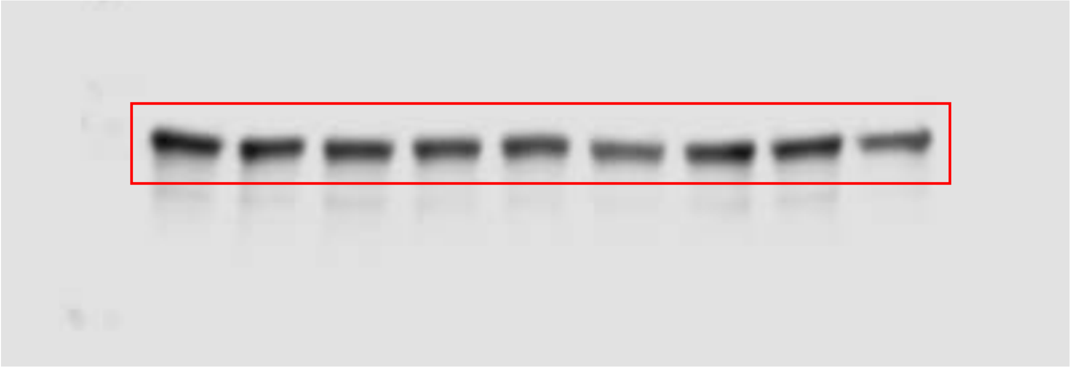

Figure 3C- hTDP-43 cytoplasm (UR)

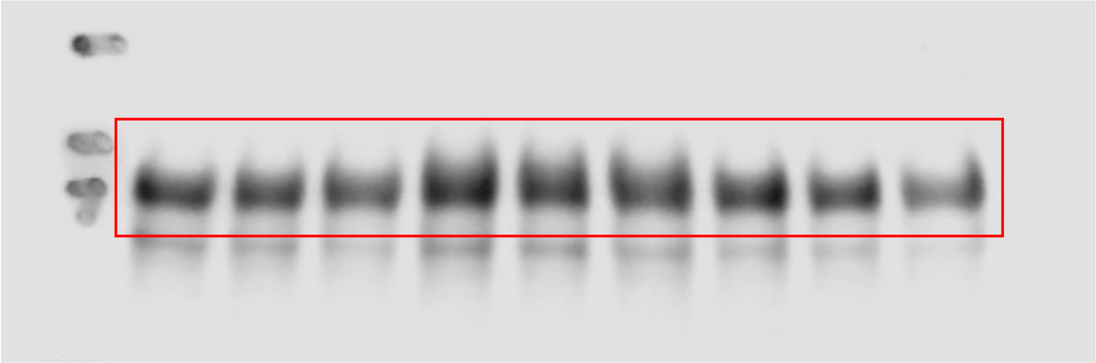

Figure 3C- hTDP-43 nucleus (UR)

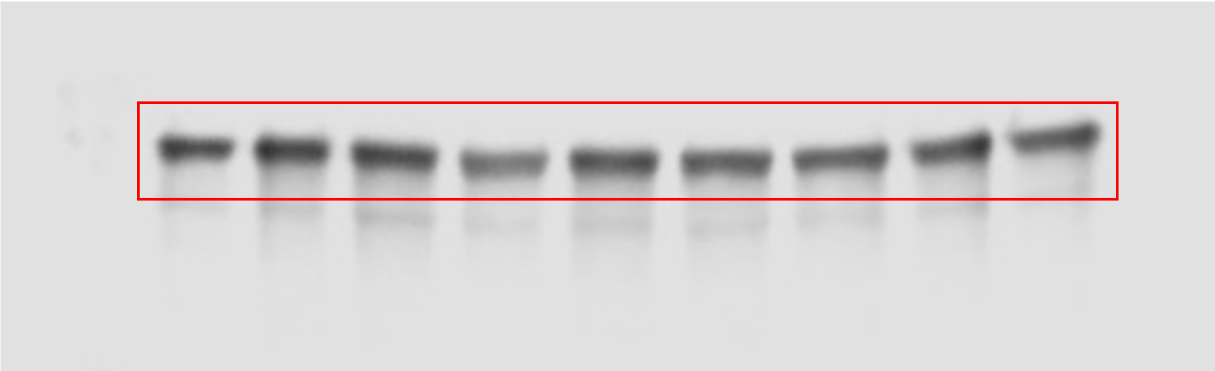

Figure 3G- Total TDP-43 (low exposure)

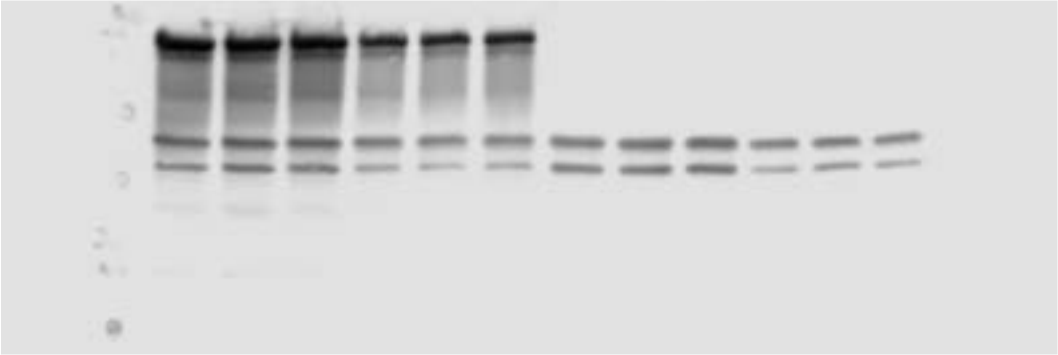

Figure 3G- Total TDP-43 (high exposure)

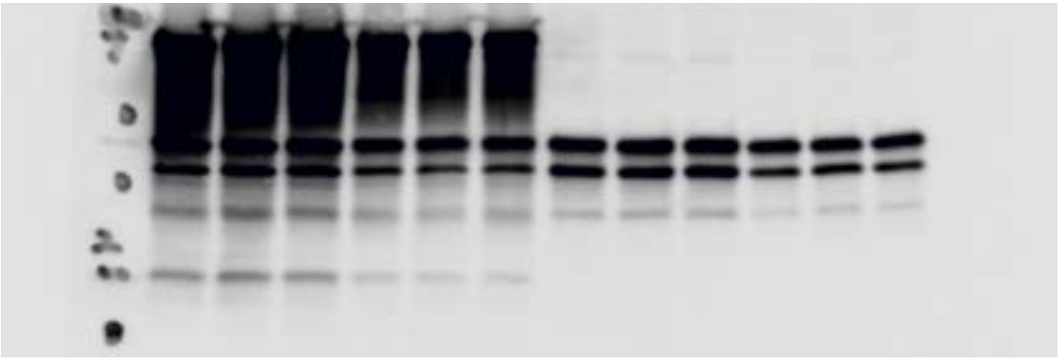

Figure 3G- Nlk

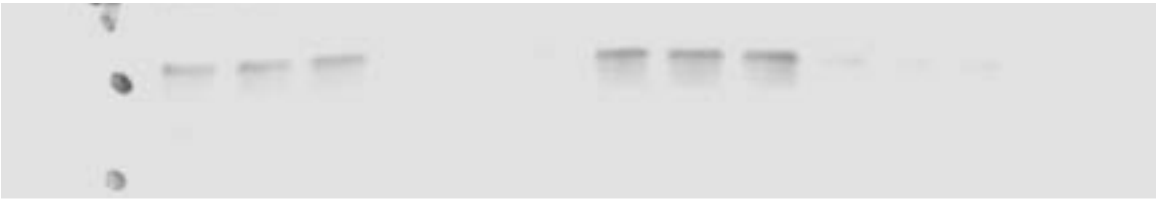

Figure 3G- Vinculin

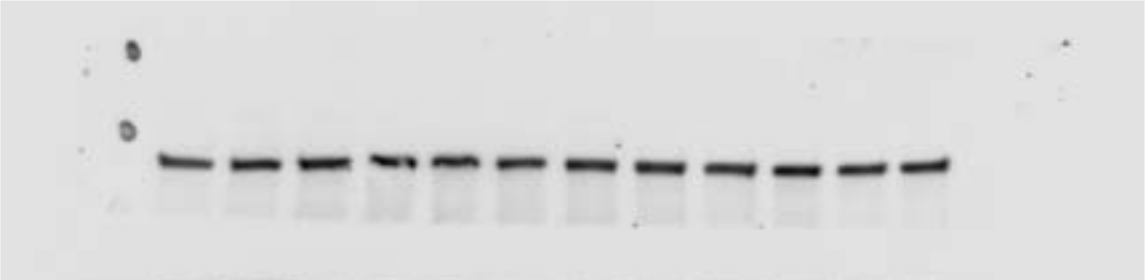

Figure 4G- Vinculin (LS)

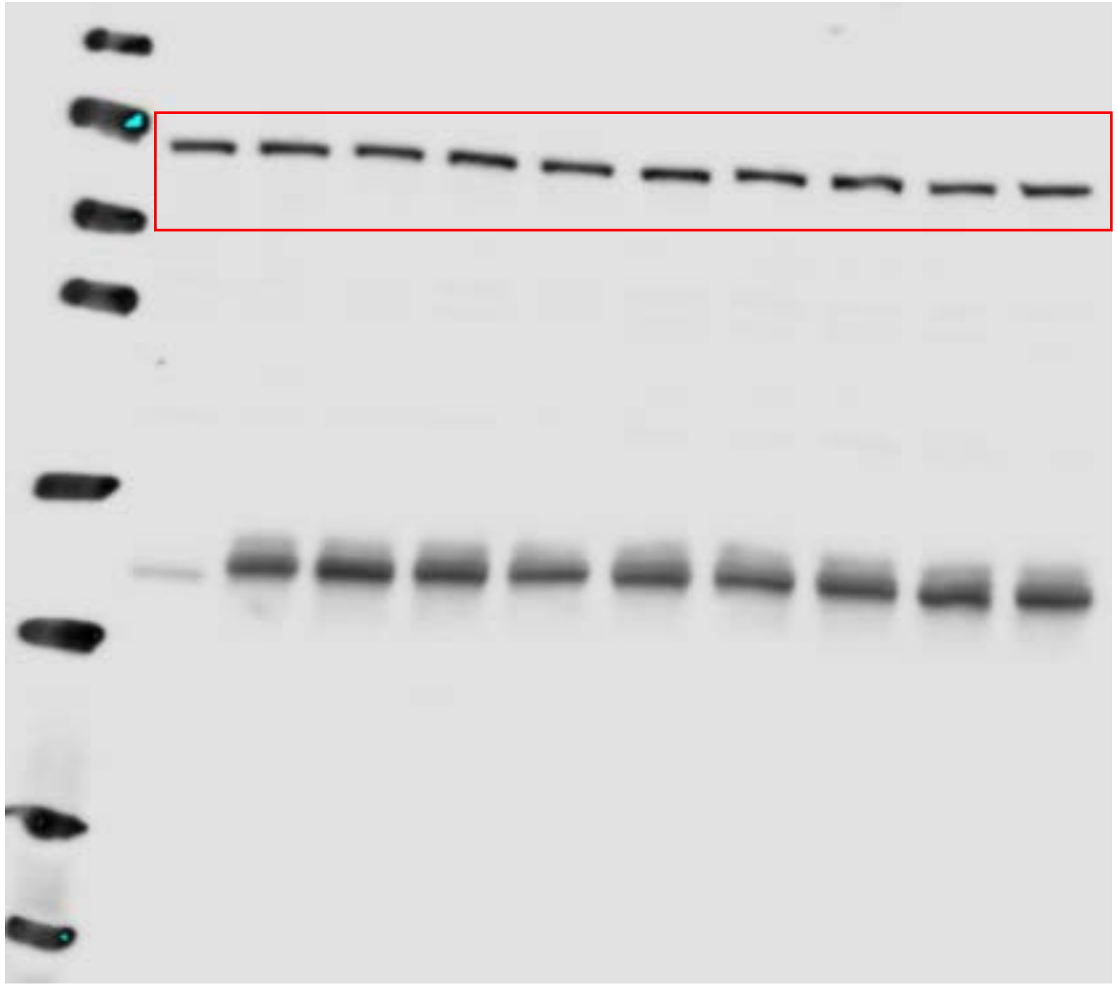

Figure 4G- TDP-43 (LS)

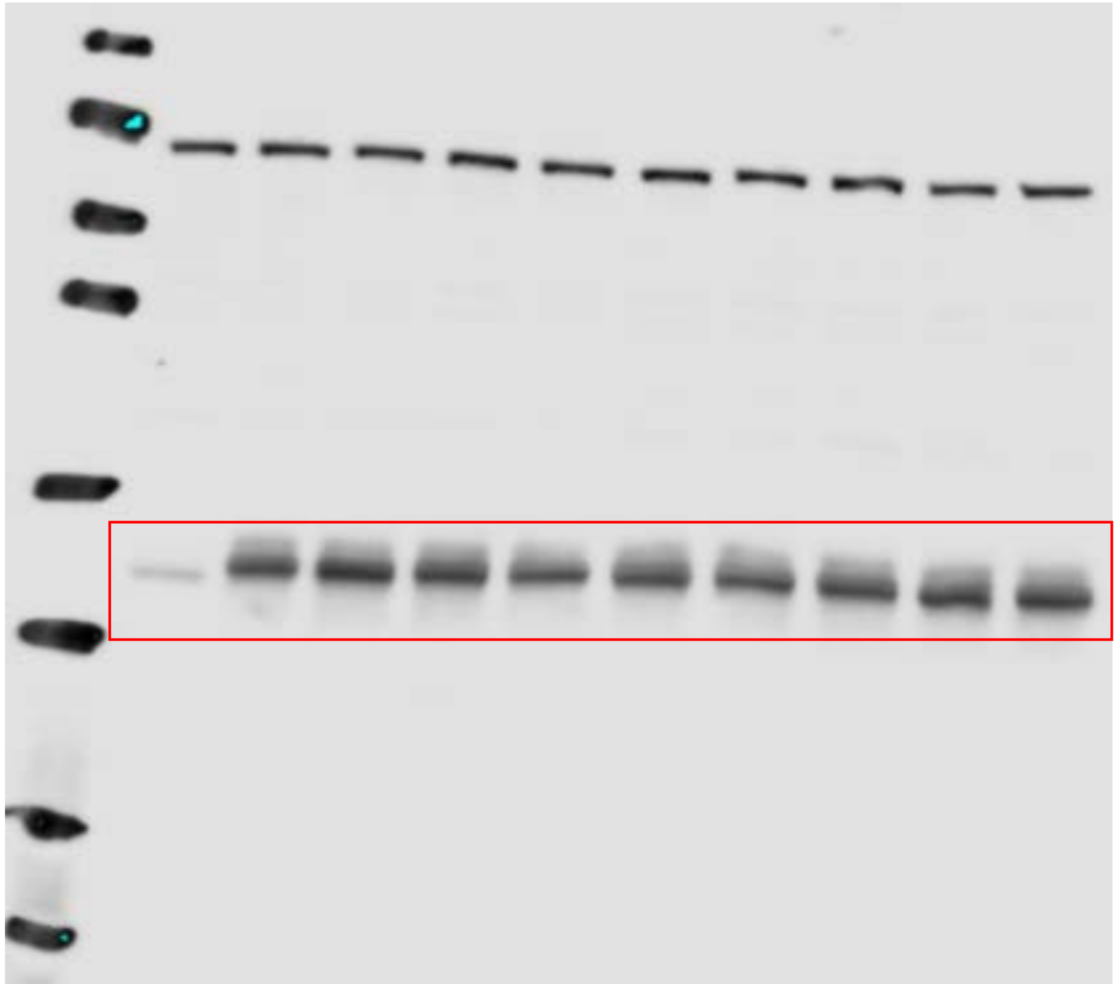

Figure 4G- Nlk (LS)

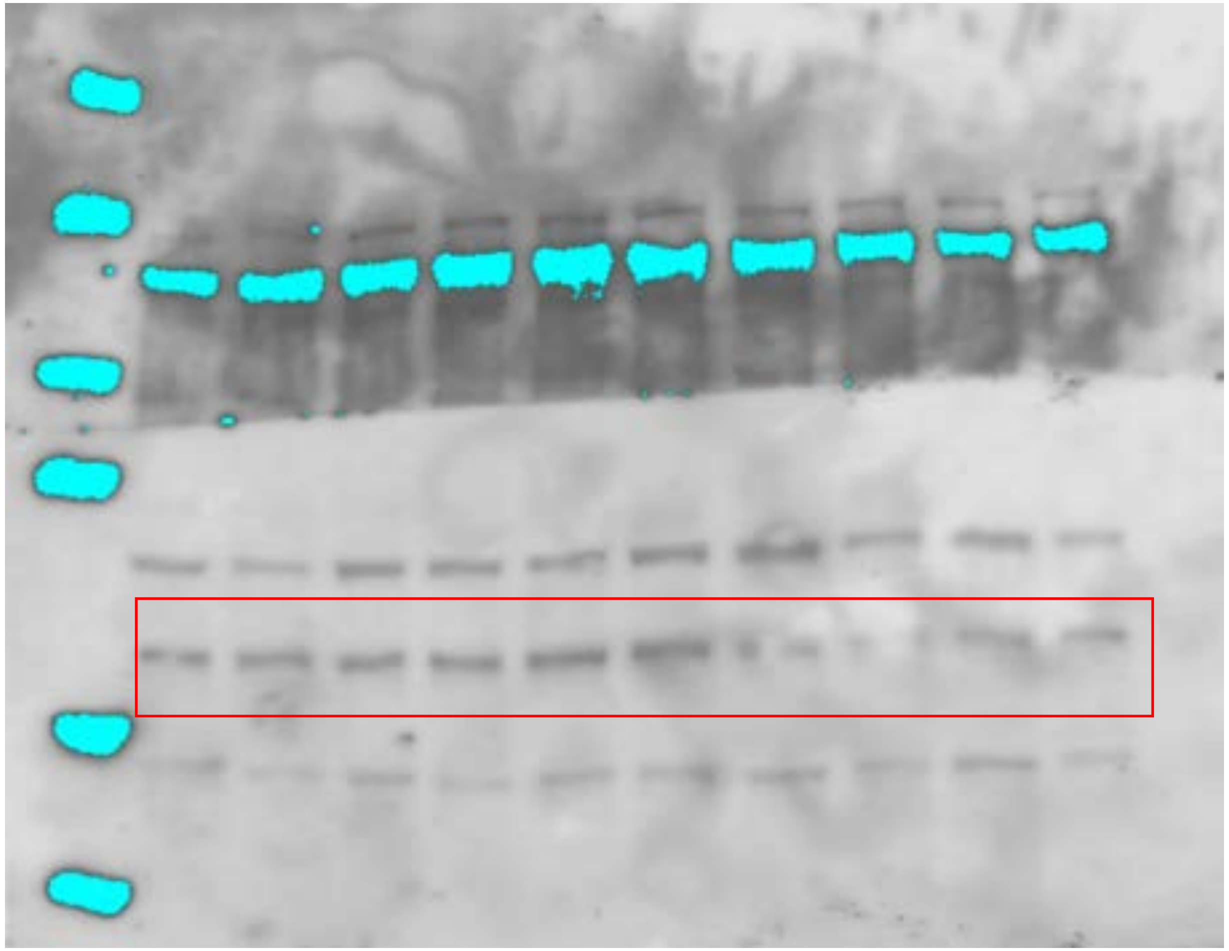

Figure 4G- Flag (LS)

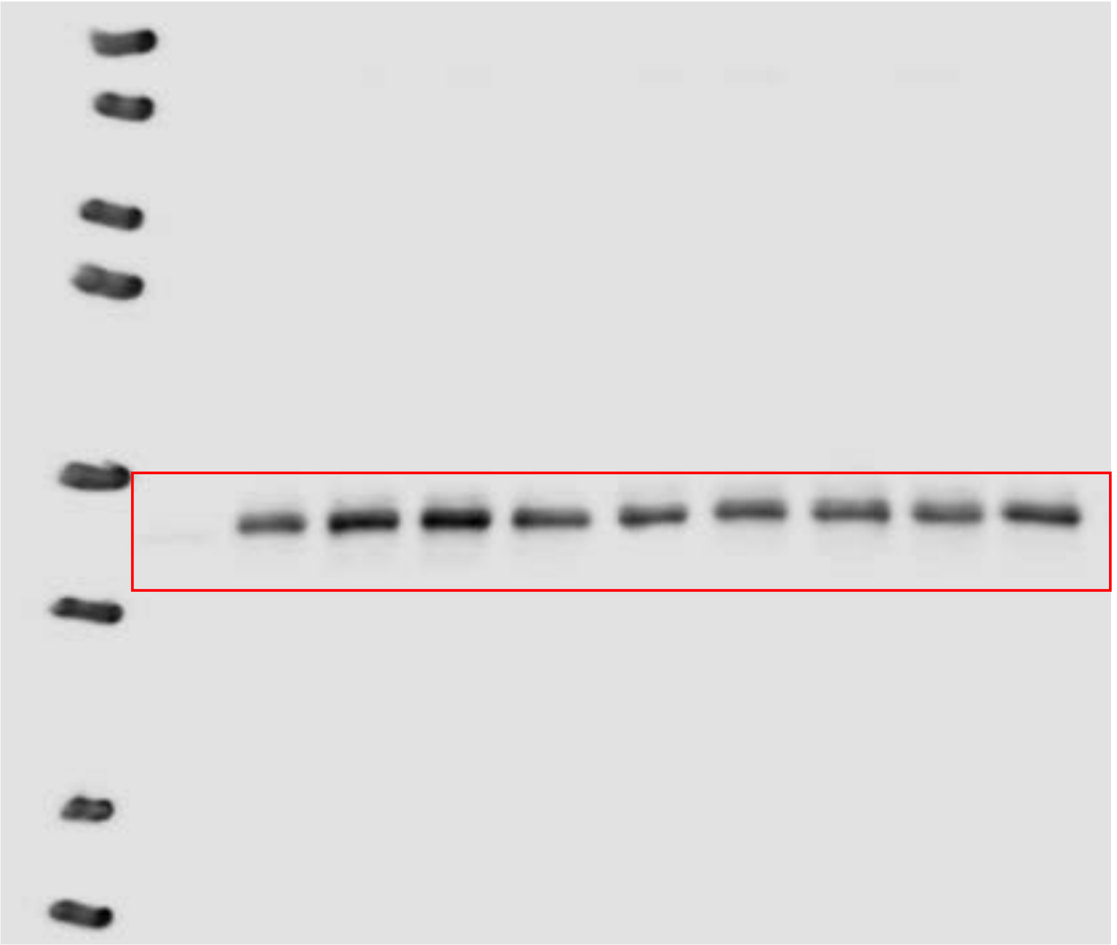

Figure 4G- TDP-43 (HS)

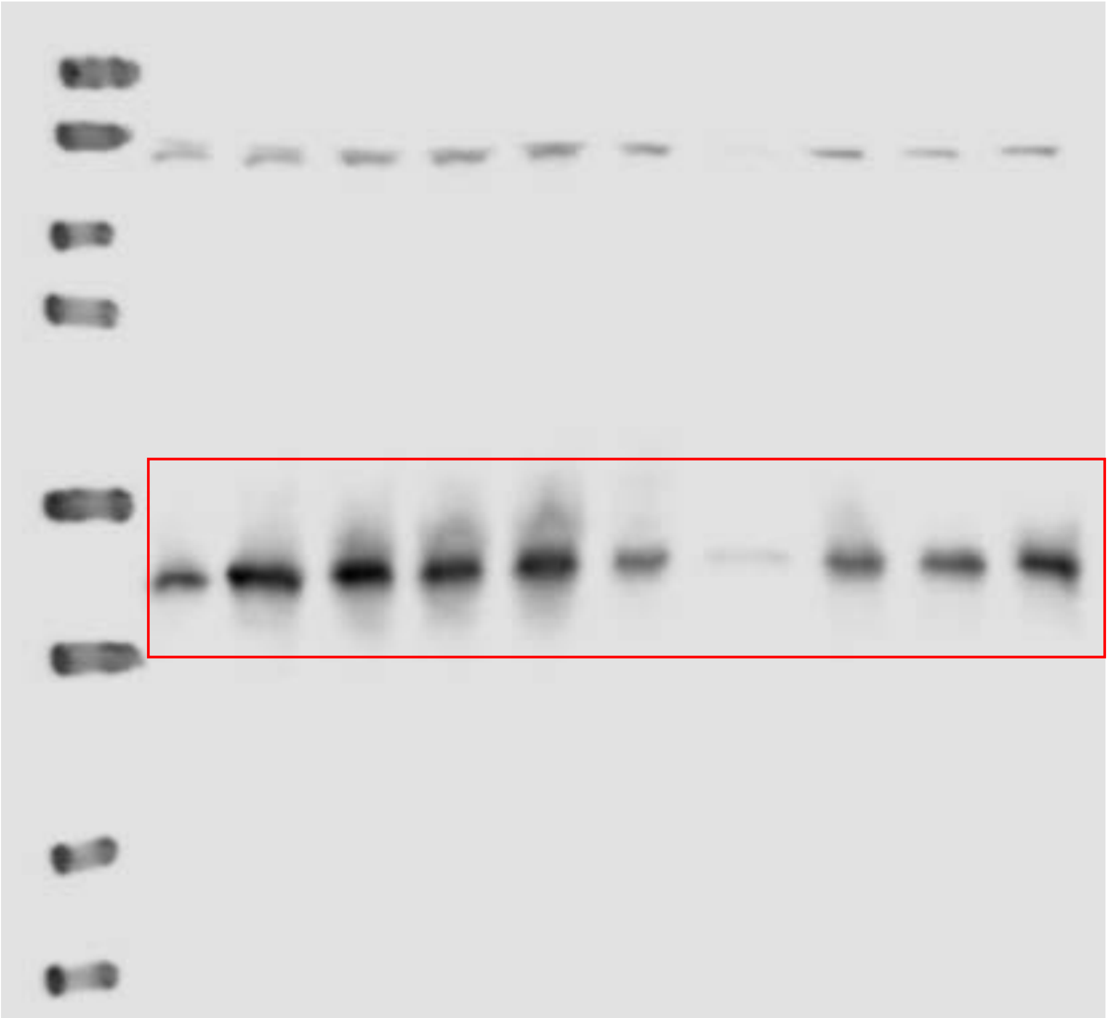

Figure 4G- Flag (HS)

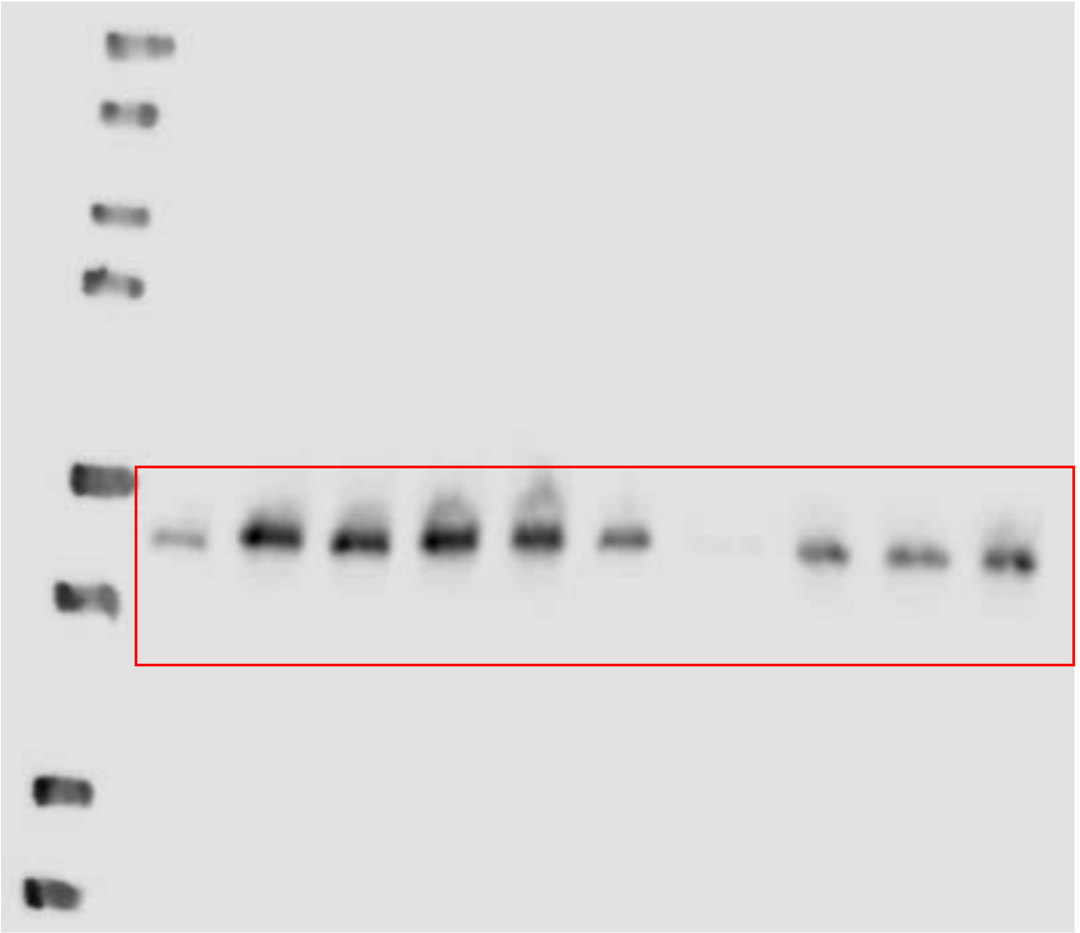

Figure 4G- TDP-43 (SK)

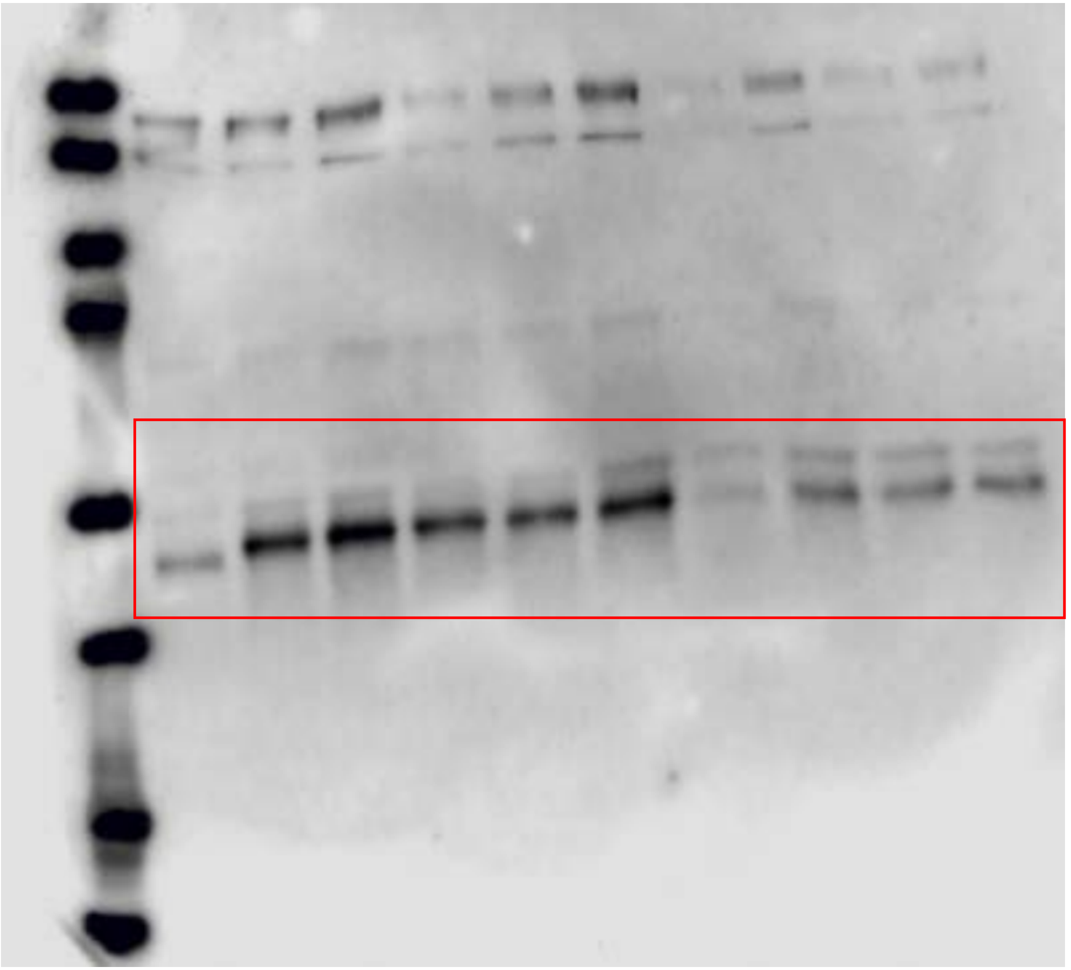

Figure 4G- Flag (SK)

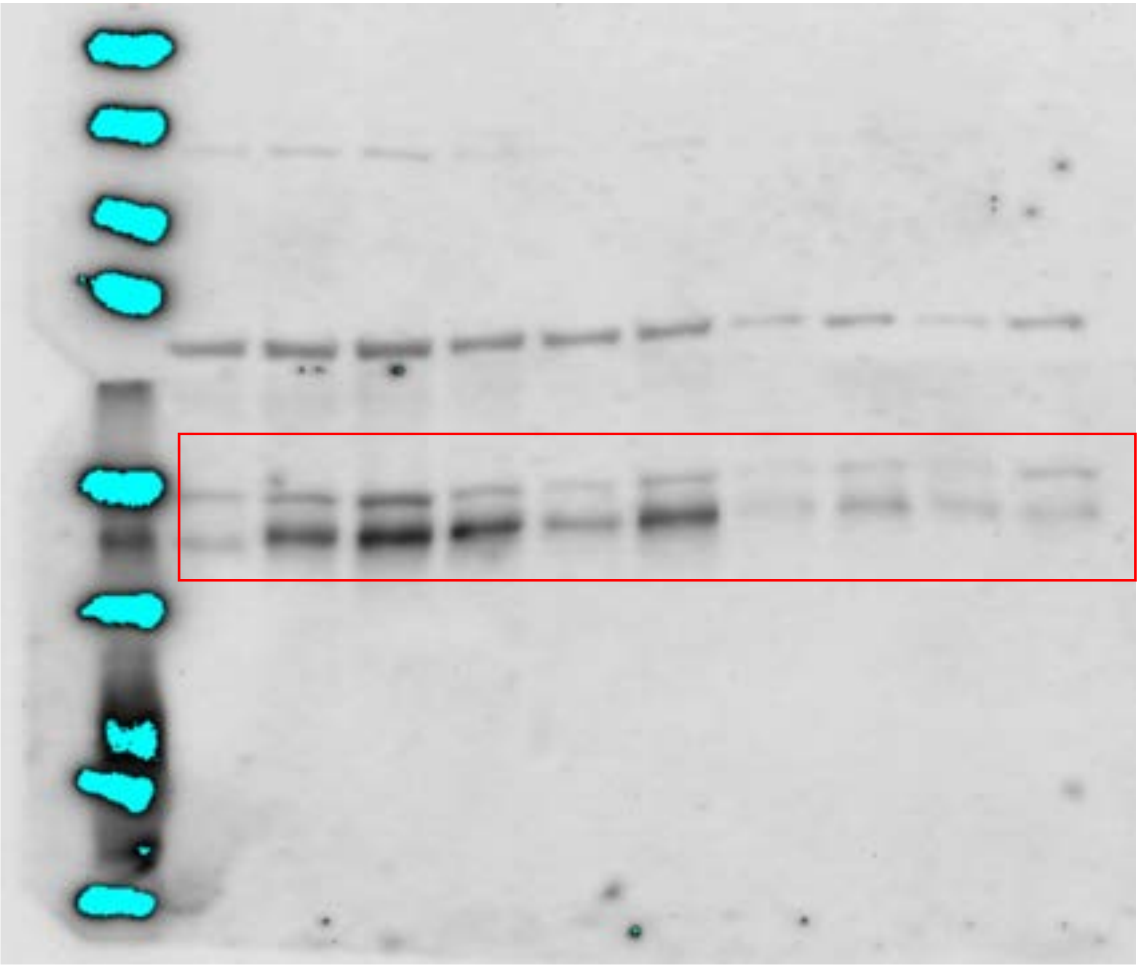

Figure 4G- TDP-43 (UR)

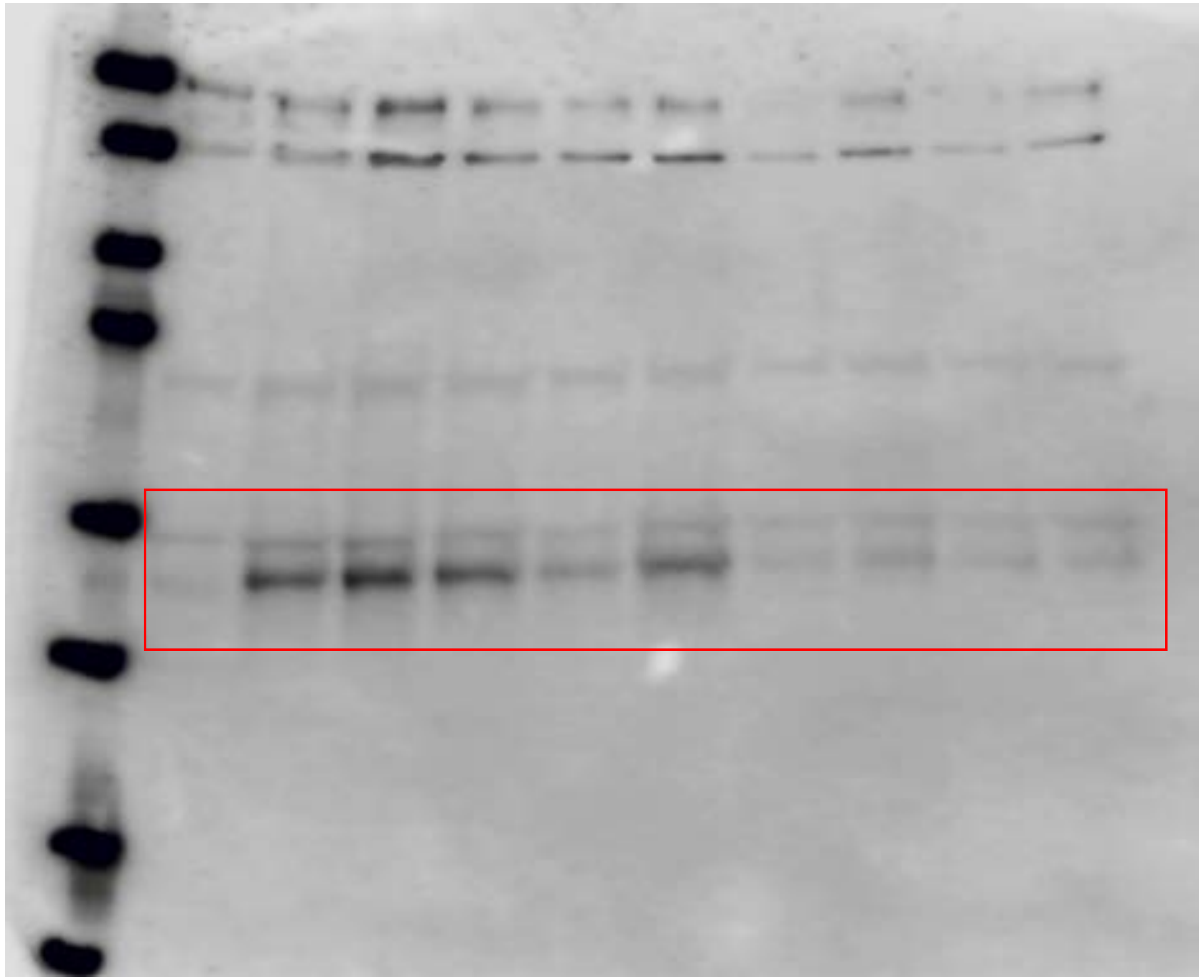

Figure 6I- Vinculin (LS)

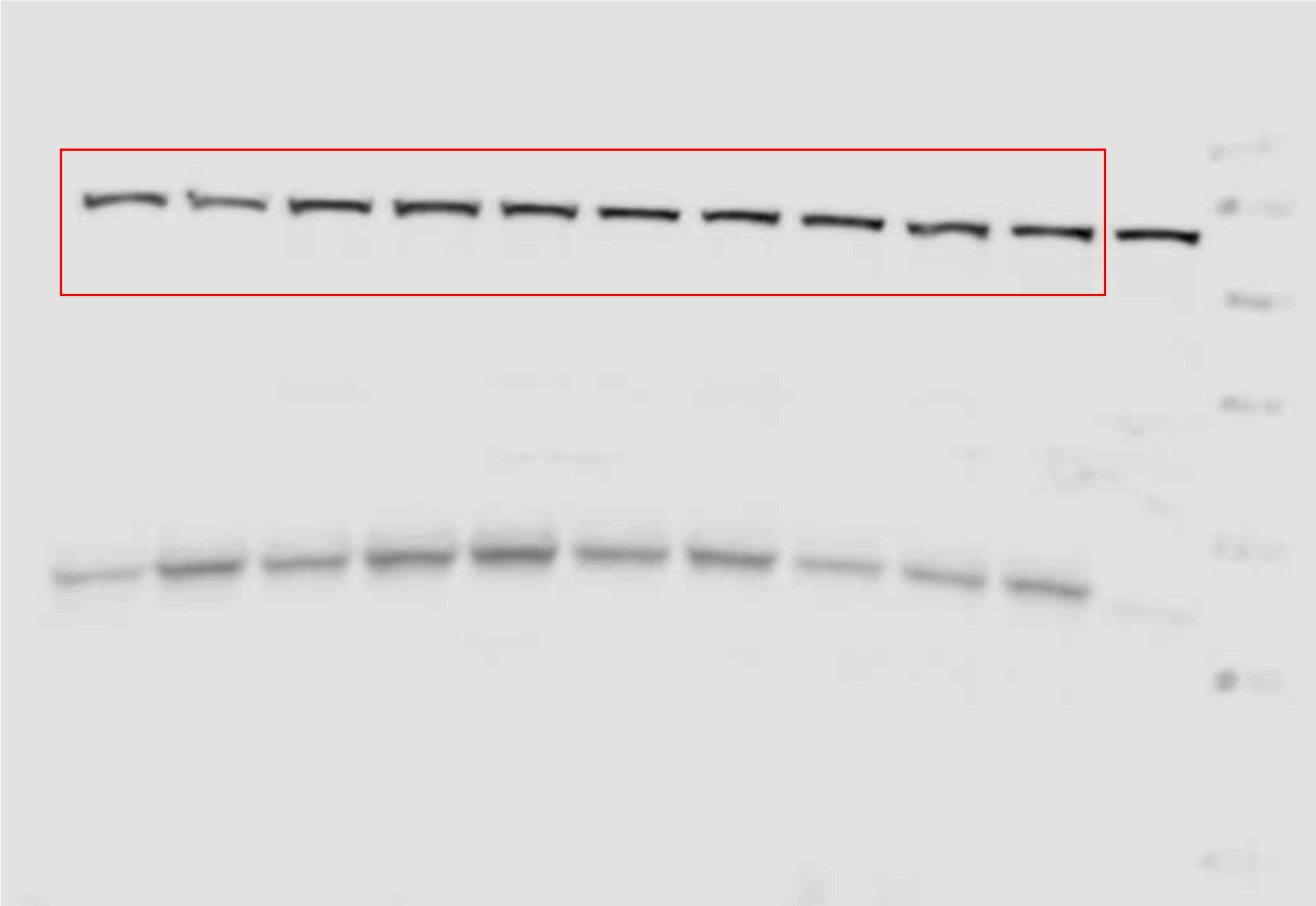

Figure 6I- TDP-43 (LS)

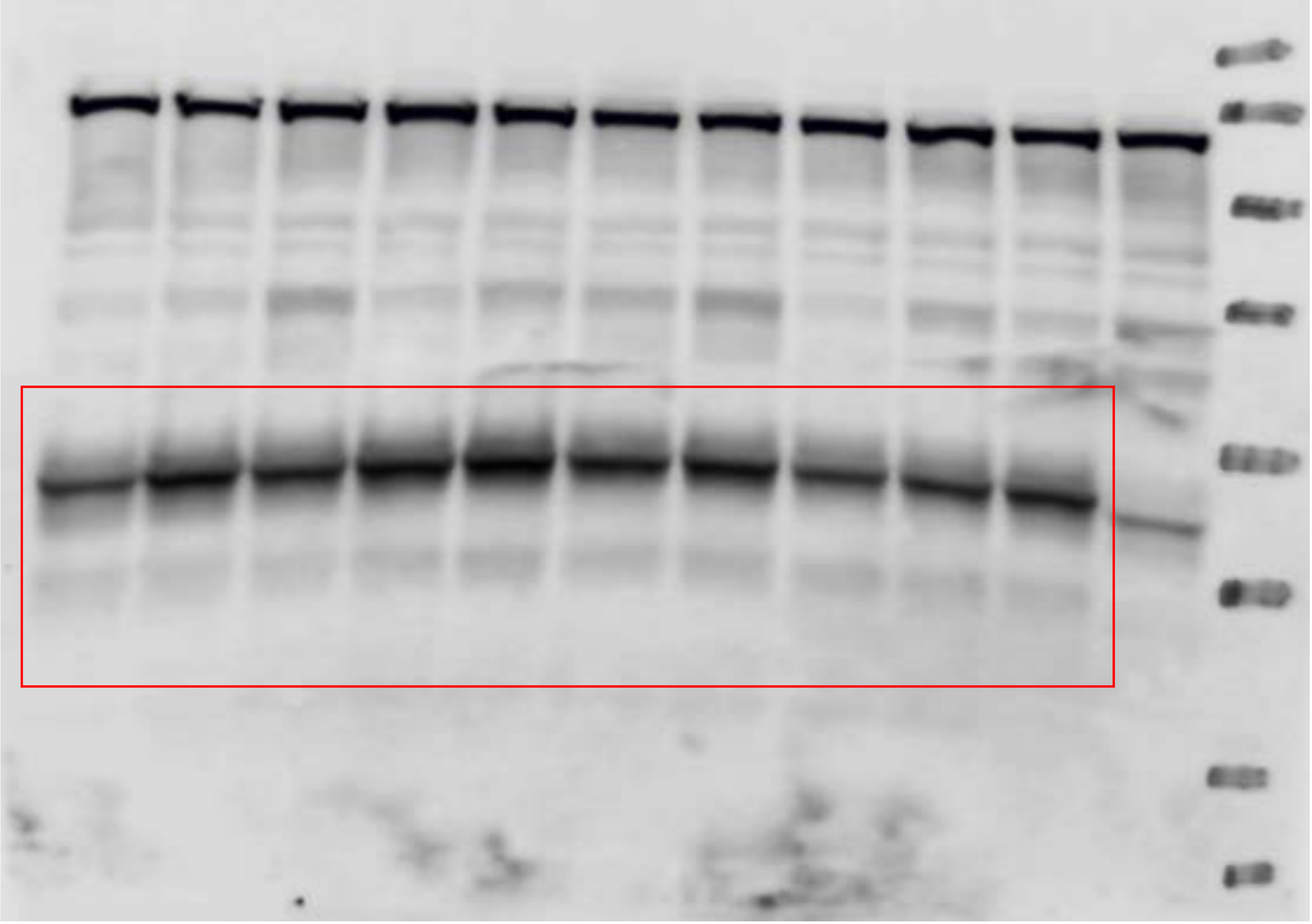

Figure 6I- Flag (LS)

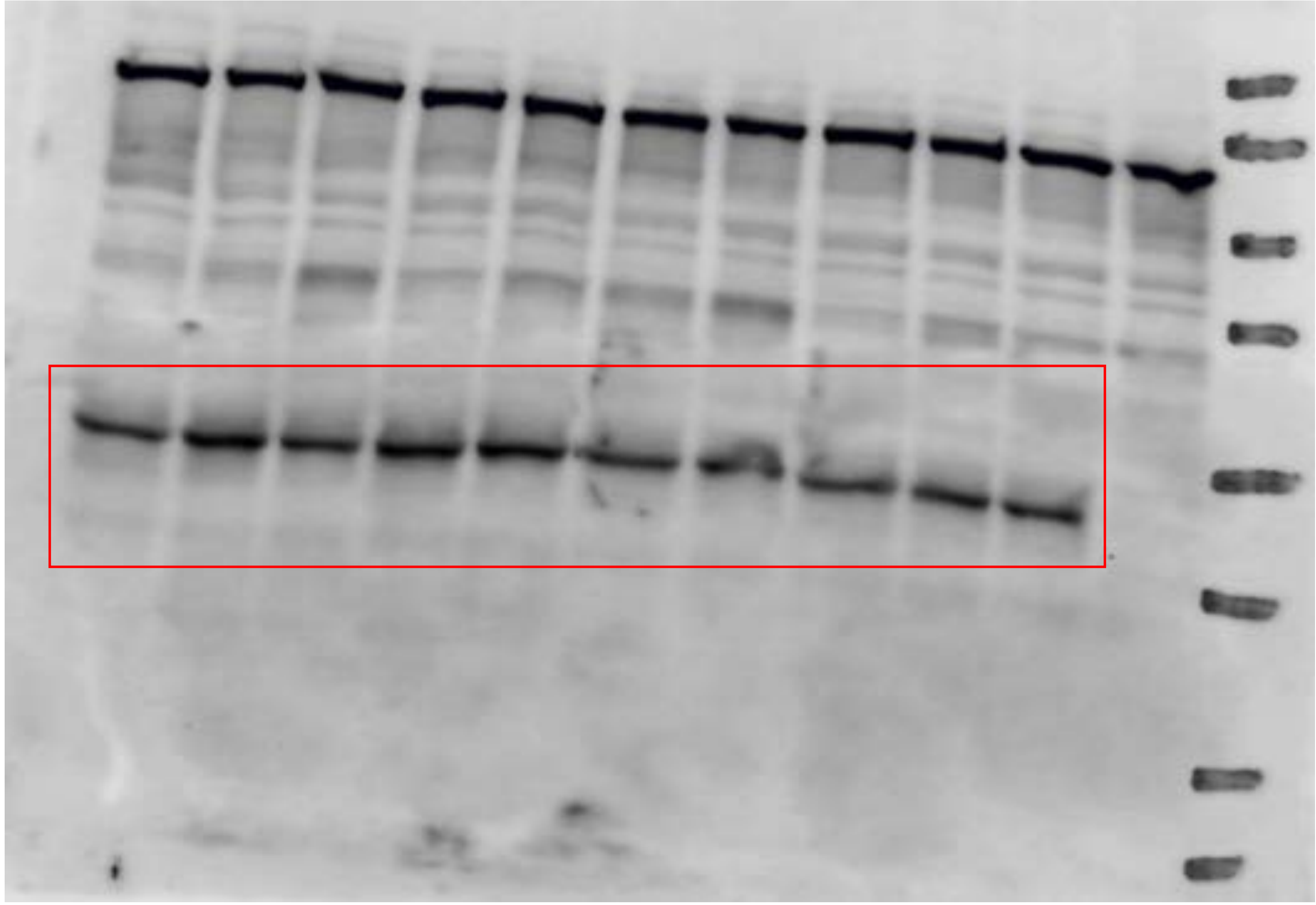

Figure 6I- TDP-43 (SK)

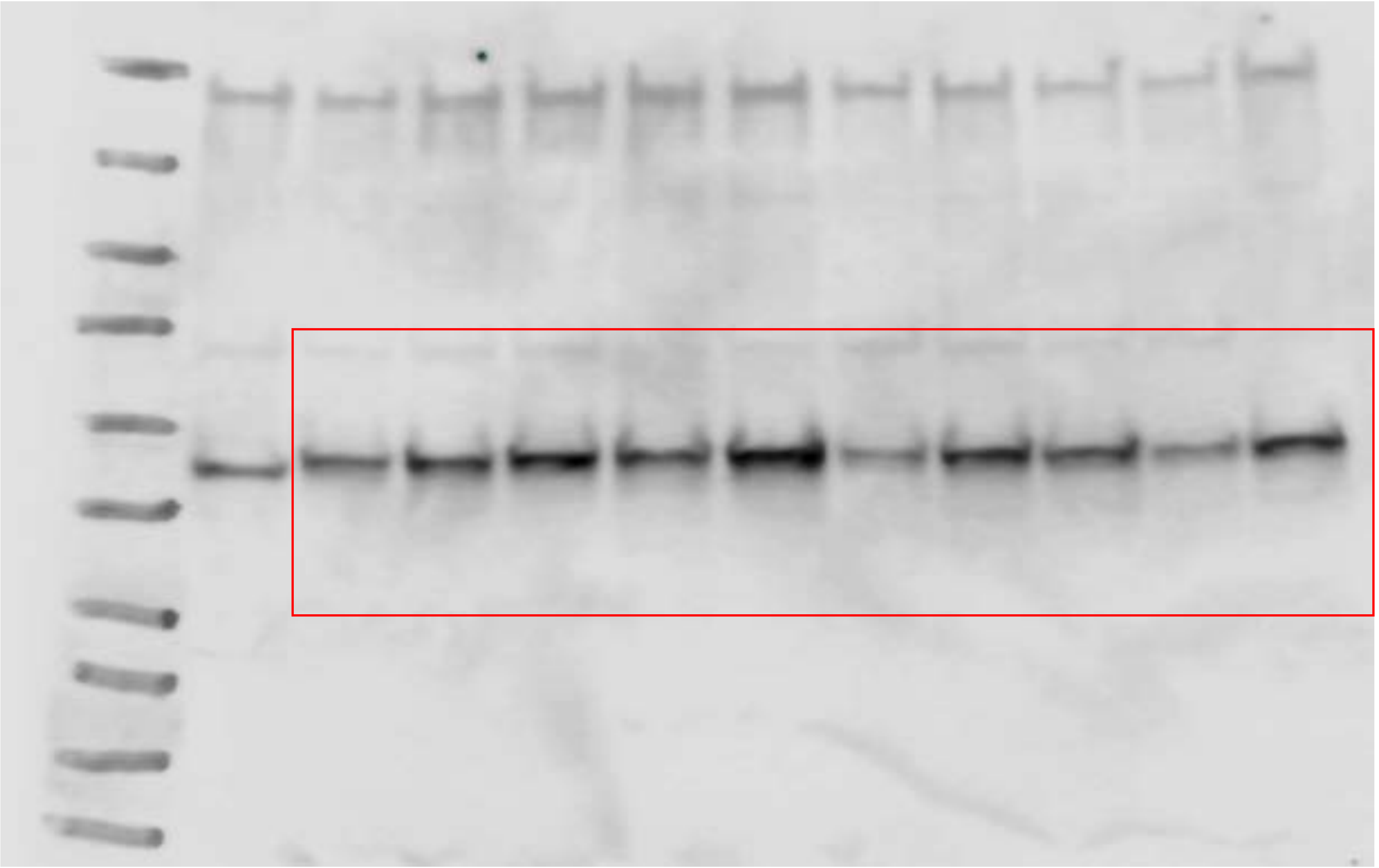

Figure 6I- TDP-43 (Urea+SDS)

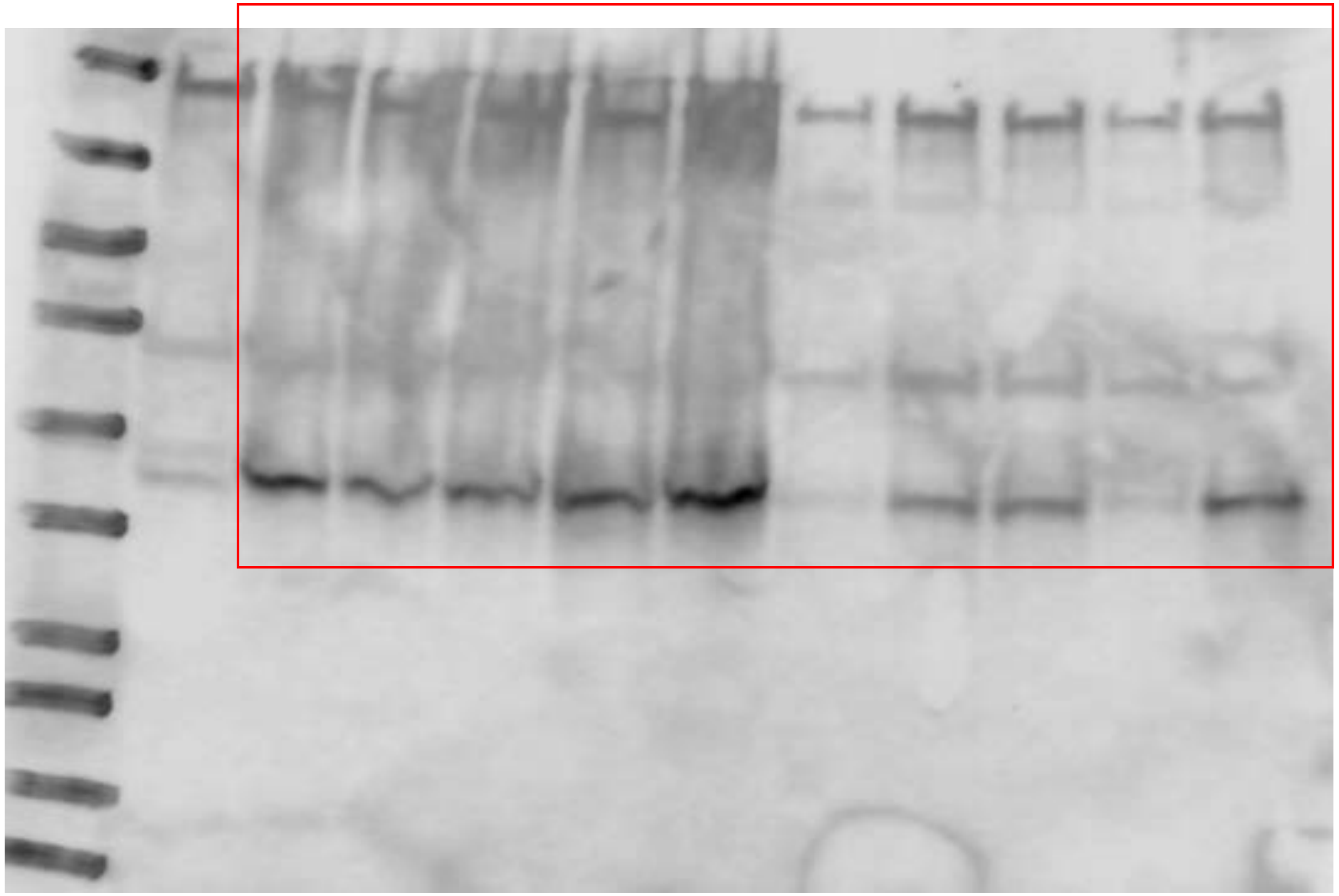

Figure S1- Nlk

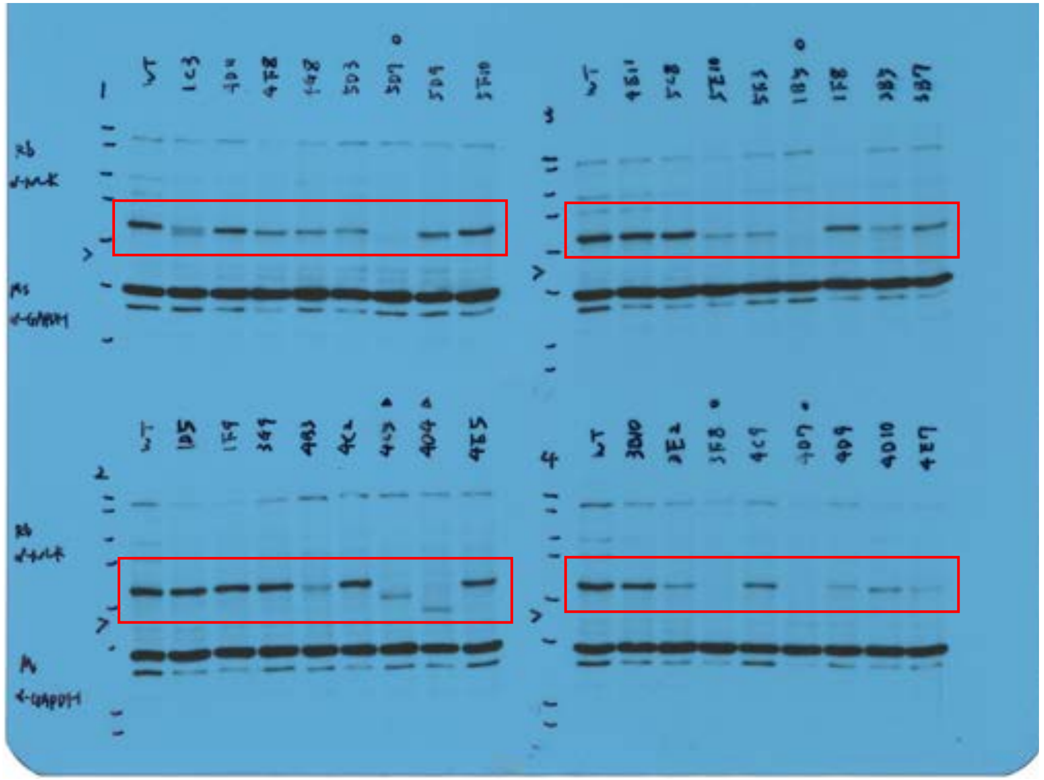

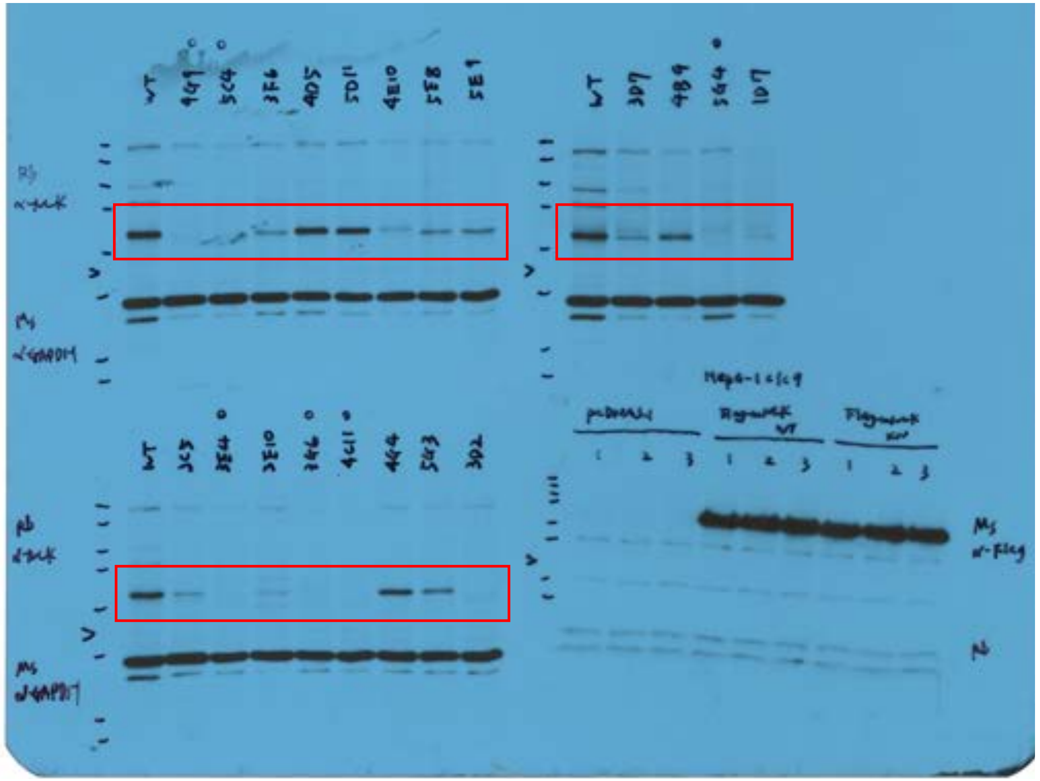

Figure S1- Gapdh

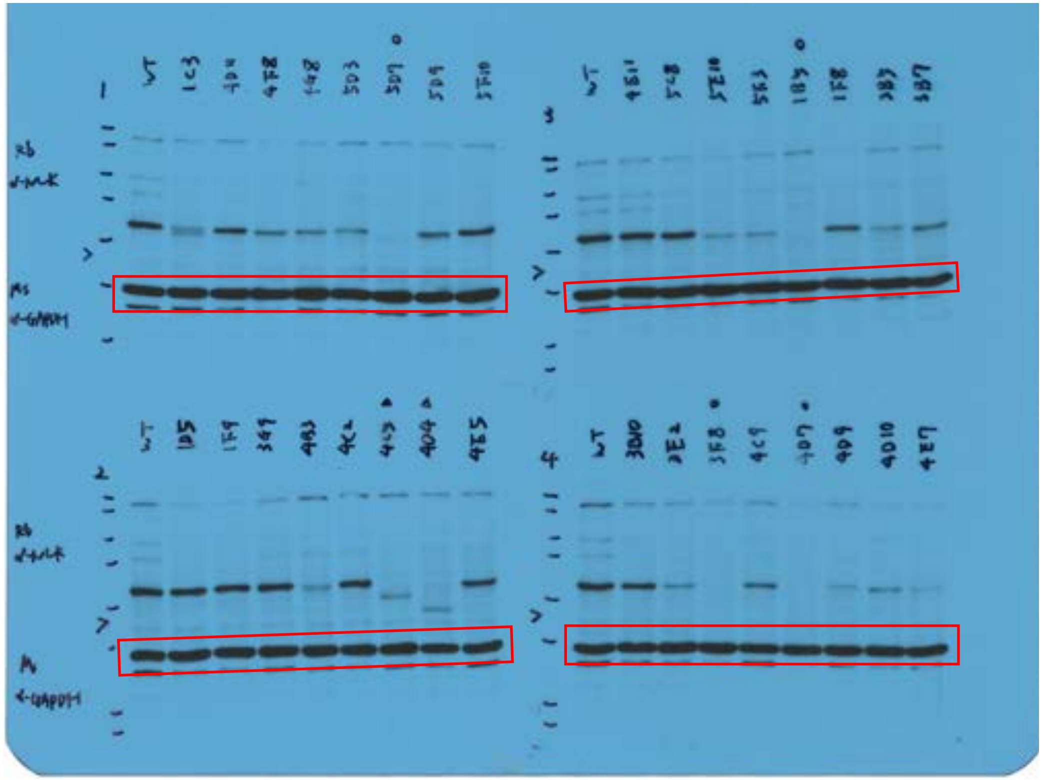

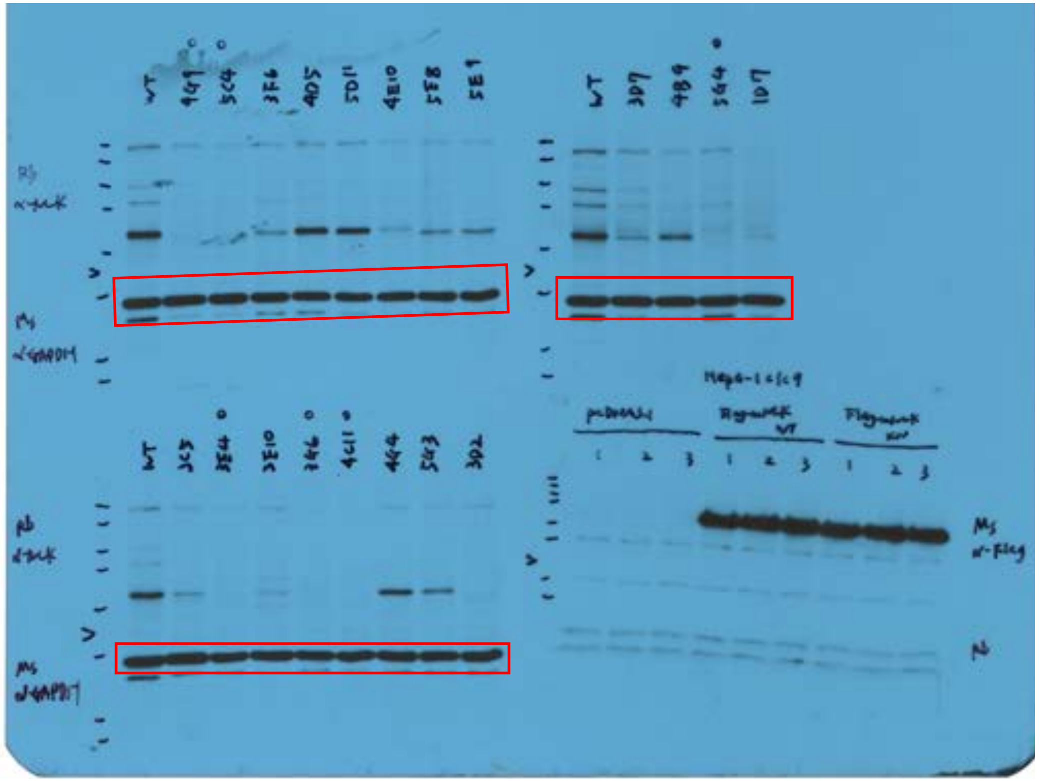

Figure S3I- p62

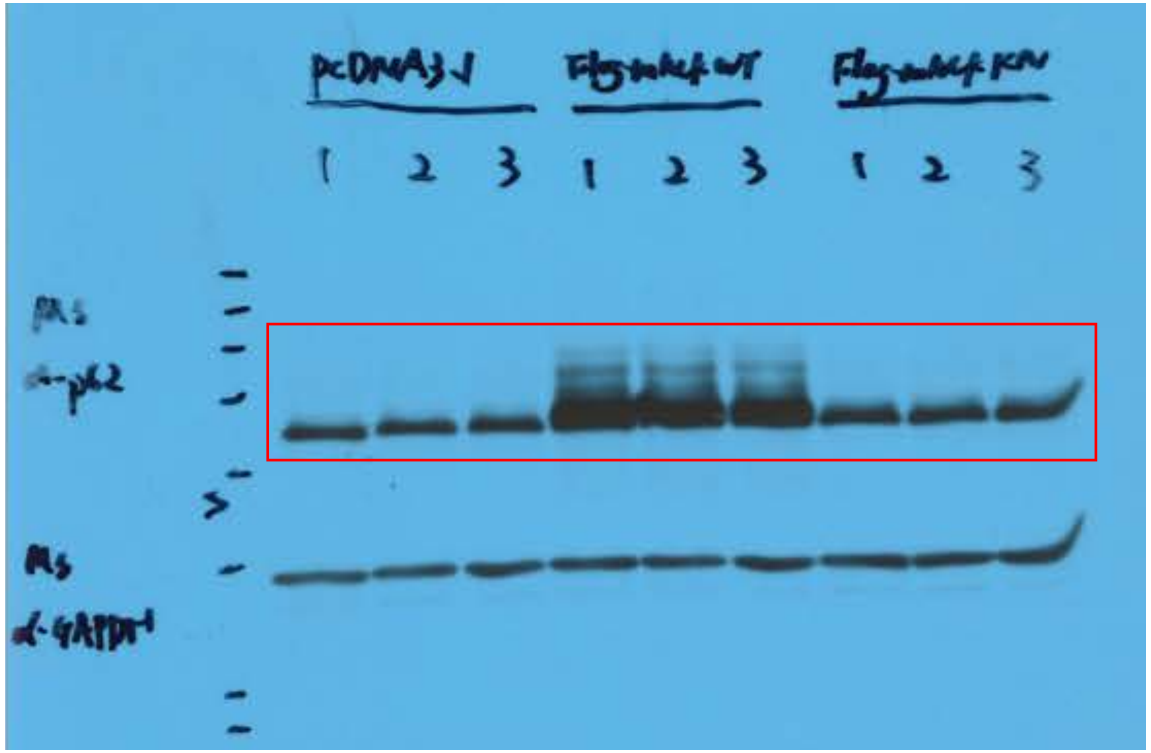

Figure S3I- LC3

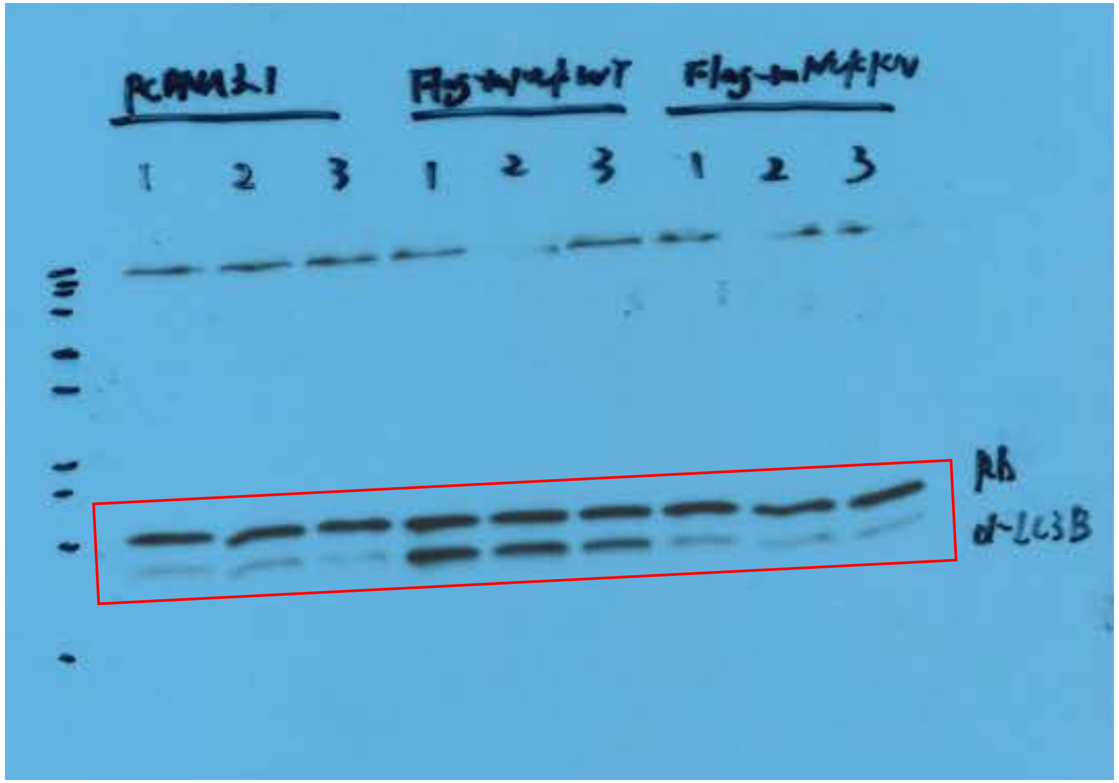

Figure S3I- Flag (Nlk)

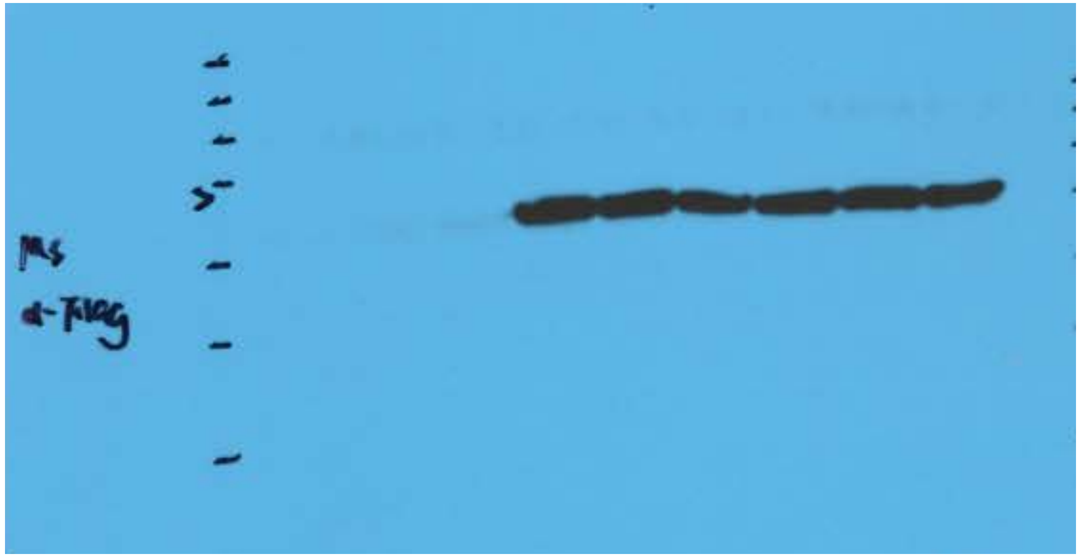

Figure S3I- Gapdh

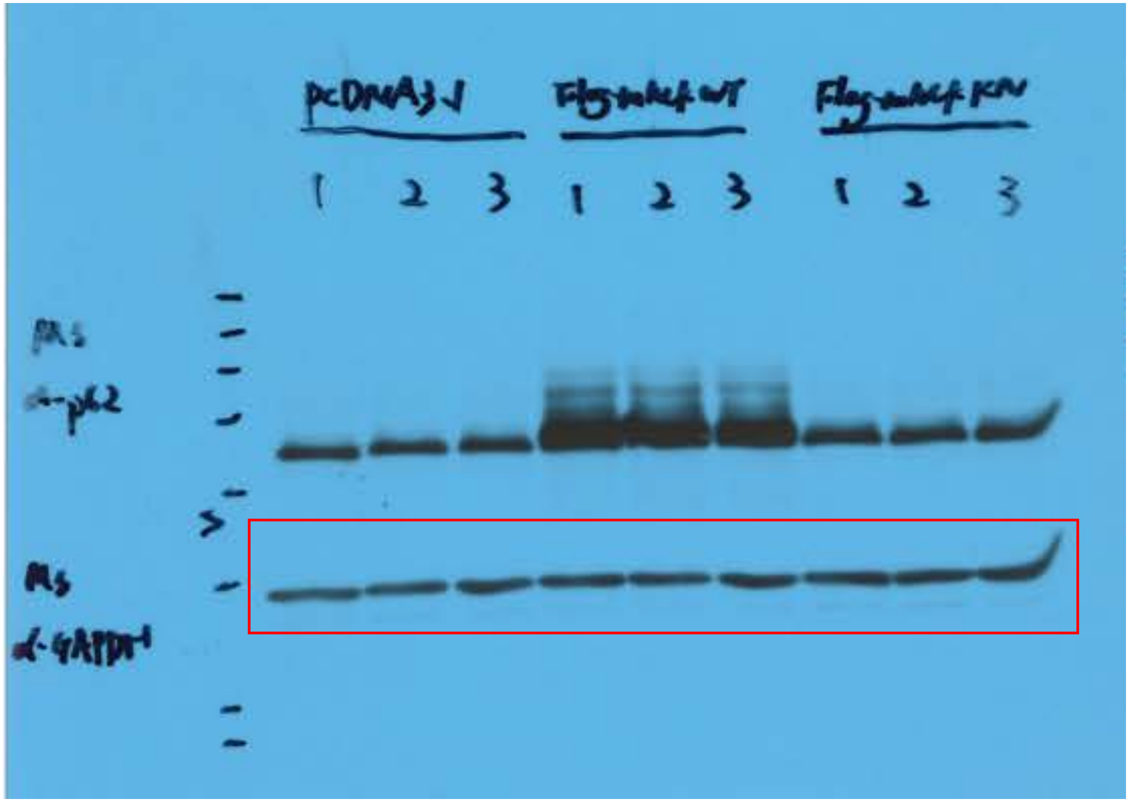

Figure S3J- GFP

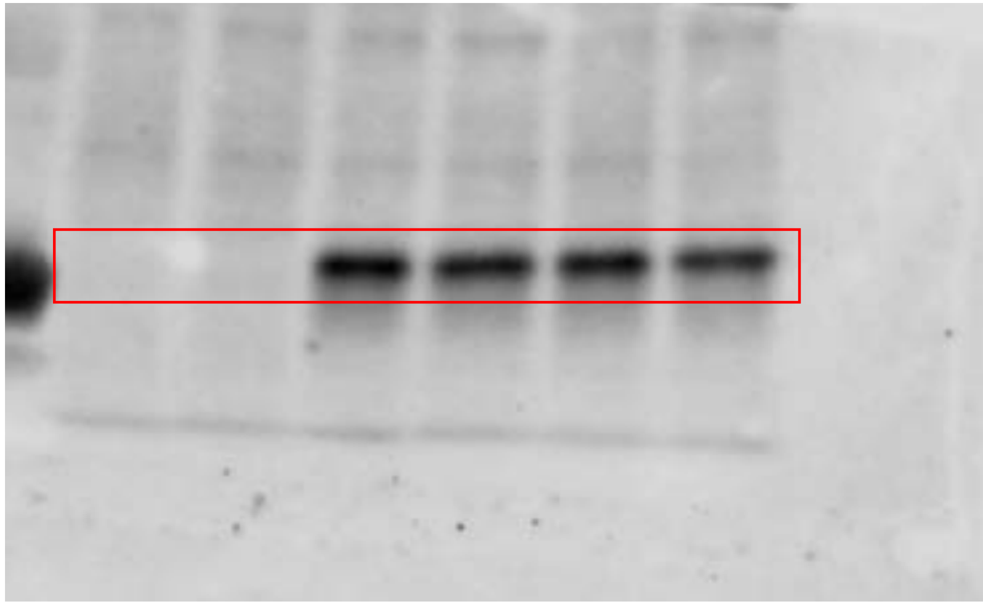

Figure S3J- Flag

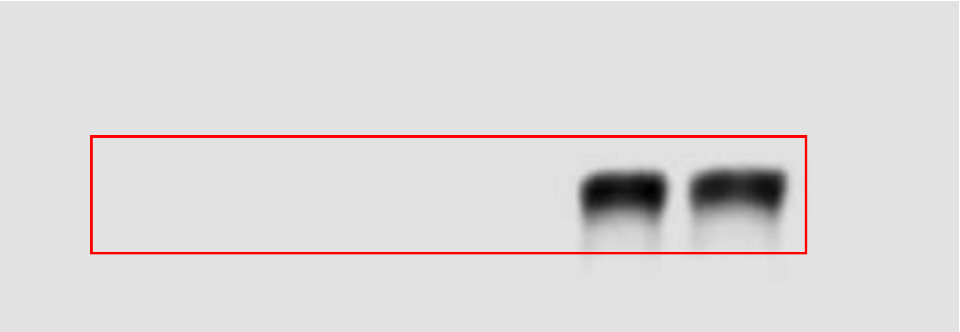

Figure S3J- Vinculin

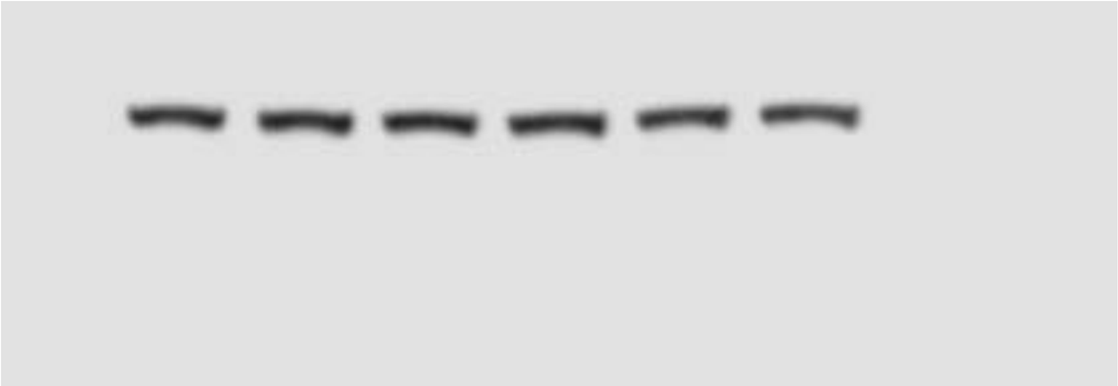

Figure S3K- Flag (Nlk)

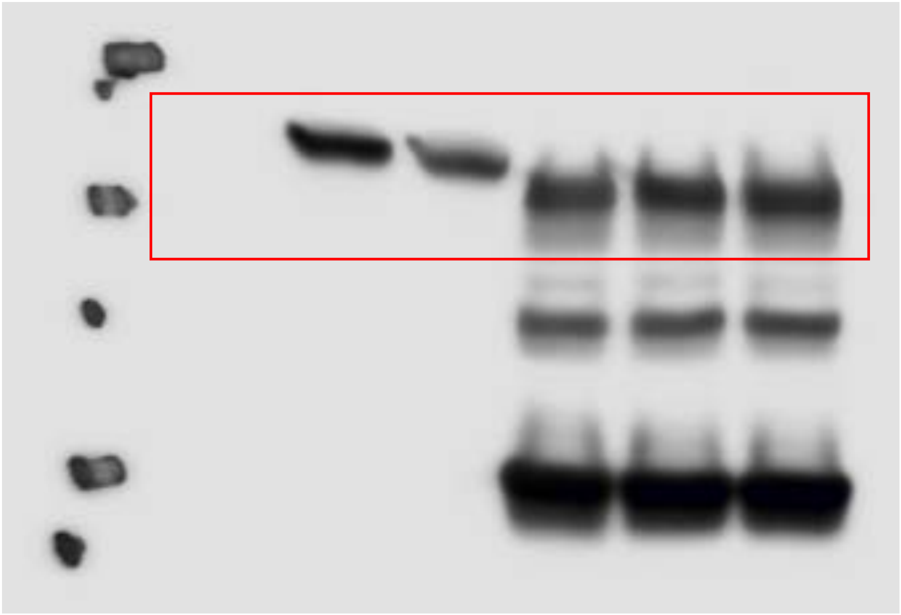

Figure S3K- HA

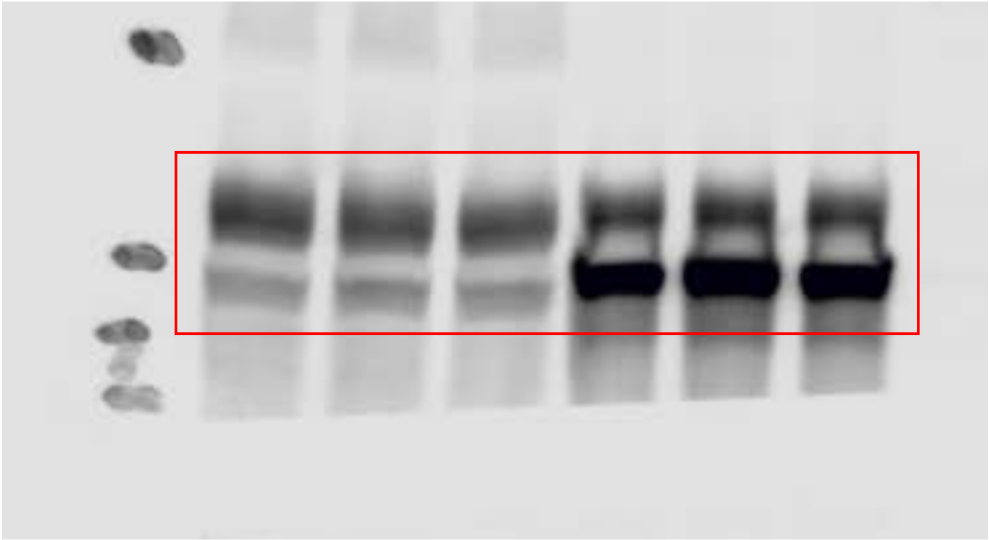

Figure S3K- Ub

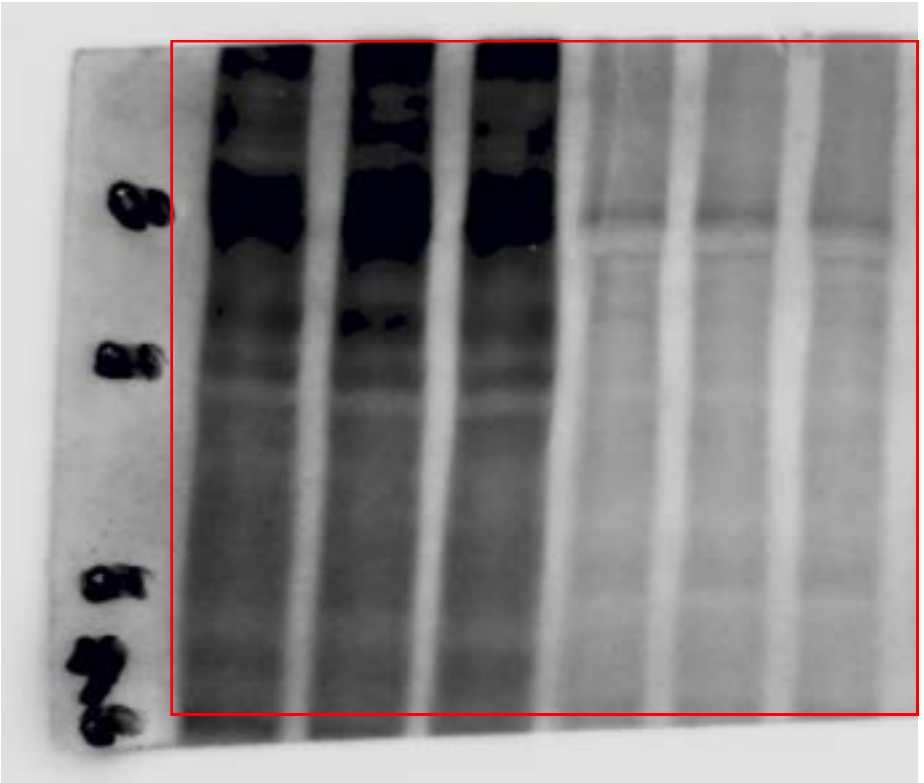

Figure S4A- NLK

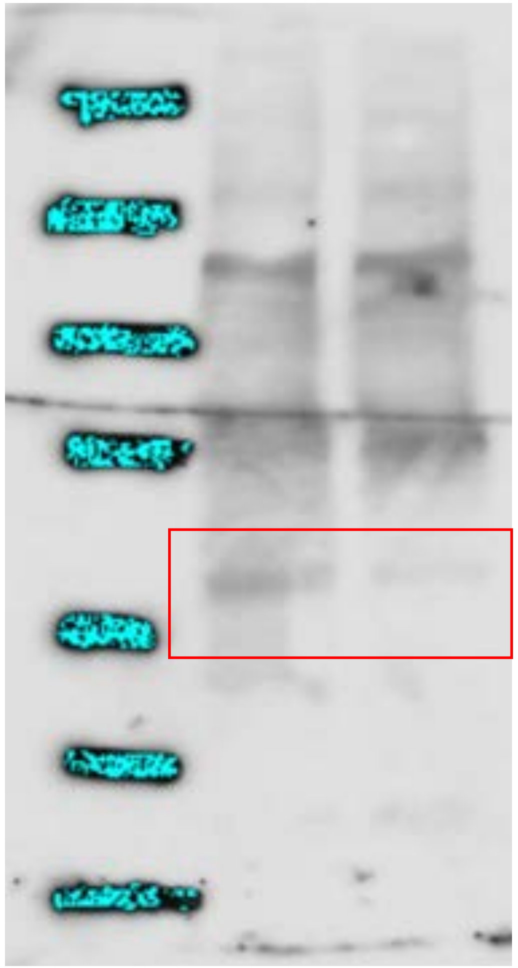

Figure S4A- Vinculin

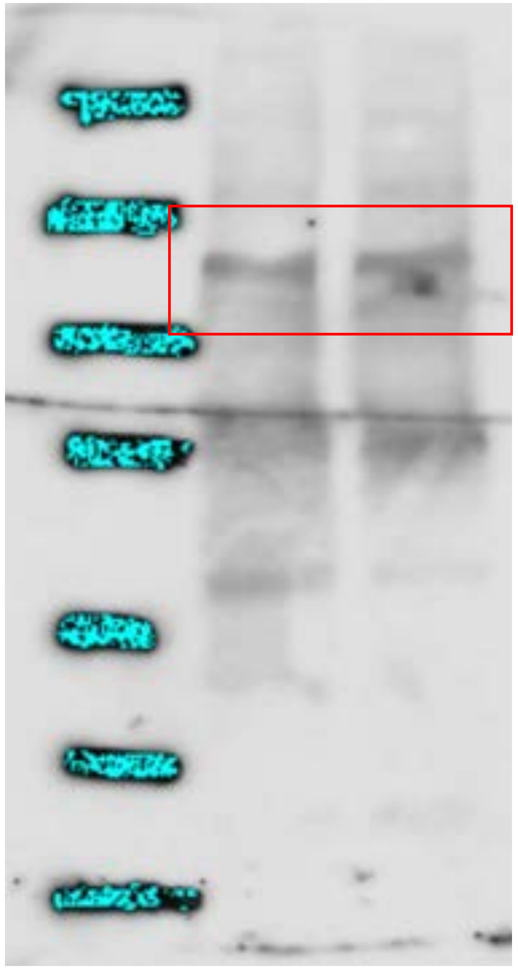

Figure S5A- p4E-BP1

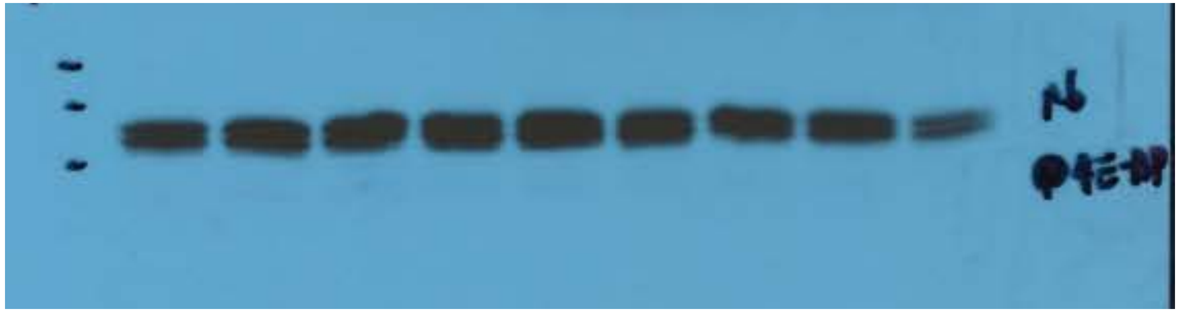

Figure S5A- 4E-BP1

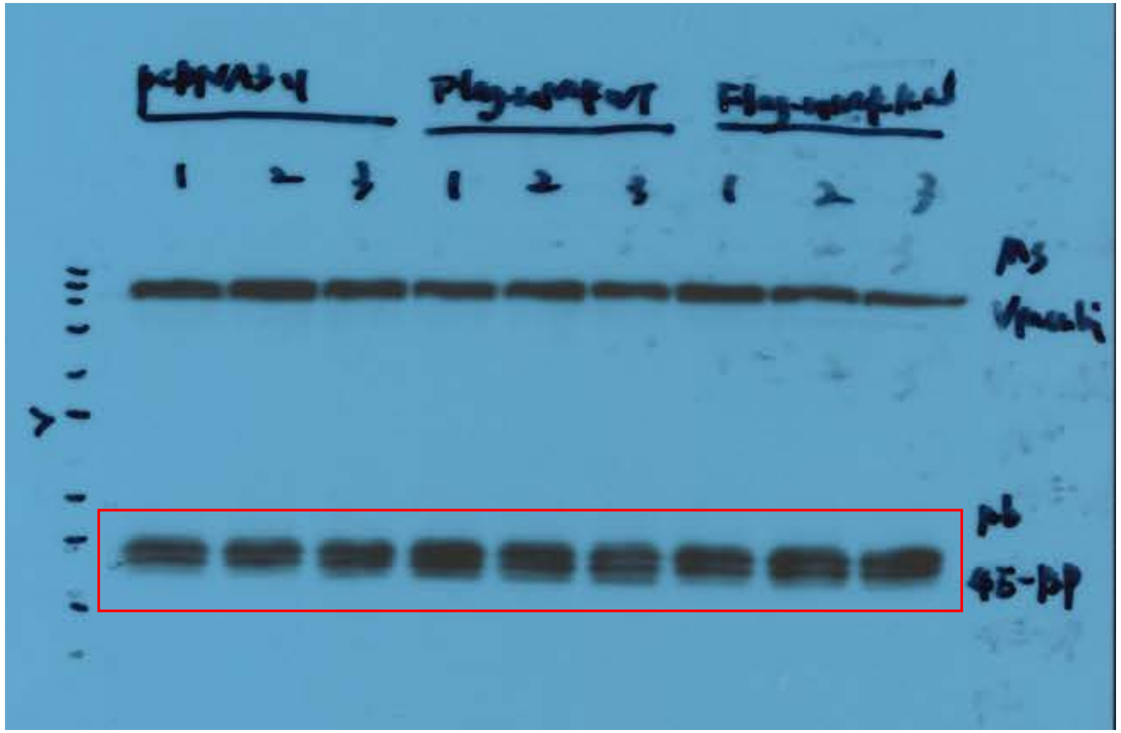

Figure S5A- pS6

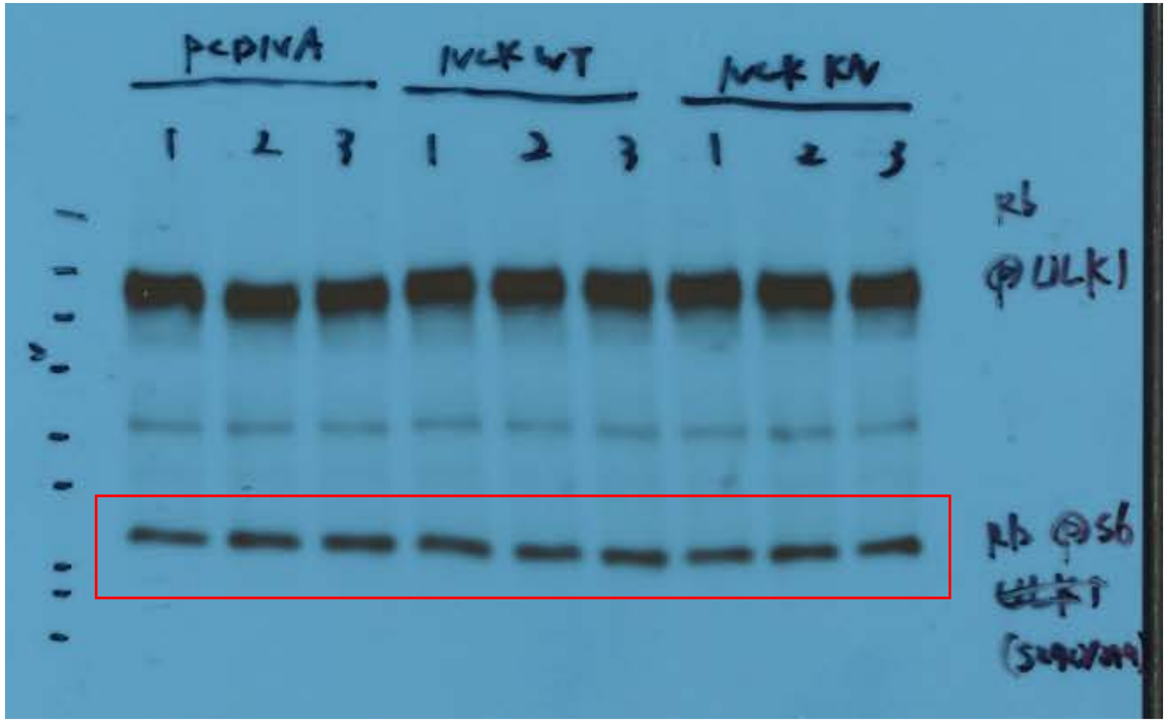

Figure S5A- S6

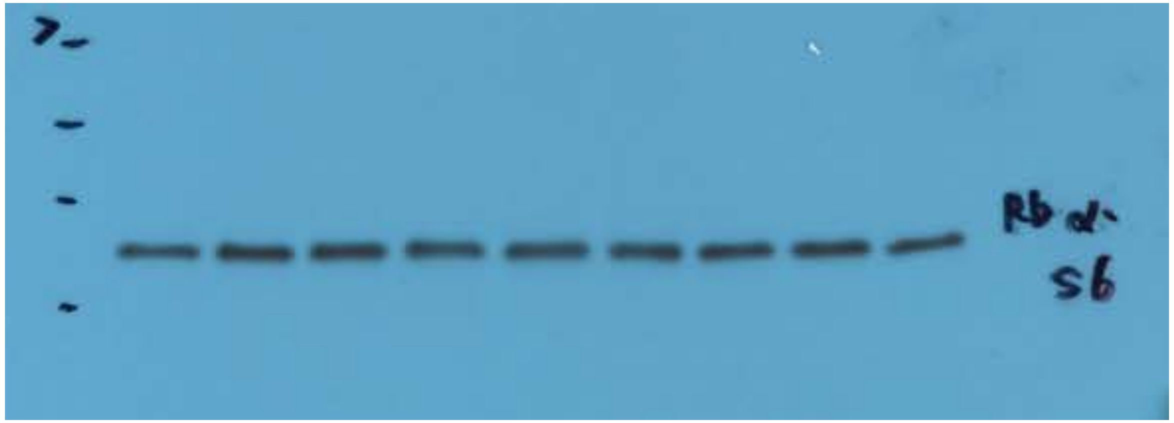

Figure S5A- pULK1

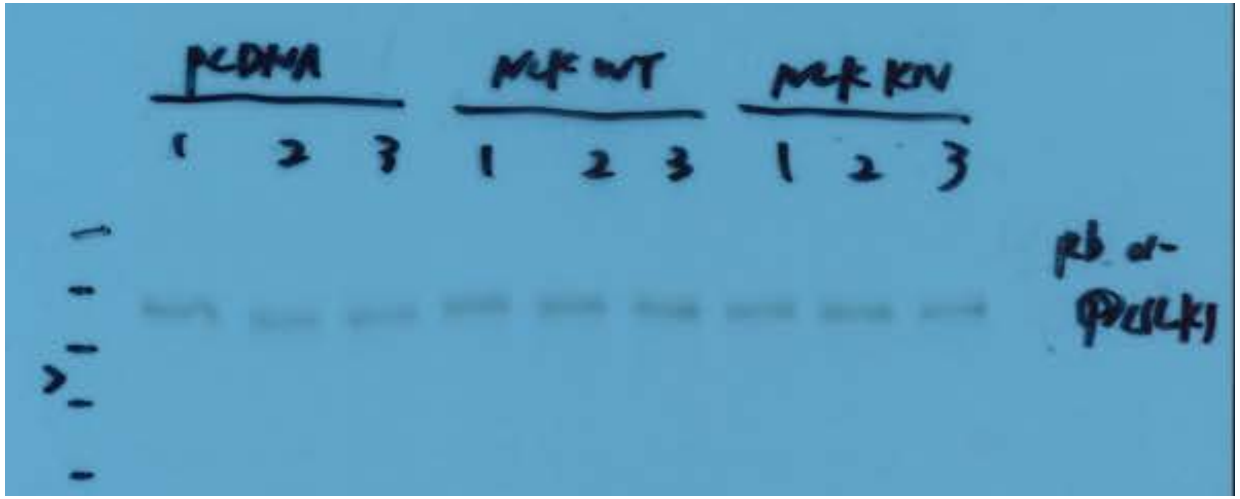

Figure S5A- ULK1

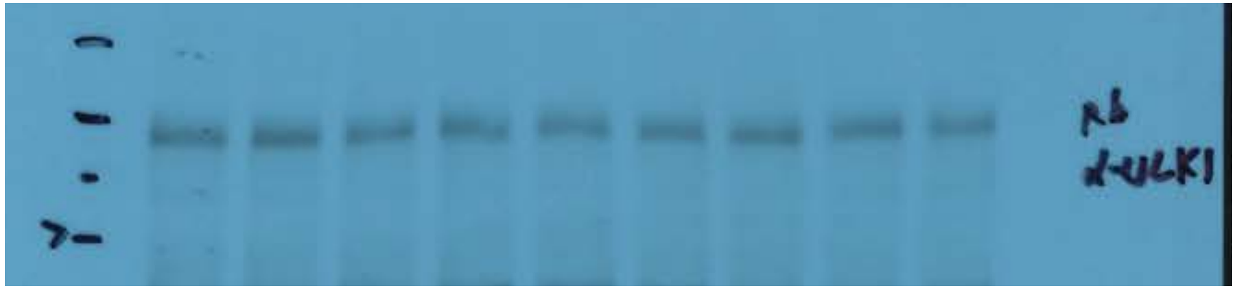

Figure S5A- Flag (Nlk)

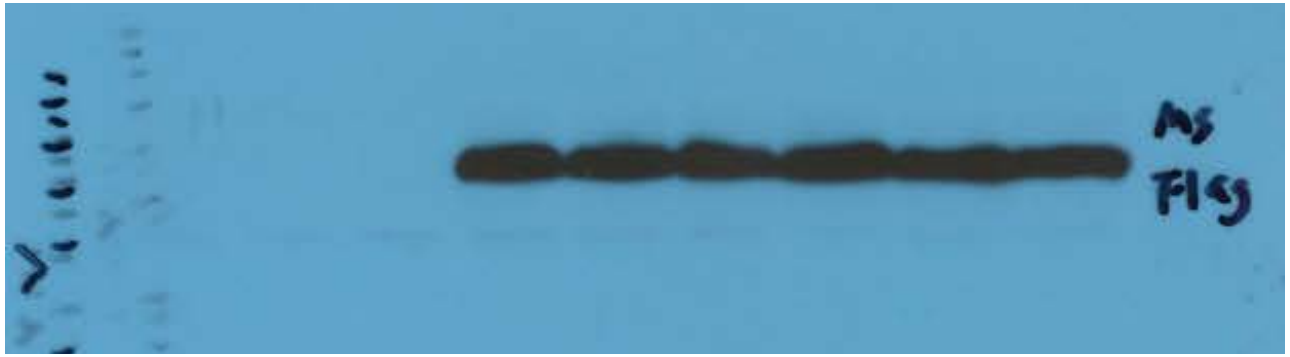

Figure S5A- Vinculin

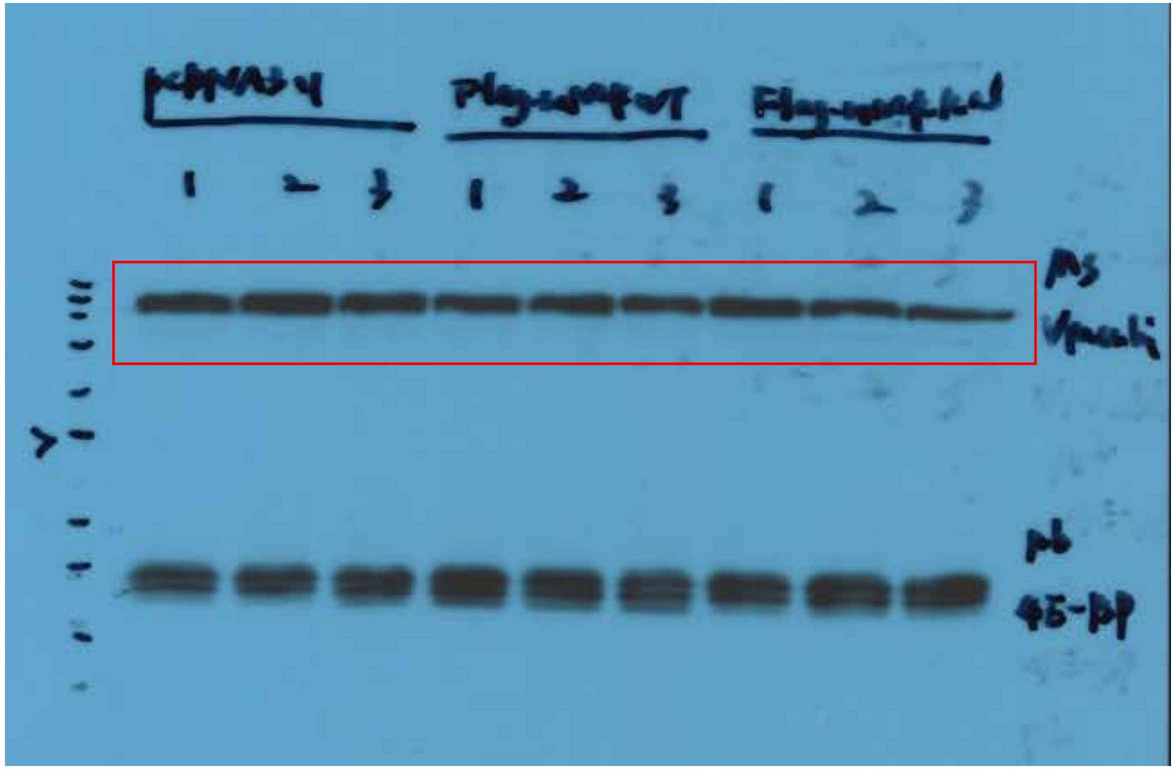

Figure S5C- pS6

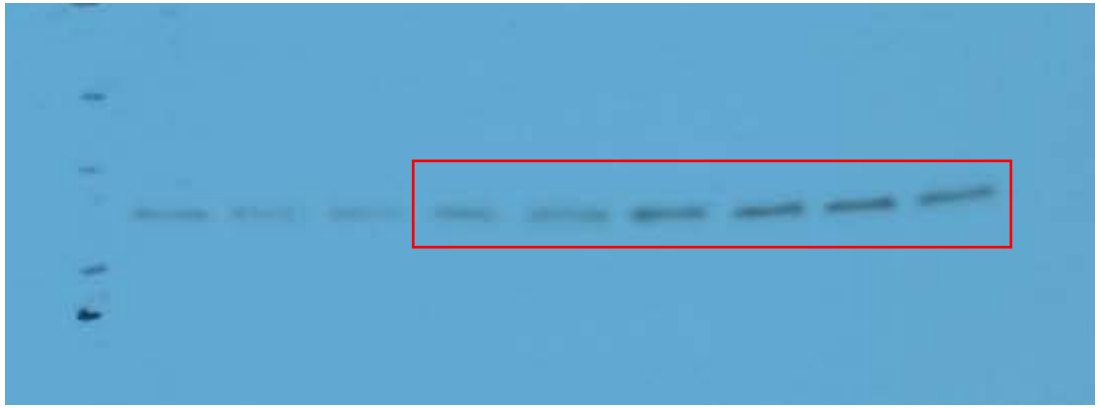

Figure S5C- S6

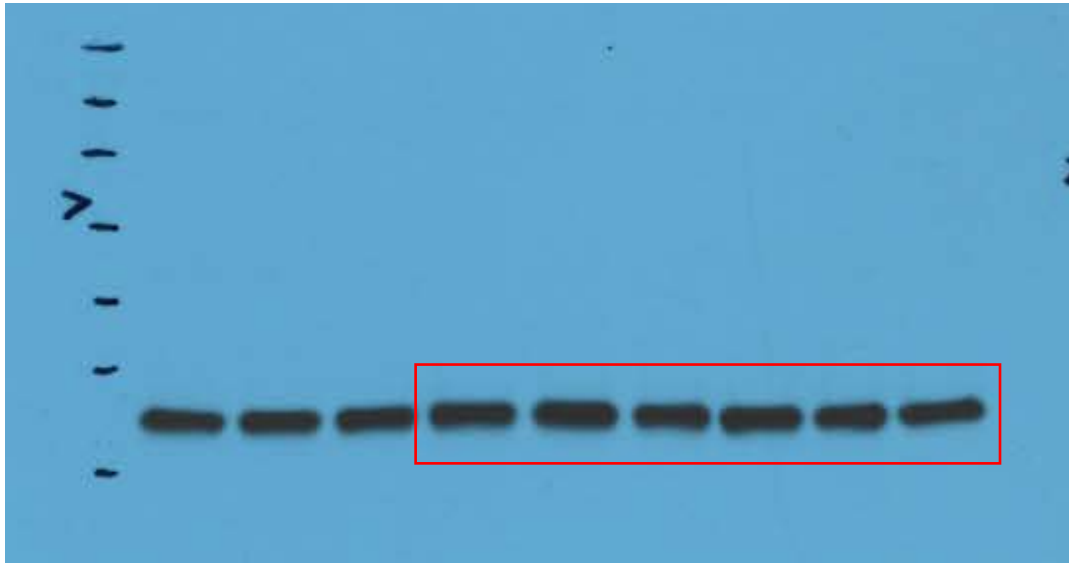

Figure S5C- pULK1

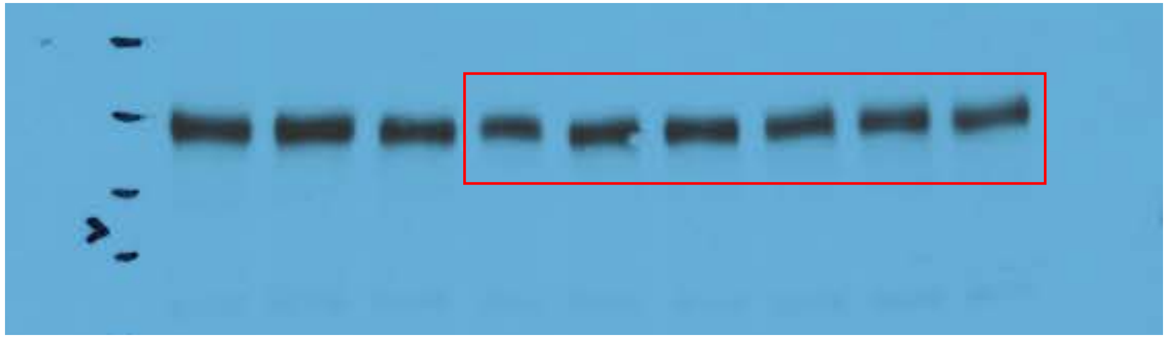

Figure S5C- ULK1

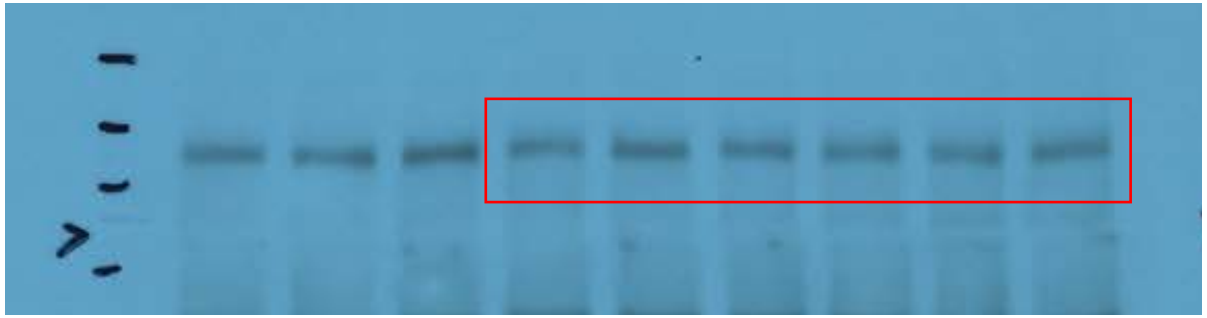

Figure S5C- Nlk

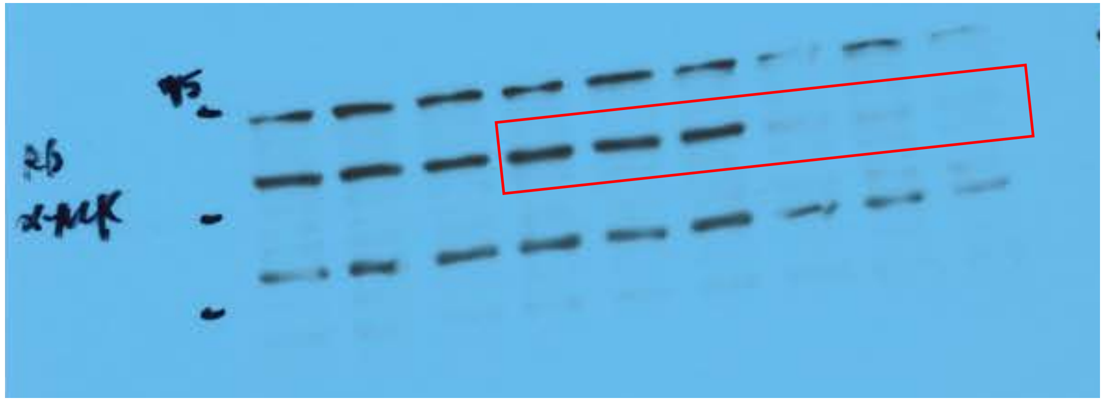

Figure S5C- Actin

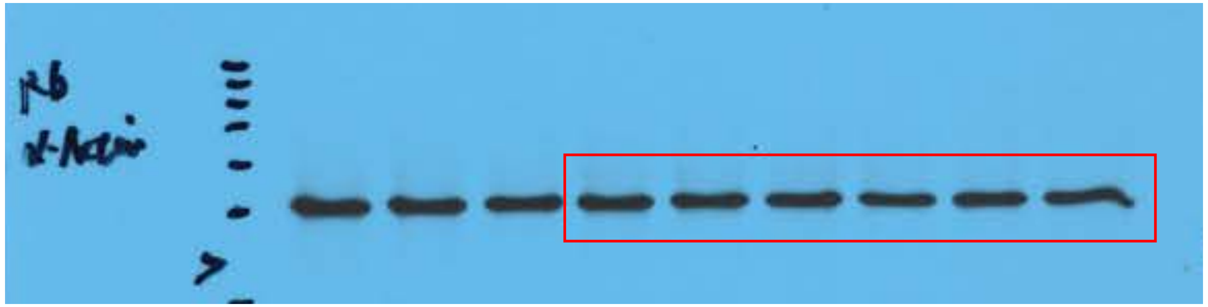

Figure S6A- HA

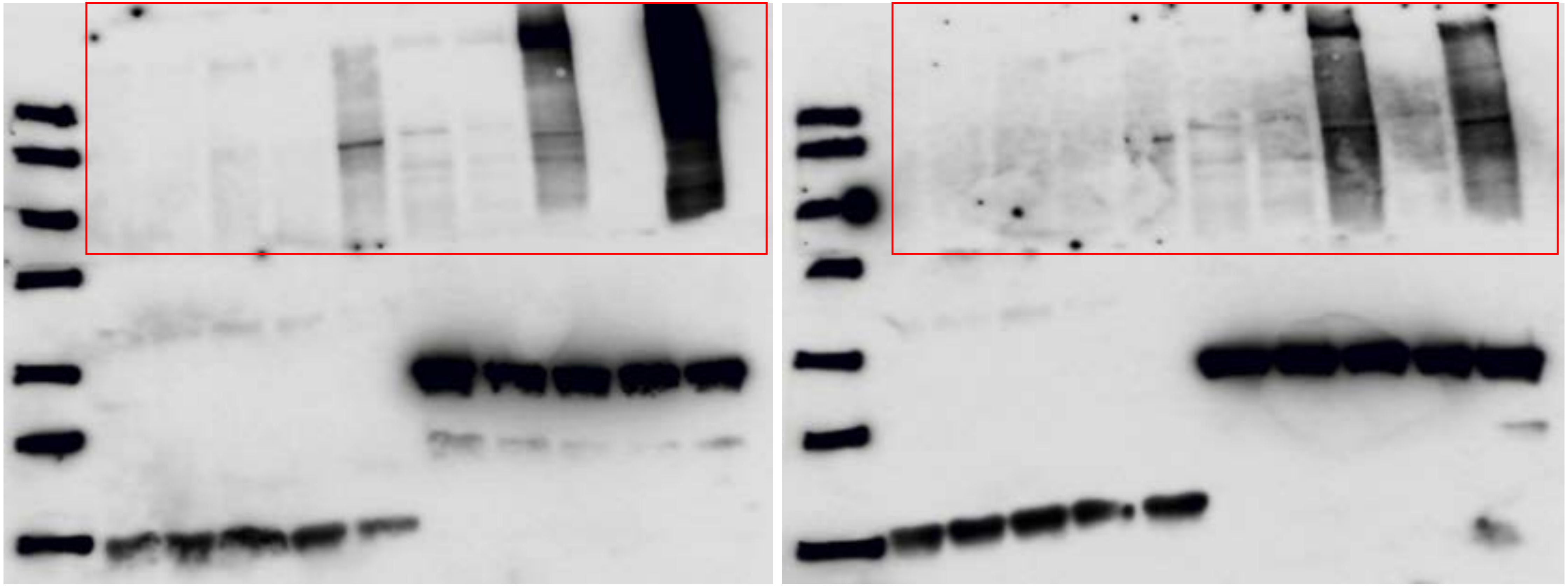

Figure S6A- TDP-43 (high exp.)

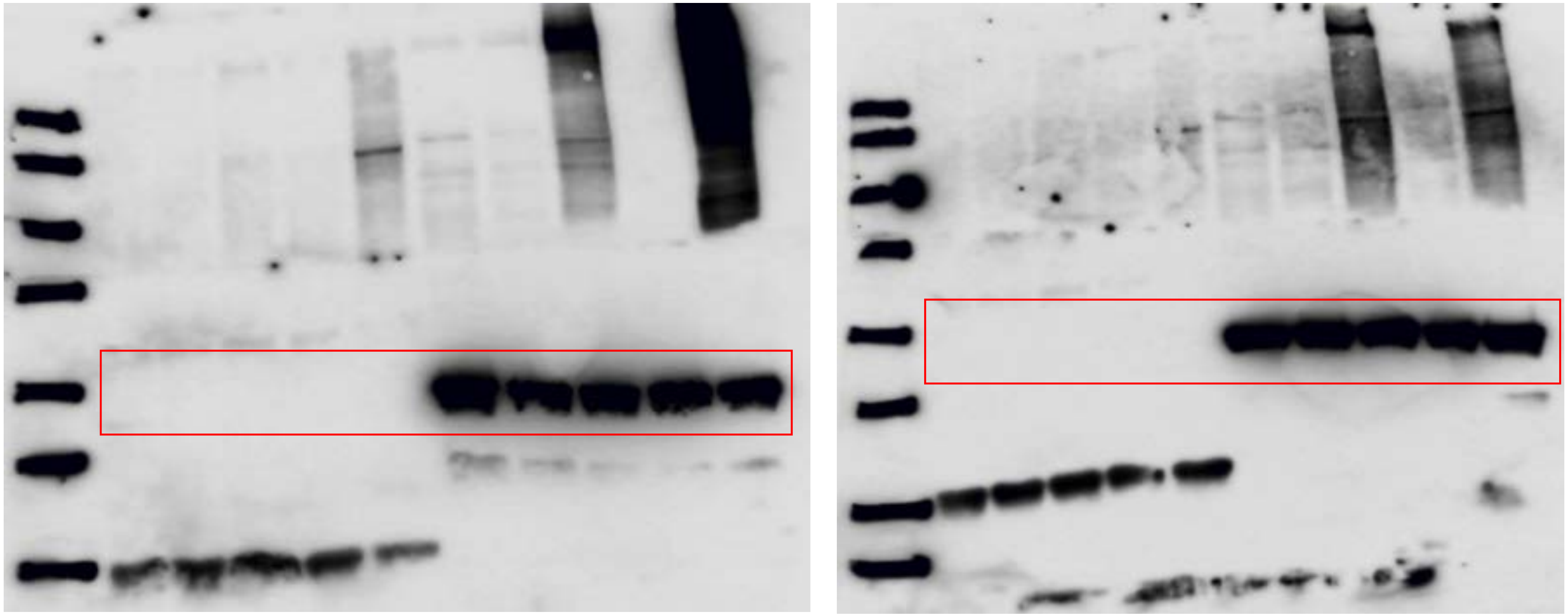

Figure S6A- TDP-43 (low exp.)

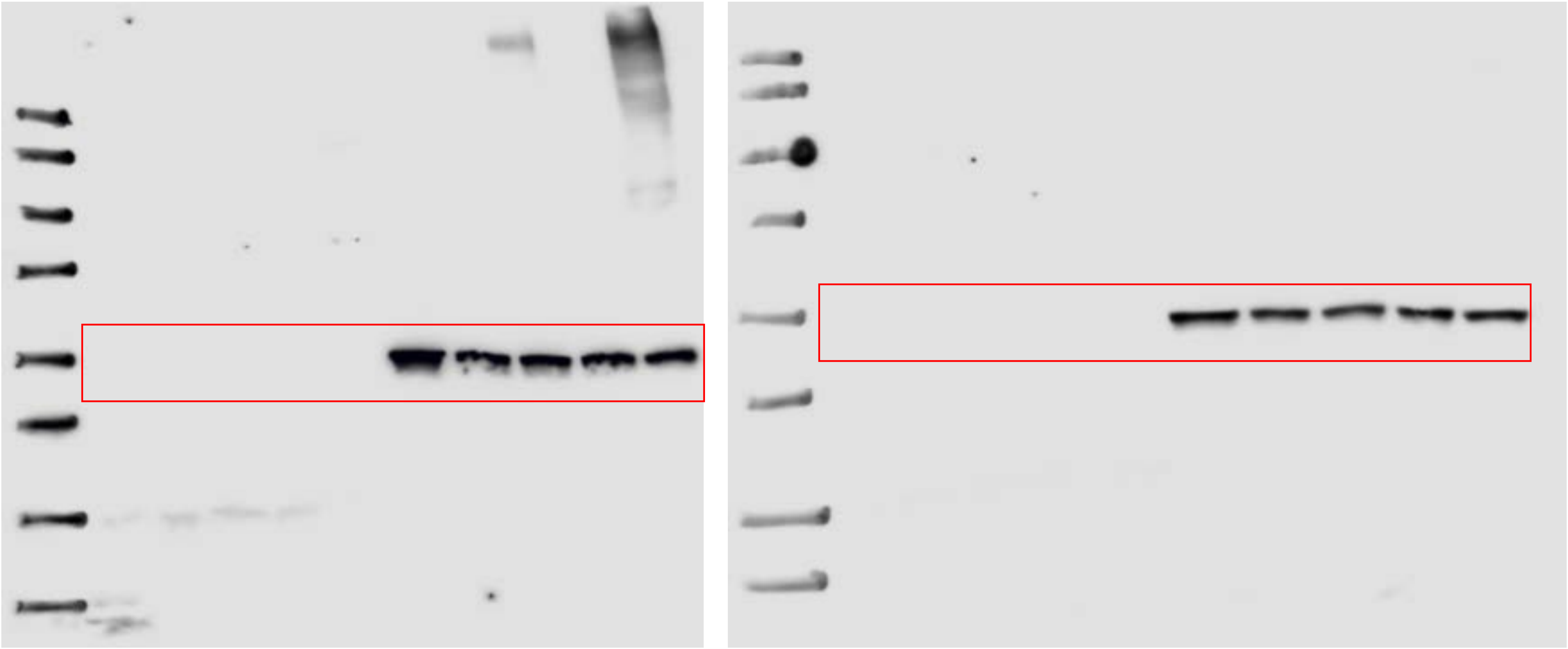

Figure S6A- Nlk

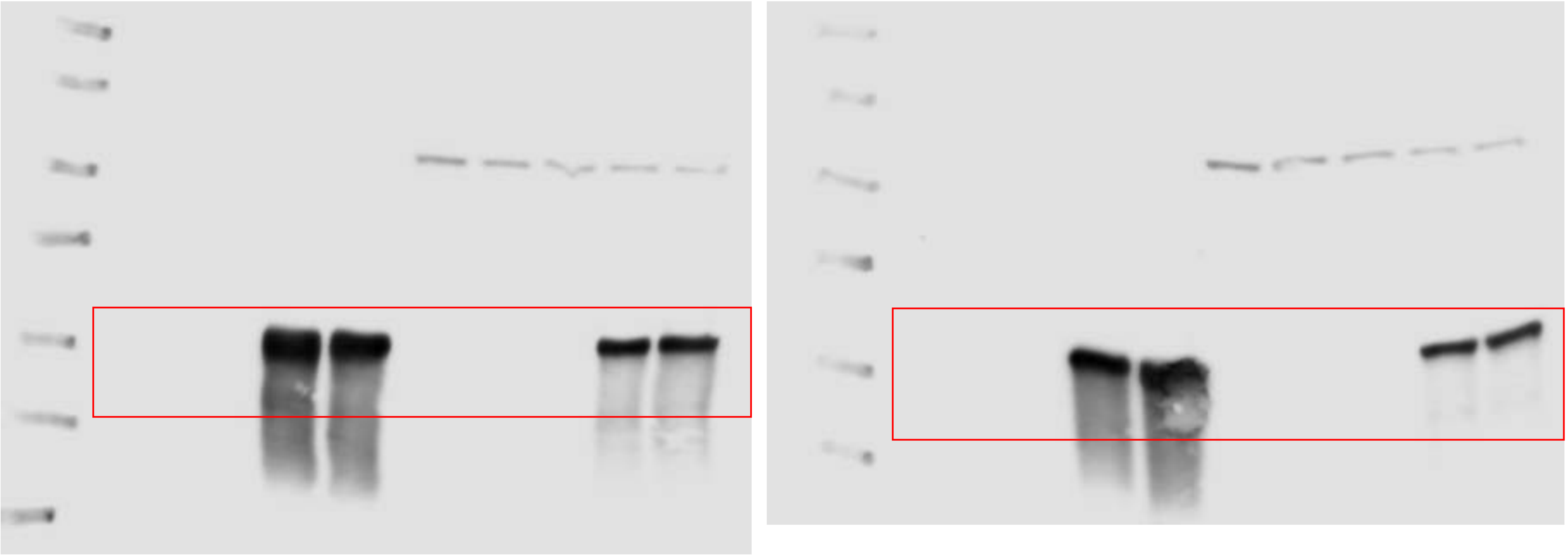

Figure S6A- Vinculin

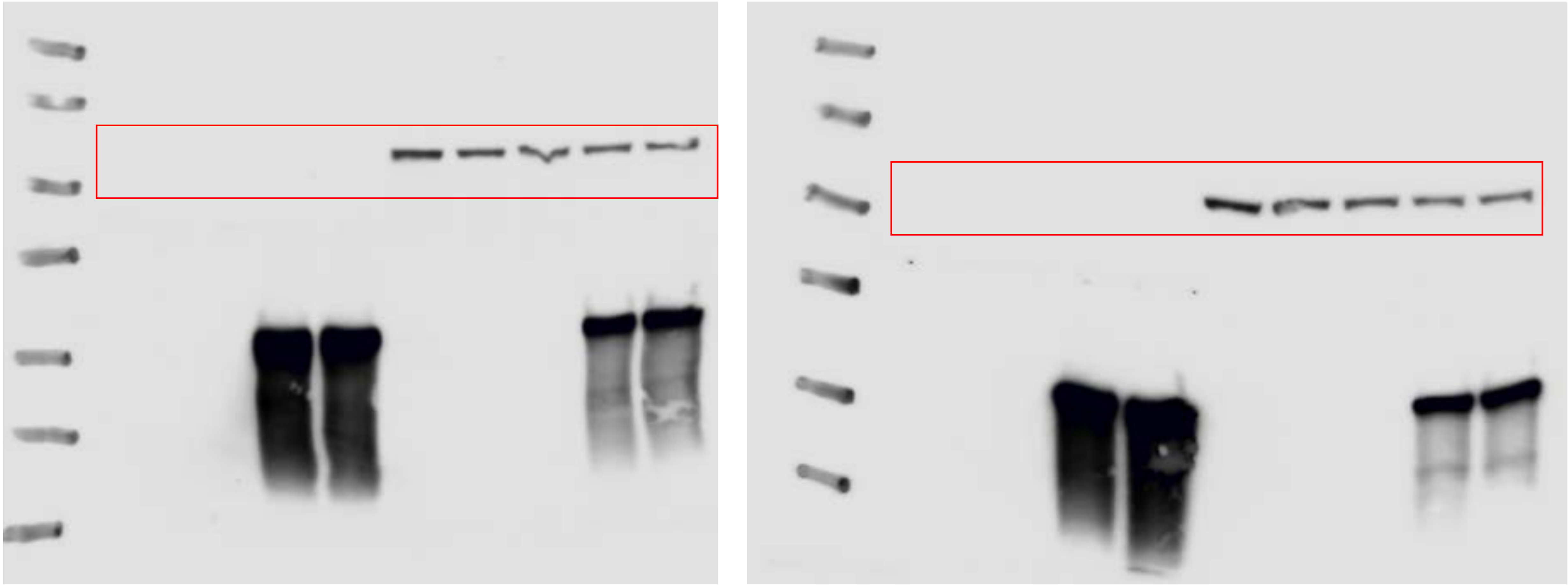

Figure S6B- HA

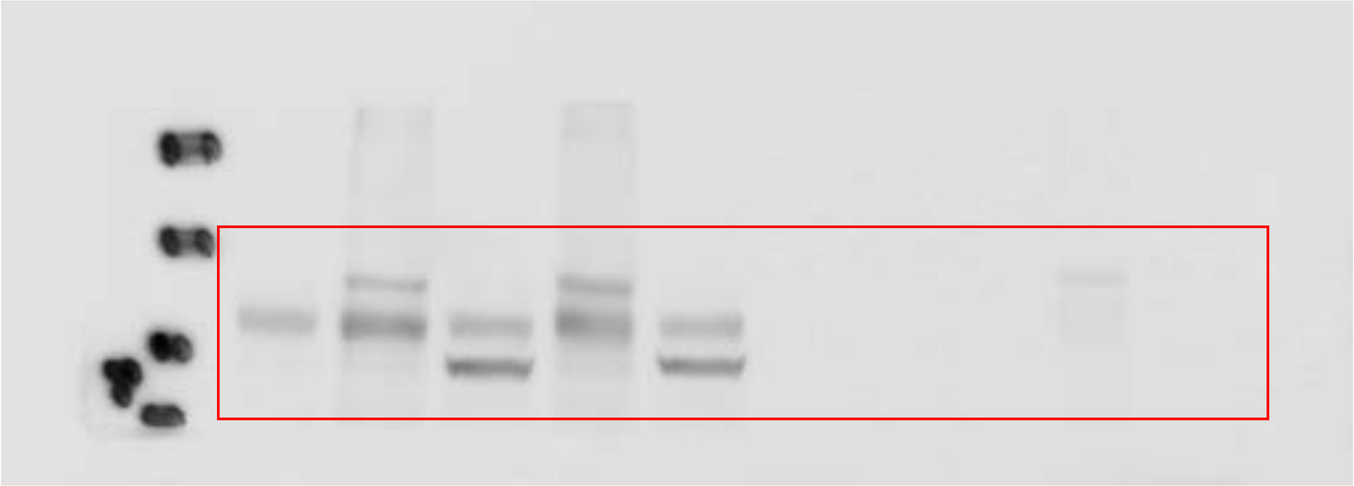

Figure S6B- Flag

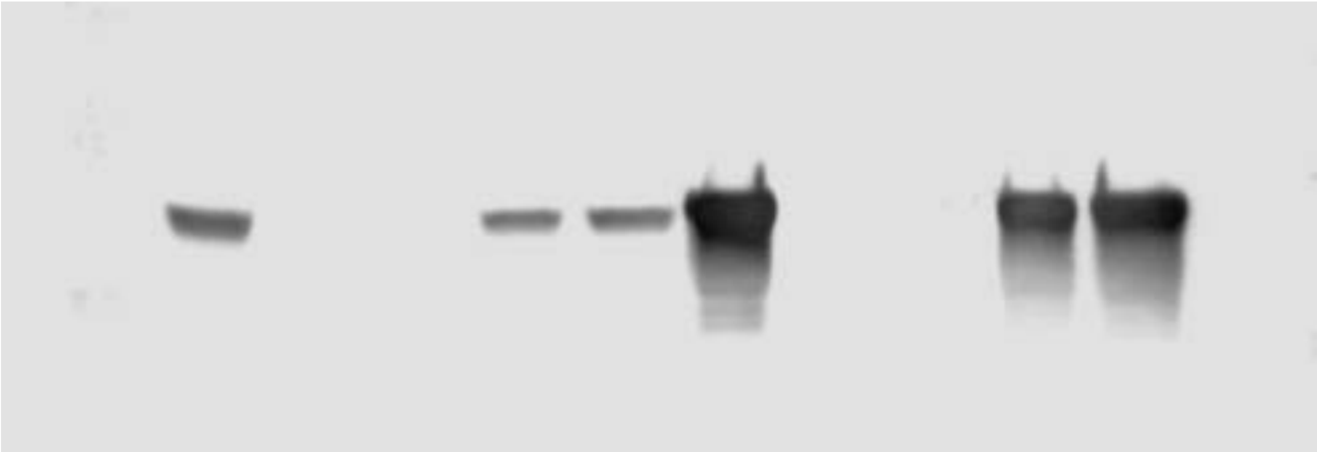

Figure S6B- Gapdh

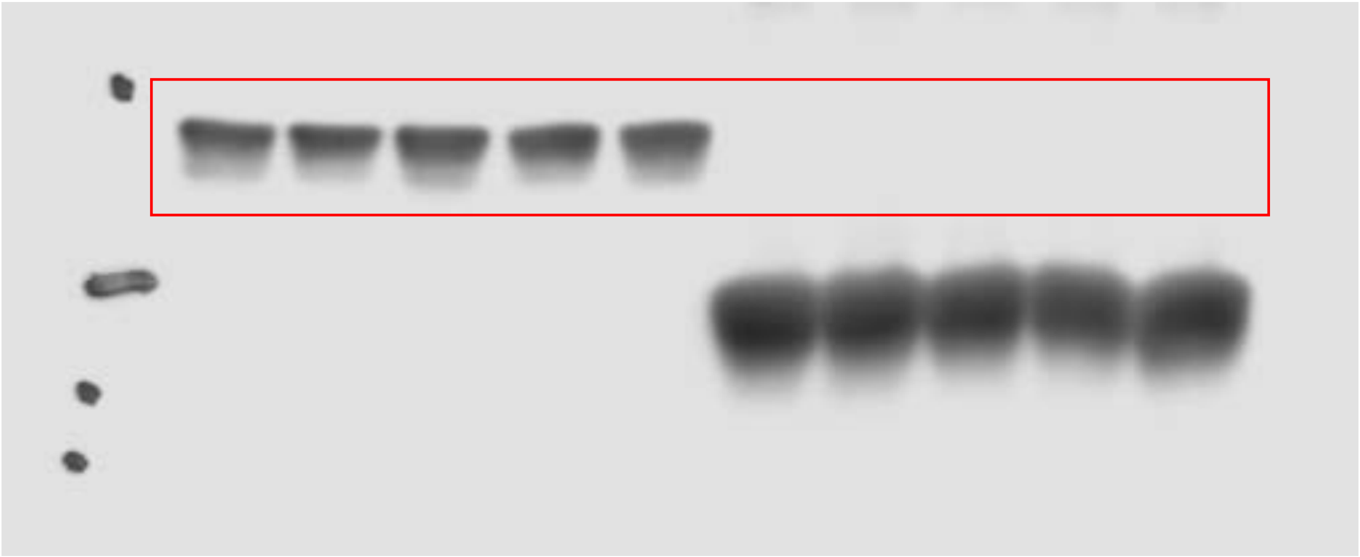

Supplement: Supplemental data [file jci-133-138207-s010.pdf]
